# Towards a platform quantitative systems pharmacology (QSP) model for preclinical to clinical translation of antibody drug conjugates (ADCs)

**DOI:** 10.1007/s10928-023-09884-6

**Published:** 2023-10-03

**Authors:** Bruna Scheuher, Khem Raj Ghusinga, Kimiko McGirr, Maksymilian Nowak, Sheetal Panday, Joshua Apgar, Kalyanasundaram Subramanian, Alison Betts

**Affiliations:** 1 Applied BioMath, 561 Virginia Road, Concord, MA 01742 USA; 2 Present Address: DMPK and Modeling, Takeda, Boston, MA United States; 3 Differentia Bio, Pleasanton, California United States

**Keywords:** Antibody drug conjugate, Quantitative systems pharmacology, HER2, Kadcyla, Enhertu, Oncology

## Abstract

A next generation multiscale quantitative systems pharmacology (QSP) model for antibody drug conjugates (ADCs) is presented, for preclinical to clinical translation of ADC efficacy. Two HER2 ADCs (trastuzumab-DM1 and trastuzumab-DXd) were used for model development, calibration, and validation. The model integrates drug specific experimental data including in vitro cellular disposition data, pharmacokinetic (PK) and tumor growth inhibition (TGI) data for T-DM1 and T-DXd, as well as system specific data such as properties of HER2, tumor growth rates, and volumes. The model incorporates mechanistic detail at the intracellular level, to account for different mechanisms of ADC processing and payload release. It describes the disposition of the ADC, antibody, and payload inside and outside of the tumor, including binding to off-tumor, on-target sinks. The resulting multiscale PK model predicts plasma and tumor concentrations of ADC and payload. Tumor payload concentrations predicted by the model were linked to a TGI model and used to describe responses following ADC administration to xenograft mice. The model was translated to humans and virtual clinical trial simulations were performed that successfully predicted progression free survival response for T-DM1 and T-DXd for the treatment of HER2+ metastatic breast cancer, including differential efficacy based upon HER2 expression status. In conclusion, the presented model is a step toward a platform QSP model and strategy for ADCs, integrating multiple types of data and knowledge to predict ADC efficacy. The model has potential application to facilitate ADC design, lead candidate selection, and clinical dosing schedule optimization.

**Supplementary Information:**

The online version contains supplementary material available at 10.1007/s10928-023-09884-6.

## Introduction

Antibody drug conjugates (ADCs) are a class of targeted therapies for cancer treatment that combine a specific antibody to a tumor antigen linked to a potent cytotoxic agent. The aim of this therapeutic is to target the cytotoxic drug to tumor cells, thus maximizing efficacy whilst minimizing systemic toxicity. ADCs are clinically validated, with currently 14 ADC drug approvals received worldwide for various solid and hematological malignancies [1]. These ADCs deliver chemotherapeutic payloads with diverse mechanisms of action including microtubule inhibitors, DNA cross-linkers and topoisomerase (TOPO) inhibitors. Advancements in conjugation chemistry and linker technology have enabled site specific conjugation of payloads and more stable linkers [2, 3]. Several ADCs have been launched with accelerated approval and have demonstrated transformative responses in the clinic that are broad and deep even in treatment of refractory cancers. In addition, certain tumor payloads can stimulate immune responses, either by direct stimulation of dendritic cell activation and maturation or by triggering immunogenic cell death. As a result, checkpoint inhibitors have emerged as an obvious combination partner for ADCs [4].

However, it has been a significant journey to get to this point, and first-generation ADCs were limited by several challenges in the clinic including severe adverse events and sub-optimal efficacy, unable to reach their efficacious doses before the onset of dose limiting toxicities (DLTs). This is partly because ADCs are not truly targeted; they distribute throughout the body and can be taken up into healthy tissues via target-mediated mechanisms (on-target binding to healthy tissues expressing the ADC target, or via binding to FcγRs), or target-independent mechanisms such as non-specific endocytosis [5]. In addition, ADCs can have poor penetration into solid tumors, which restricts their efficacy [6]. The future success and optimal design of ADCs requires an improved mechanistic understanding to better predict efficacy and toxicity. In this manuscript we have sought to use mechanistic modeling approaches to better predict efficacy, with a second manuscript focusing on ADC toxicity.

Prediction of ADC efficacy through experimental methods alone can be laborious, expensive or even infeasible [7]. Traditional approaches can be misleading, especially when considered in isolation. In vitro cytotoxicity assays provide a single point estimate of an ADC’s potency for a chosen cell line (i.e. IC_50_ or IC_90_) but do not give any information on the dynamics of the response. There are relatively few reports establishing in vitro to in vivo correlations for ADCs [8]. While in vivo mouse tumor xenograft models can be useful to describe efficacy of ADCs, these models are an extreme simplification of human cancer. Species-specific differences in target expression/distribution, ADC PK, tumor penetration, tumor growth rates and heterogeneity need to be considered when translating the results to the clinic. In addition, xenograft mice are more tolerant to ADC toxicities, and dose can simply be increased until efficacy is demonstrated [9].

The inherent complexity of ADCs lends itself well to the use of mathematical modeling and simulation, to map out the mechanism of action and to consider the impact of multiple variables. Mechanistic approaches, such as quantitative systems pharmacology (QSP) models, combine computational modeling and experimental data to examine the relationships between a drug, the biological system and the disease process [10, 11]. Several mathematical models have been published for ADCs over recent years, evolving from empirical and semi-mechanistic PK/PD models, towards more mechanism-based models [7]. These models have been used to describe in vitro assays, preclinical animal experiments, and clinical trials in humans [7]. They have become an important tool to support design of ADCs, to enable preclinical to clinical translation, and facilitate more efficient and effective development of ADCs. However, the models have generally been developed for specific ADCs and there is a need for a more holistic model, with sufficient intracellular detail to describe the mechanism of action of all approved ADCs, incorporating the learnings from previous models. This needs to be accompanied by a strategy for preclinical to clinical translation of ADCs, integrating multiple data types and established knowledge to predict ADC efficacy.

In this manuscript, a next generation multiscale QSP platform model for ADCs is presented, that takes preclinical data routinely collected during ADC discovery and development as input and performs a clinical translation of ADC efficacy. The model was developed, calibrated, and validated using data from the public domain on trastuzumab emtansine (T-DM1) and trastuzumab deruxtecan (T-DXd), two ADCs with the same HER2-targeting antibody (trastuzumab) but having different linker-payloads, intracellular mechanisms of action, and clinical responses. The model describes ADC administration and disposition, including payload release, mechanistic characterization of tumor uptake, binding, and intracellular processing of ADC and payload. A detailed intracellular model with a distinct endo/ lysosomal compartment, receptor recycling, and differentiated release of payload from non-cleavable or cleavable linkers is included. In addition, the model describes the competition between the ADC and the naked antibody for binding to the tumor target. The PK model incorporates physiological volumes for better translation across species and includes the binding of ADC and the naked antibody to the healthy tissue sinks. Tumor payload concentrations predicted by the model were linked to a model of TGI in xenograft mice and then translated to humans to predict outcome via virtual clinical trial simulations. The presented model is a step toward a platform QSP model and strategy for ADCs, integrating multiple types of data and knowledge to predict ADC efficacy.

## Methods

### Model structure

#### In vitro cellular model

**Fig. 1:**
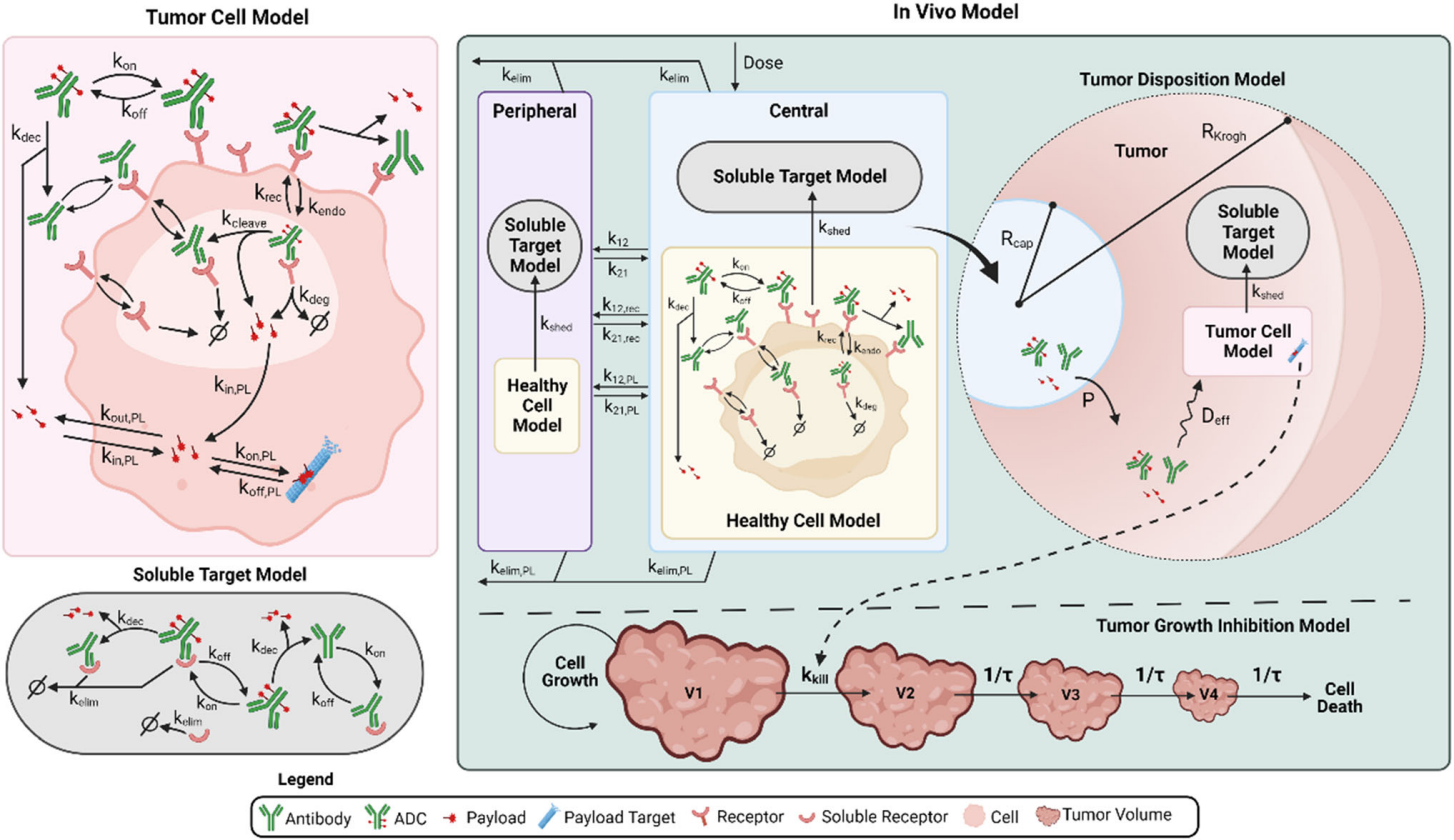
Schematic representation of the ADC QSP model. The full QSP model consists of several submodels connected in a modular fashion. Submodels include **a** Tumor cell model, **b** Soluble target model, **c** Healthy cell model, **d** Tumor disposition model and **e** Tumor growth inhibition model. The full human model is shown. The in vitro cellular model (shown in Figure S1A) and the mouse model (shown in Figure S3) are simplifications of the human model. The model describes the disposition and PK of ADC, antibody and released payload in central, peripheral and tumor compartments. ADC is dosed in the central compartment, where it can deconjugate to release payload and naked antibody or distribute to peripheral and tumor compartments. The ADC and the Ab can bind to receptors expressed on healthy cells in the central and peripheral compartment, receptors expressed on tumor cells in the tumor compartment, and soluble receptors in central, peripheral, and tumor compartments. The ADC, antibody and payload are eliminated in central and peripheral compartments. The model incorporates a mechanistic characterization of tumor uptake, implemented using a Krogh cylinder model. In the tumor the ADC can deconjugate, ADC and Ab can bind to receptors on the surface of the tumor cell and be internalized into the endo/ lysosomal compartment. Here, intracellular bound ADC can either be recycled back to the cell surface, or the payload can be released either by linker cleavage (cleavable linkers) or via ADC degradation (non-cleavable linkers). Payload can escape into the cytosol and bind to its target or exit the cell. Free payload can also re-enter the cell. Tumor payload concentrations predicted by the model were linked to a model of TGI in xenograft mice. The state variables used in the model are described in Table S1, the model parameters are described in Table S2, and the reactions and resulting ordinary differential equations are shown in Table S3

The in vitro model was developed to describe the dynamics that occur at the cellular level in an in vitro culture system (Figure S1A). This model also represents the tumor compartment in the in vivo version of the model (Fig. 1—Tumor Cell Model). The state variables included in the model diagram are defined in Table S1a, the model parameters are included in Table S2a and the reactions and equations in Table S3a and b. The ADC is dosed into the media and can deconjugate in a single step ($k_{dec}$), losing its payload, to produce unconjugated antibody and free payload. The ADC and antibody (Ab) reversibly bind to HER2 receptors on the cell surface ($k_{on,Ab}$, $k_{off,Ab}$) and are internalized into the cells ($k_{endo,HER2}$, $k_{endo, HER2:Ab}$). The binding interaction is modeled as a monovalent interaction. It is assumed that HER2 receptors are at steady-state, and that the total number of HER2 receptors does not change with time. Intracellular bound ADC/Ab can either recycle back to the cell surface ($k_{rec,HER2} , k_{rec, HER2:Ab}$) or degrade ($k_{deg,HER2}$, $k_{deg,HER2:Ab}$) in the endo/lysosomal compartment. Intracellular free payload is released following ADC degradation according to the drug to antibody ratio (DAR), and irreversibly escapes into the cytoplasm ($k_{in,PL}$) where it can reversibly bind to the intracellular payload target ($k_{on,PL, } k_{off,PL}$) or exit the cell ($k_{out,PL}$). Free payload in the media can also (re-)enter the cell ($k_{in,PL}$). There is no first order elimination of ADC, only degradation to release payload. For T-DXd, an additional rate constant was included in the endosomal/ lysosomal compartment describing cleavage of the linker to release the payload ($k_{cleave}$). Transport of released payload from the lysosome to the cytosol was described as a first order process. While this can capture the overall process, it is not mechanistically accurate and therefore a simplification for lys-smcc-DM1, which has been shown to be delivered from the lysosomes to the cytosol via an active transport protein [12, 13]. Free payload exits the cell only by passive diffusion, with no active efflux required for DM1 or DXd.

To assist with parameterization, the model was calibrated to several in vitro datasets. Receptor-mediated cellular uptake was calibrated to a dataset describing incubation of ^125^I-trastuzumab with SK-BR-3 breast cancer cells and measurement of cell surface, dissociated, internalized, and catabolized radioactivity [14]. Internalization, recycling, and degradation rates were fitted using this data, as well as affinity of trastuzumab for HER2 and number of cells in the experiment. The model was validated using data from an independent study where trastuzumab was incubated with BT474 cancer cells and internalization of trastuzumab bound to HER2 was measured [15]. These parameters were then used to model in vitro T-DM1 disposition experiments completed by Erickson et al*.*, whereby T-[H]^3^DM1 was incubated with BT-474EEI, SK-BR-3 and MCF7-neo/HER2 breast cancer cells [16]. In these experiments, total, conjugated and unconjugated DM1 was measured inside cells, unconjugated DM1 was measured in the media and total DM1 metabolites were measured in the incubation. Parameters for the model were informed from calibration to the Austin et al*.* 2004 data, with estimation of extracellular T-DM1 deconjugation rate and numbers of receptors/cell for each cell line to match the intracellular concentrations reported in the data. Observed vs. predicted diagnostic plots for in vitro and in vivo fitting are provided in Figure S2. The final intracellular concentrations were calculated using the reported cellular volumes described in Table S2. In vitro disposition data was not available for T-DXd and its released payload deruxtecan. Since T-DM1 and T-DXd both have trastuzumab as their antibody backbone, the antibody parameters are shared between models. The payload specific parameters for DXd were extracted from the literature (see Table S2) and used directly in the model without calibration.

#### In vivo mouse model development

##### Modeling plasma PK of ADC and payload in mouse and predicting tumor payload concentrations

The mouse PK model combines mouse plasma PK, a mechanistic tumor uptake model and the cellular model described in Sect. 1 above, to predict ADC and payload concentration in the tumor (Figure S3). All of the state variables included in the model diagram are defined in Table S1b and model parameters are included in Table S2b. Trastuzumab does not bind to the rodent version of HER2 (ErbB2/neu) and therefore soluble target and normal tissue expression of target were not required in the mouse model [17]. ADC is dosed into the central compartment and can distribute to peripheral tissues ($$k_{12}$$,$$k_{21}$$) or to the tumor. ADC can deconjugate ($$k_{dec}$$) in any compartment, releasing Ab and its payload (as a function of the DAR). Payload can also distribute to peripheral tissues ($$k_{12,PL}$$, $$k_{21,PL}$$). ADC, Ab, and payload are cleared by first order elimination ($$k_{elim}$$*)* in central and peripheral compartments. Distribution of ADC, Ab, and payload from the central compartment into a solid tumor occurs through surface exchange $$\left( {\frac{6*D}{{R_{tumor}^{2} }}} \right)$$ when the tumor is small, and vascular exchange described using a Krogh cylinder model $$\left( {\frac{{2*P*R_{cap} }}{{R_{krogh}^{2} }}} \right)$$ at larger tumor sizes [18–20]. The tumor void fraction (ε) represents the tumor volume that is accessible to ADC, Ab, or payload. There are different ε values for ADC/Ab and payload (based on MW). In the tumor extracellular environment, cellular disposition of ADC, Ab and payload are characterized using the parameters and model described in Sect. 1 (Figure S3—Tumor Cell Model). Physiological volumes were used for central, peripheral, and tumor compartments in mice [21]. The T-DM1 and T-DXd mouse PK models were calibrated to plasma PK data in non-tumor bearing mice following IV administration at 3 mg/kg, with measurement of ADC and total antibody concentrations over time (Figure S4) [16, 22]. The model was used to fit the data for both ADCs simultaneously. It was assumed that the ADC distribution and half-life parameters were the same for both T-DM1 and T-DXd, but the deconjugation rate was allowed to differ for each ADC. The model was also used to fit plasma PK data following IV administration of DXd at 1 mg/kg to non-tumor bearing mice, in order to estimate payload distribution and clearance rates [22]. Comparable data was not available for T-DM1, however mouse PK data from IV administration of a maytansine with structural similarity to DM1 was available (100 µg/kg dose) which was used to estimate the payload half-life [23]. For parsimony, distribution parameters were assumed to be the same for DM1 and DXd.

The model was then used to simulate data from an in vivo tumor disposition study describing T-[H]^3^-DM1 administration at 300 µg/kg to BT-474EEI tumor-bearing xenograft mice with measurement of DM1 in both plasma and tumor [16]. The reactions and equations for the integrated mouse PK and tumor uptake model are included in Table S3c and Table S3d.

##### Modeling tumor growth inhibition in mouse

Tumor growth and killing were implemented to capture ADC treatment response in tumor growth inhibition studies in xenograft mice across various human cell lines (Fig. 4 and S5). As before, the state variables included in the model diagram are defined in Table S1b and model parameters are included in Table S2c. The TGI model used has been described previously [24]. The tumor growth model describes both exponential tumor doubling time ($t_{double}$) and linear tumor growth ($k_{lin}$), limited by the maximum tumor volume ($V_{tumor, max}$). ψ is an arbitrary constant for switching from exponential to linear growth patterns set to a value of 20 [25]. 4 tumor cell states (N1, N2, N3, N4) are included in the model, representing the proliferating cell mass (N1) and the terminally committed cells in a transduction cascade (N2 → N3 → N4). Tumor killing is described with a Michaelis–Menten type model acting on the proliferating cells (N1) and is dependent on the intracellular unconjugated tumor payload concentration. The total tumor volume (mm^3^) is the sum of all cells in the transduction cascade (N1 + N2 + N3 + N4). N2, N3 and N4 do not proliferate, but otherwise are similar to N1 in terms of receptor expression, binding, endocytosis, and recycling. The transduction cascade introduces a delay to the tumor cell killing (τ). The maximal killing rate ($k_{kill, max}$) and concentration of payload at half-maximal kill rate ($kc_{50}$) are estimated by fitting the model to mouse xenograft data.

The mouse efficacy model was calibrated to TGI data for T-DM1 versus N87 [24, 26], BT-474 [27], BT474EEI [16], and KPL-4 tumor cell lines [28], and for T-DXd versus N87 tumor cell line [29], following a range of doses. The reactions and equations for the mouse tumor growth inhibition model are included in Table S3c and Table S3d.

#### Human model development and analysis

##### Modeling the PK of ADC, total Ab, and payload in the plasma of cancer patients

The human PK model captures the disposition and PK of the ADC, the Ab and the payload in the central, peripheral and tumor compartments in HER2+ cancer patients (Fig. 1). State variables included in the model diagram are defined in Table S1 and model parameters are included in Table S2d. ADC is dosed in the central compartment, where it can deconjugate or distribute to peripheral and tumor compartments as described in the mouse model. All compartments have human physiological volumes [21]. In the human model, the ADC and the Ab can also bind to soluble HER2 receptors in central, peripheral, and tumor compartments. In addition, the ADC and the Ab can bind to HER2 receptors on healthy cells in the central and peripheral compartment as well as HER2 receptors expressed on tumor cells in the tumor compartment ($$k_{on, Ab}$$*,* $$k_{off, Ab}$$). HER2 receptors are shed ($$k_{shed}$$) to form soluble HER2 receptors and soluble HER2 receptor complexes with the ADC and the Ab, which then can also be degraded/eliminated ($$t_{{\raise.5ex\hbox{$\scriptstyle 1$}\kern-.1em/ \kern-.15em\lower.25ex\hbox{$\scriptstyle 2$} , sHER2}} , t_{{\raise.5ex\hbox{$\scriptstyle 1$}\kern-.1em/ \kern-.15em\lower.25ex\hbox{$\scriptstyle 2$} , sHER2:Ab}}$$) [30]. Soluble HER2 and its complexes can distribute between the central compartment and peripheral or tumor compartments. Soluble target shedding is linked to tumor size via membrane HER2 expression. The parameters for the ADC human PK model were informed from literature data or were carried through from the in vitro model fitting (see Table S2d). The human PK model was used to simulate ADC, total antibody and payload PK and compared to data from phase 1 clinical studies. For T-DM1, the model was used to simulate data following IV infusion to HER2+ metastatic breast cancer (MBC) patients at doses ranging from 0.3 to 4.8 mg/kg Q3W. ADC concentrations were measured during the first dose dosing cycle for all doses, and total antibody and released payload levels were reported at the MTD of 3.6 mg/kg [31]. For T-DXd, the model was used to simulate data from patients with breast, gastric or gastro-oesophageal carcinomas, who received IV doses of T-DXd from 0.8 to 8.0 mg/kg Q3W. ADC levels were measured at all doses for 3 dosing cycles. In addition, total antibody and released deruxtecan were measured at 6.4 mg/kg over cycles 1–3 [32].

##### Predicting tumor growth inhibition in HER2+ metastatic breast cancer patients

**Fig. 2:**
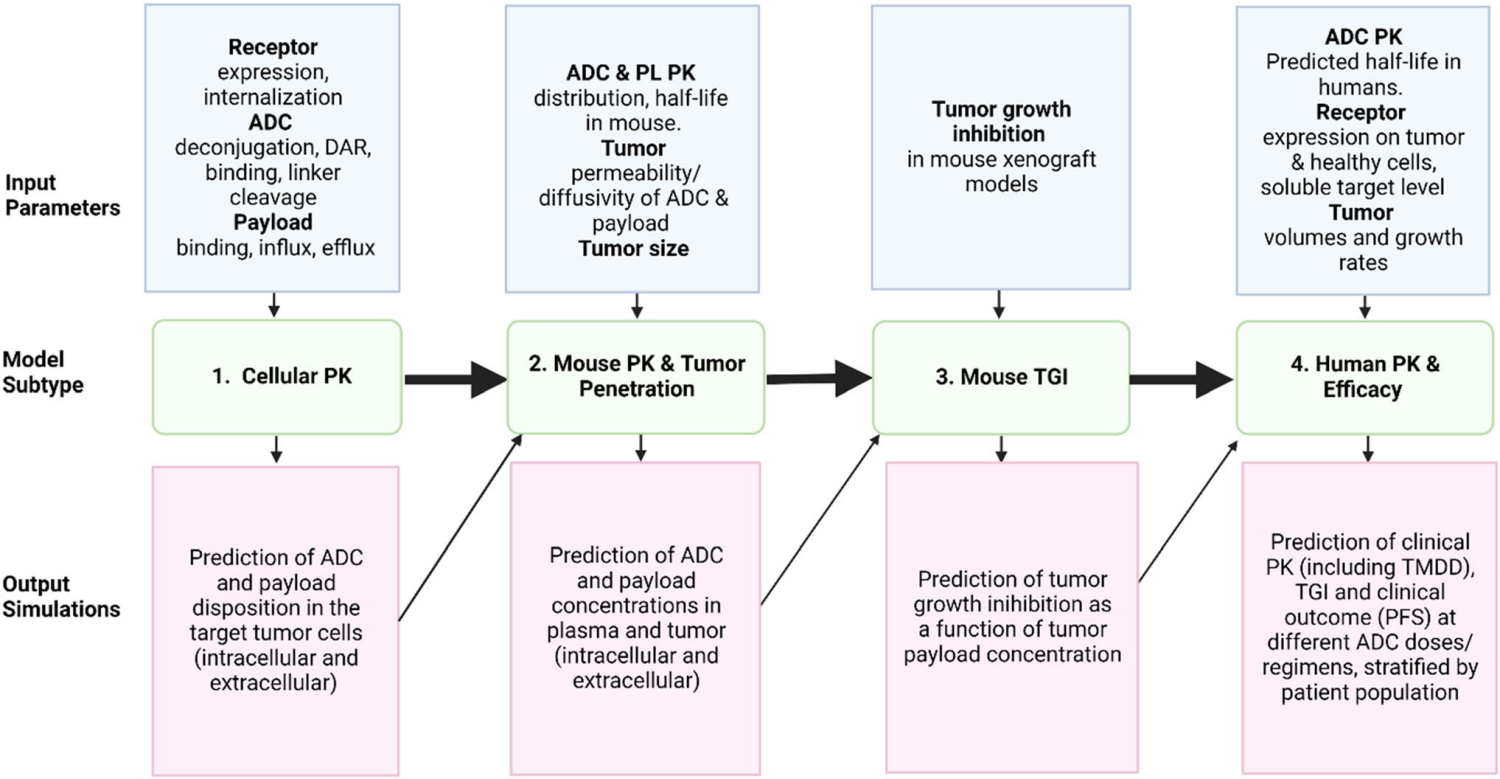
Workflow of ADC QSP model development and translational strategy, connecting in vitro, in vivo, and clinical data, models, and predictions. At each step, the model integrates the input data and knowledge of the biological mechanism to perform simulations and predictions which informs the next step in the strategy

The calibrated human PK model for T-DM1 and T-DXd was used to predict tumor payload concentrations, and intracellular unconjugated tumor payload concentrations were linked to TGI as described for the mouse model (Table S2). The human model was parameterized for HER2+ MBC as follows: (a) clinically observed exponential and linear growth rates for HER2+ MBC were used in the human model [33, 34], (b) HER2 expression levels of 20,000 and 1 million receptors per cell were simulated, approximating HER2 1+ and 3+ patients [35] and (c) initial and maximal tumor burdens were set to clinically observed/ plausible values [36, 37]. The drug effect parameters, including the transduction delay, maximum kill rate, and concentrations at half max kill rates were taken directly from mouse model estimations (Fig. 2). The translational strategy is illustrated in Fig. 2. Please refer to Table S2d for parameter values.

##### PRCC and virtual clinical trial simulations

Global sensitivity analysis was completed using a sampling-based method (latin hypercube partial rank correlation coefficient or LHS-PRCC) to identify the most sensitive parameters impacting the tumor volume response following ADC administration [38]. Parameters were chosen for analysis based on parameter correlation and certainty of parameter values. Parameter ranges and distributions for PRCC sampling were determined by literature values or bounded ten-fold higher and lower of the mean (human parameter values resulting from T-DM1 PK calibration). Sampled parameter sets (n = 1000) were dosed with T-DM1 at 3.6 mg/kg Q3W four times and tumor volume AUC was the output used for determined partial correlation. This analysis was used to guide the selection of parameters to vary for the virtual clinical trial simulations.

Using the results from the global sensitivity analysis, exponential doubling time ($t_{double}$), linear growth rate ($k_{lin}$*),* maximal kill rate ($k_{kill, max}$), and concentration at half maximal killing rate ($kc_{50}$) were selected as the parameters to vary in the creation of the virtual population. The pharmacodynamic killing parameters were varied according to the range estimated from the mouse models (Table S2c), and clinical growth parameters were taken from the literature [33, 34]. The virtual patient simulations for T-DM1 were completed to emulate the phase 2 trial by Burris et al.[39]. Specifically, model simulations were performed for 95 HER2+ MBC patients administered 3.6 mg/kg T-DM1 every 3 weeks for 14 months. Patients with high (1e6) and low (2e4) HER2+ receptor expression were simulated and the results from each group were analyzed separately. The virtual patient simulations for T-DXd were completed to emulate a phase 2 trial [40] and a phase 3 trial [41]. To simulate the phase 2 trial, 184 HER2+ patients were administered T-DXd at 5.4 mg/kg Q3W for 20 months. To simulate the phase 3 trial, 373 patients who had low expression of HER2 were administered T-DXd at 5.4 mg/kg Q3W for 29 months. Coefficients of variation for each sampled parameter were calibrated to match the PD observed in the clinical trial data (Table S2e). While the coefficients of variation (CV) for $t_{double}$*,* $k_{lin}$*,* and $k_{kill, max}$ were low, high $kc_{50}$ CVs were required for both ADCs to describe variability in efficacy observed.

### Progression free survival (PFS) predictions

The virtual clinical trial simulations for T-DM1 and T-DXd in HER2+ MBC patients were used to predict PFS based on Response Evaluation Criteria In Solid Tumors (RECIST) guidelines [42]. PFS values were calculated from simulated tumor diameters at different timepoints over the length of the trial. Responses were categorized as follows (i) complete response (tumor diameter < 10 mm), (ii) partial response (> 30% decrease in tumor diameter relative to baseline), (iii) stable disease (< 30% decrease and < 20% increase in tumor diameter from baseline), and iv) progressive disease (> 20% increase in tumor diameter relative to baseline and absolute increase in tumor diameter of > 5 mm). Observation of progressive disease at a given time point was defined as an event. Patient survival rate at each time interval was calculated using following as described by Rich and coworkers [43]:

$$Survival \, rate_{t} = \frac{number\, of\, at-risk\, patients\, after\, event}{{number\, of\, at - risk\, patients\, during\, time\, interval}}$$

where the time interval is defined by when an event occurred. PFS probability (%) was calculated as:

$$Survival\, probability \left( \% \right) = Survival\, rate_{t} * Survival\, rate_{t - 1}$$

Model predicted PFS was compared to clinical trial data published for T-DM1 [39] and T-DXd [40, 41] as described above. Patients removed from the trial due to reasons other than tumor progression were censored by sampling patient drop out times from an exponential distribution, assuming that each individual dropped out of the trial independent of other individuals. Sampling rates, intervals, and number of patients sampled were selected to mimic those observed in the clinical trials.

### Data sources and model implementation

All the data used in model parameterization was extracted from the literature. Datasets used for model calibration and validation were digitized from the literature using WebPlotDigitizer (https://automeris.io/WebPlotDigitizer, version 4.6, Pacifica, CA). Models were fit to data using gradient-descent methods and simulations were performed using QSP Notebook software (Applied Biomath LLC, Concord MA). As per Applied BioMath™ internal guidance, model QC was completed by an independent modeler, not involved in model development. Figures for the manuscript were generated using Biorender.com and plots using matplotlib [44].

## Results

### Modeling the cellular disposition of T-DM1 and lys-smcc-DM1 in cancer cells

**Fig. 3:**
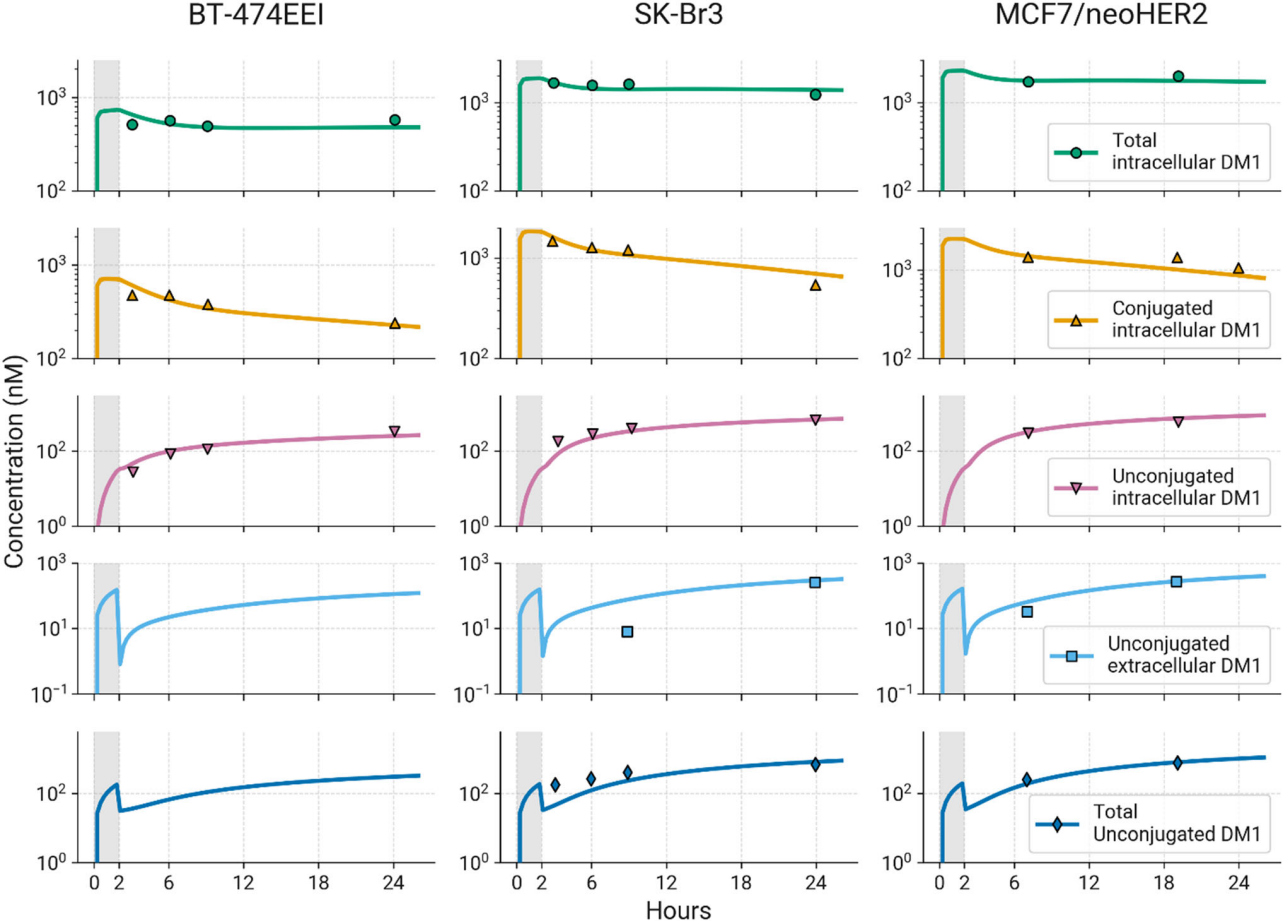
Observed (symbols) and in vitro cellular model calibrated (lines) T-DM1 in vitro disposition data. BT-474EEI, SK-BR-3 and MCF-7-neo/HER2 cells were incubated with T-[^3^H]DM1 for 2 h on ice (gray shaded area), washed, and intracellular and extracellular concentrations of DM1 catabolites were measured [16]. The in vitro cellular model was used to simulate these concentrations and compared to observed data

A cellular model was developed to characterize the in vitro disposition of T-DM1 and its released payload in target tumor cells. Model simulations versus experimental observations for the model calibration and validation to trastuzumab uptake into cancer cells [14, 14, 15] are shown in the supplementary material (Figure S1B). Simulated profiles of T-DM1 incubations in BT-474EEI, SK-BR-3 and MCF7-neo/HER2 breast cancer cells are compared with observed results from this experiment [16] in Fig. 3 and the model parameters are shown in Table S2a. The model predicted concentration versus time profiles of intracellular and extracellular species for all cell lines very well. Consistent with observed data, model simulations showed that concentrations of unconjugated DM1 were higher inside cells than in the media, which is likely due to T-DM1 binding to tubulin intracellularly and its slow diffusion out of cells.

### Modeling the mouse PK of T-DM1 and T-DXd and predicting tumor payload concentrations of T-DM1

The ADC and total antibody plasma PK data for both T-DM1 and T-DXd in non-tumor bearing mice [16, 22] were described well by the model (Figure S4). The model was able to fit the data for both ADCs simultaneously using the same distribution parameters and antibody half-life (11.6 days). The deconjugation rate was estimated separately for the two ADCs and was much slower for T-DXd compared to T-DM1, indicating a more stable linker in circulation. The half-life values for DM1 and DXd were estimated to be 3.5 h and 0.8 h, respectively, from PK data for a maytansine with structural similarity to DM1, and DXd in non-tumor bearing mice.

**Fig. 4:**
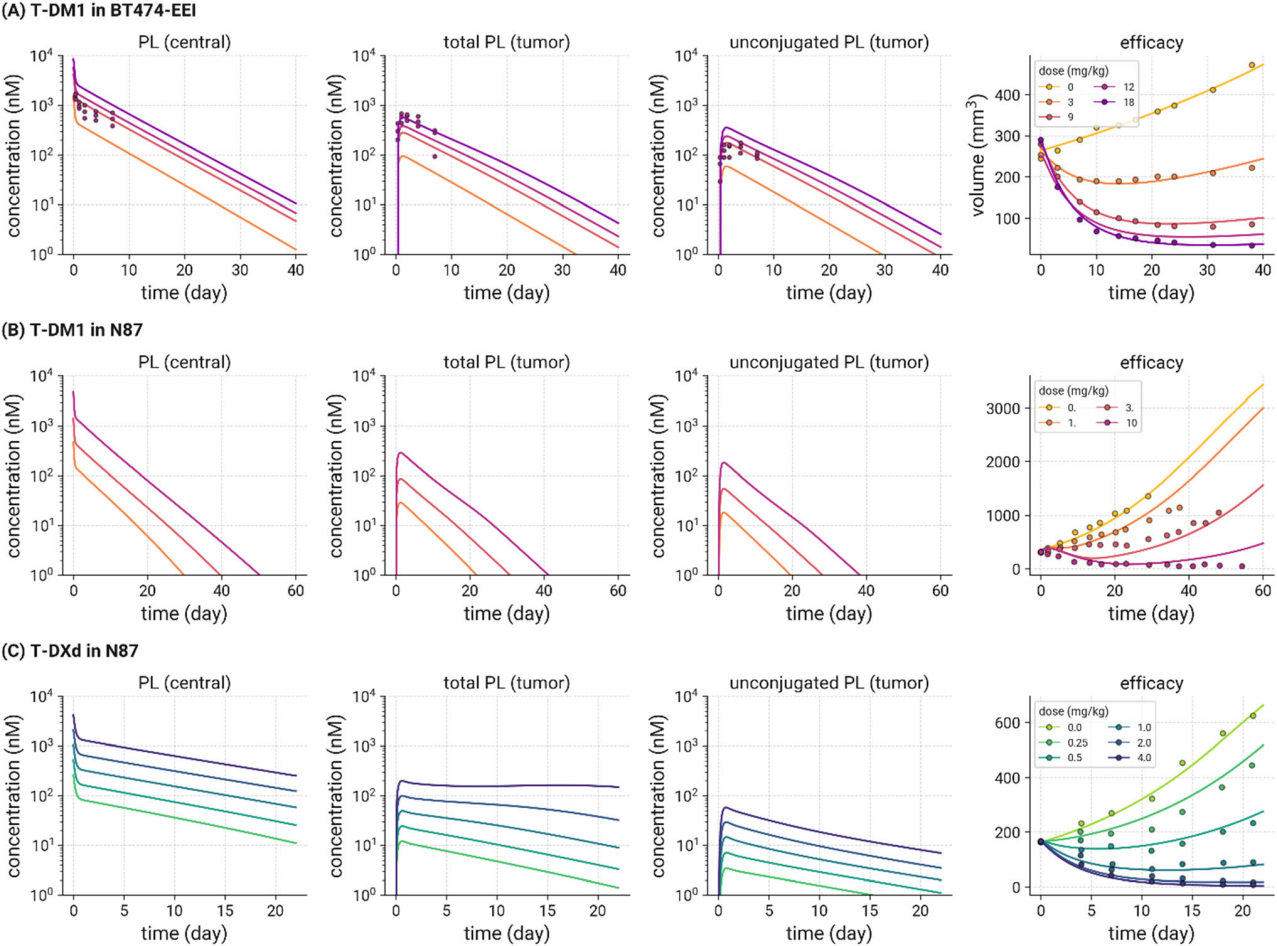
In vivo mouse model calibration for T-DM1 and T-DXd. **A** Observed (symbols) and in vivo PK model calibrated (lines) DM1 disposition data. Following iv administration of T-[H]^3^-DM1 (300 µg/kg DM1 based dose) to BT-474EEI tumor bearing xenograft mice, levels of DM1 were measured in plasma and tumor [16]. Observed (symbols) and in vivo model calibrated (lines) tumor growth inhibition following IV administration of **A** T-DM1 to BT474-EEI, **B** T-DM1 to N87 and **C** T-DXd to N87 mouse xenograft studies. Model simulations of predicted payload concentrations in plasma and tumor (total and unconjugated), and model predicted TGI versus observed data are shown for T-DM1 and T-DXd [16, 24, 26–29]

The mouse plasma PK model for T-DM1 was combined with a mechanistic tumor uptake model and the cellular model to predict tumor concentrations of DM1 in xenograft mice. Figure 4A shows fitting of the model to observed data for DM1 concentration versus time profiles in plasma and tumor of BT-474EEI bearing xenograft mice, after IV administration of T-[H]^3^-DM1 (300 µg/kg DM1 based dose) [16]. Mouse PK parameters are summarized in Table S2b.

### Modeling tumor growth inhibition in mouse following administration of T-DM1 and T-DXd

The predicted intracellular tumor payload concentrations were linked to a model of tumor growth and tumor cell killing and used to fit TGI data from a range of xenograft mouse experiments. Figure 4 shows the fitting of the model to TGI data from a BT474-EEI mouse xenograft study following dosing of T-DM1, and N87 mouse xenograft studies following dosing of T-DM1 and T-DXd. Model simulations of predicted payload concentrations in plasma and tumor, and model calibrated TGI versus observed data are shown for each dataset. Additional figures showing model fits to TGI data from BT-474 and KPL-4 mouse xenograft studies following different doses of T-DM1 are shown in Figure S5. In each case, the model was able to characterize the data well, estimating only parameters associated with tumor cell growth and tumor cell killing (see Table S2c). This demonstrates the flexibility of the model in capturing data from diverse xenograft data, with different tumor growth rates across cell lines. The model parameters (Table S2c) provided a set of PD parameters for preclinical to clinical translation of T-DM1 and T-DXd efficacy.

### Predicting PK of T-DM1 and T-DXd and their released payloads in the plasma of cancer patients

**Fig. 5:**
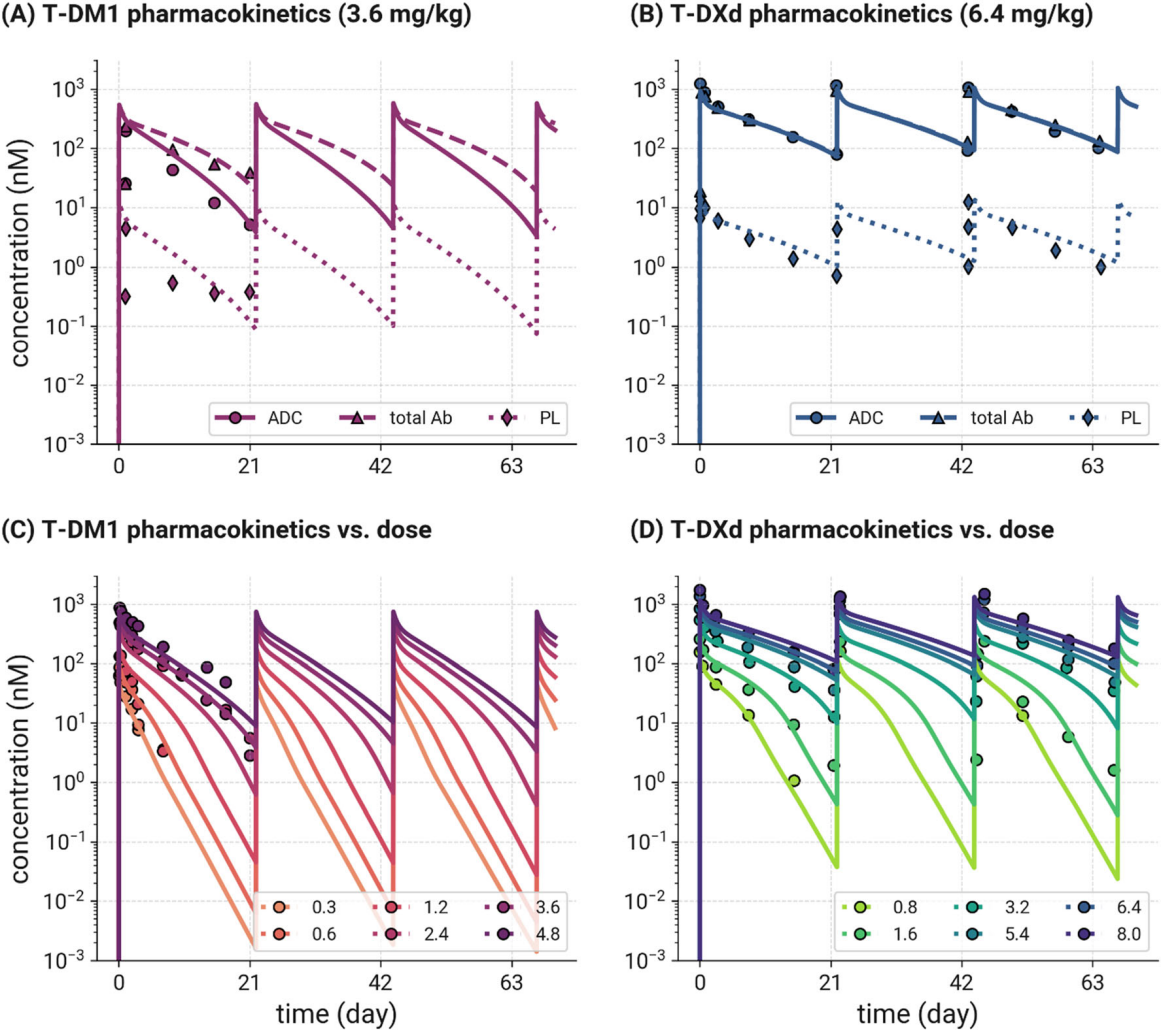
Observed (symbols) and model predicted (lines) clinical PK of T-DM1 and T-DXd. Simulations of ADC, total antibody and released payload following IV administration of **A** T-DM1 to HER2+ MBC patients at 3.6 mg/kg, compared with clinical data from cycle 1 [31], and **B** T-DXd to HER2+ patients with breast, gastric or gastro-oesophageal carcinomas at 6.4 mg/kg, compared to data from cycles 1–3 [32]. Simulations of ADC concentrations in the same patient populations following IV administration of **C** T-DM1 from 0.3 to 4.8 mg/kg and **D** T-DXd from 0.8 to 8.0 mg/kg Q3W, with comparison to clinical data

The human PK model was able to predict the clinical plasma PK data for T-DM1 and T-DXd for all analytes (ADC, total antibody, and payload; Fig. 5) without estimation of any parameters. The model was able to capture the nonlinearity of the ADC concentrations in the dose escalation data due to target mediated drug disposition (Fig. 5C and D). PK model parameters (Table S2d) were used in subsequent clinical TGI and PFS simulations.

### Predicting tumor growth inhibition following administration of T-DM1 and T-DXd to HER2+ MBC patients

**Fig. 6:**
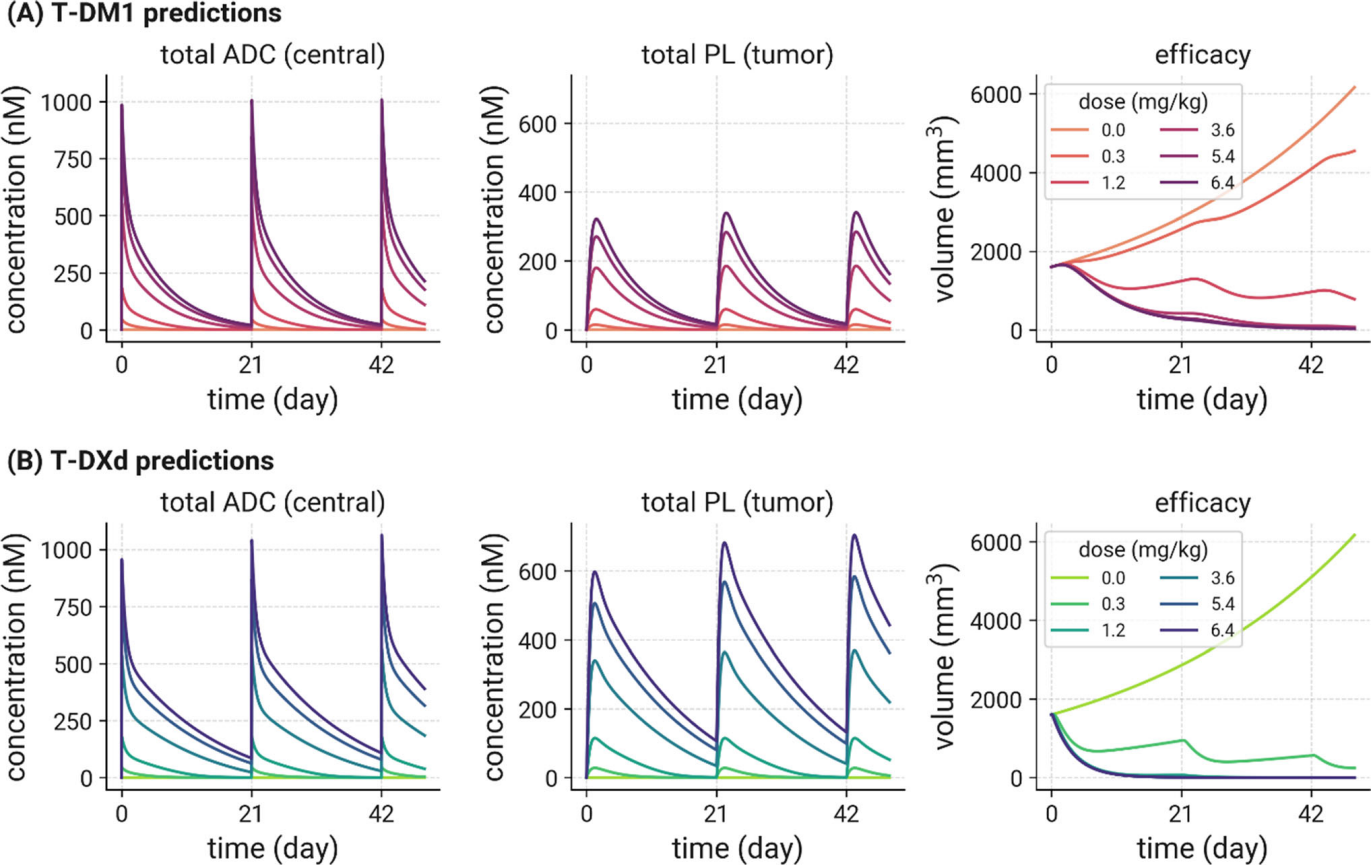
Model predictions of T-DM1 and T-DXd plasma PK, tumor payload concentrations and efficacy (tumor growth inhibition) using nominal patient parameters, following IV administration at 0.3–6.4 mg/kg. T-DM1 and T-DXd have very similar ADC plasma PK, but T-DXd delivers higher concentrations of payload to the tumor, one of the factors that could result in greater predicted efficacy compared to T-DM1 at the same doses

Human model simulations of T-DM1 and T-DXd plasma PK, total tumor payload concentrations and TGI following IV administration at 0.3–6.4 mg/kg are shown in Fig. 6. The simulations predicted that T-DM1 and T-DXd have very similar ADC plasma PK, but T-DXd delivers higher concentrations of payload to the tumor, which contributes to greater predicted efficacy compared to T-DM1 at the same doses.

### PRCC sensitivity analysis

A global sensitivity analysis was performed using the T-DM1-parameterized human model to understand which parameters impacted tumor volume response. The most sensitive parameters (Figure S6) included exponential tumor doubling time ($t_{double}$) and a combination of pharmacodynamic parameters (tumor death transduction delay (τ), maximum kill rate ($k_{kill, max}$), and payload concentration at half maximum kill rate ($kc_{50}$). Other notable sensitive parameters included target related parameters such as soluble HER2 half-life and number of HER2+ cells in normal tissues. Drug parameters including DAR, ADC/Ab permeability and half-life were also sensitive.

### Virtual clinical trial simulations to predict progression free survival of T-DM1 and T-DXd in HER2+ MBC patients

**Fig. 7:**
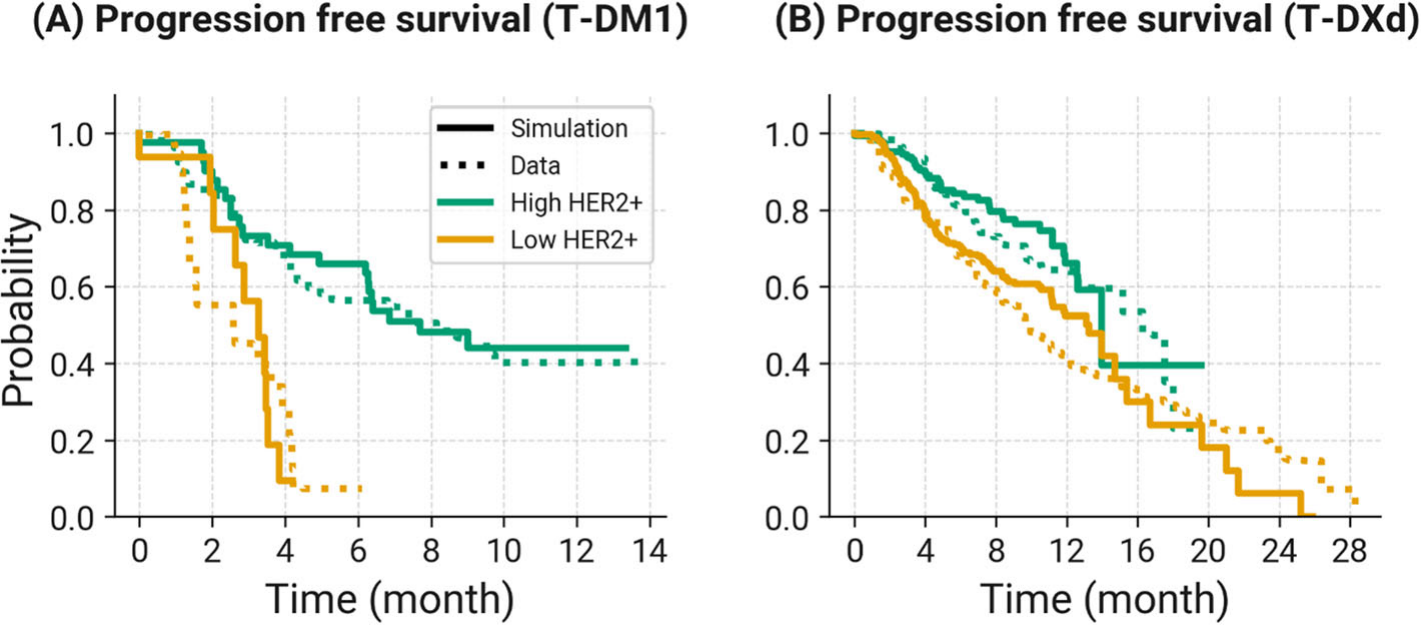
Comparison of clinical trial simulations of progression free survival (PFS) with observed clinical trial data for T-DM1 and T-DXd. **A** T-DM1 PFS simulations (solid lines) and observed data (dotted lines) from a phase 2 clinical trial [39] following administration of T-DM1 to HER2+ metastatic breast cancer (MBC) patients at 3.6 mg/kg Q3W for 14 months. **B** T-DXd PFS simulations (solid lines) and observed data (dotted lines) from a phase 2 [40] and a phase 3 clinical trial [41] following administration of T-DXd at 5.4 mg/kg Q3W to HER2+ MBC patients for 29 months. For both T-DM1 and T-DXd, clinical simulations and data were stratified based upon HER2 high (1e6/cell) and HER2 low (2e4) expression levels in MBC patients

T-DM1 simulations were performed at 3.6 mg/kg IV Q3W to match the phase 2 clinical trial data [39]. The simulations were used to calculate the probability of progression free survival out to 6–14 months for patients with high or low HER2 expression levels (Fig. 7A). For T-DXd, patients were simulated with 5.4 mg/kg IV Q3W dosing as in the phase 2 clinical trial data [40] and phase 3 data [41] (Fig. 7B). The model predictions of PFS were in good agreement with observed clinical trial data for T-DM1 and T-DXd. The model predicted greater PFS for T-DXd and could discriminate between patients with high and low HER2 expression for both ADCs, consistent with the data. Predictions were sensitive to tumor growth and death rates, and level of censorship in the studies.

## Discussion

### Encouraging clinical data, but also many clinical failures. How do we realize the clinical success of ADCs?

ADCs are among the fastest growing drug classes in oncology. They have demonstrated impressive clinical efficacy against treatment refractory cancers, with approvals in numerous and diverse indications [45]. However, many ADCs continue to fail in the clinic due to either lack of efficacy/ improvement over standard of care, or failure to reach efficacious doses due to dose limiting toxicities. Better methods are required to predict ADC efficacy and toxicity from preclinical data to optimize successful design and development of ADCs. There is also a need to learn from the previous generation of ADCs, in particular high level clinical failures, to understand the barriers that need to be overcome to enable clinical success and broader clinical utilization of ADCs.

The efficacy of an ADC depends on the intricacies of how the antibody, linker, and payload components interact with the tumor, which has important clinical implications. This requires a more quantitative understanding of ADC processing and activity that occurs prior to and following antibody-receptor engagement both on a cell-specific and a tumor-specific basis. Such knowledge would enable tuning of ADC antibody, linker and payload properties specifically for the selected target and tumor properties, informing better ADC design and selection. This would also facilitate development of a unifying framework enabling translation from preclinical studies to the clinic and prediction of clinical dose and regimen. Selection of doses for pivotal phase 2 clinical trials for ADCs by dose escalation to the maximum tolerated dose in the clinic may be a risky strategy that puts patients at risk for toxicities. A better approach to predict clinical dose and regimen for different indications is required, which would enable allocation of ADCs to those patients who are most likely to benefit from them. This is a two-part problem- there needs to be a better understanding of ADC targeting to the tumor and resulting efficacy, along with prevention of ADC uptake and payload release in normal tissues which can drive toxicity. In this manuscript we sought to establish a quantitative platform for prediction of ADC efficacy in the clinic via translation from preclinical data to the clinic. A second piece of work is ongoing to establish models to predict ADC toxicity in the clinic, which will be required for prediction of therapeutic index.

### Why is QSP modeling a useful tool for this problem? Why do we need a platform QSP model for ADCs?

Mathematical modeling approaches can be a useful tool for understanding mechanisms of drug action, increasing productivity, and enhancing decision making [46]. Empirical PK/PD models have proven very useful in the preclinical and clinical development of ADCs to maximize information obtained from experimental data, while minimizing resource utilization [8, 24, 47]. However, they are limited in their ability to predict efficacy across different targets and to inform design parameters. To understand the complexity of ADCs more mechanistic approaches are required, such as quantitative systems pharmacology (QSP) models that describe the dynamic interaction between biological systems and drugs [48]. A key feature of these models is their explicit distinction between ‘drug’ and ‘system’ parameters. For ADCs, system parameters include receptor expression on tumor and normal cells, internalization or turnover rates, and tumor growth rates, which can be obtained from the literature or prior experiments. Drug-specific parameters include PK parameters such as half-life, and pharmacological parameters such as target affinity and intrinsic efficacy of ADCs. As such QSP models contain sufficient mechanistic details to enable understanding of the processes critical to an ADC’s performance and to perform multiscale predictions.

Several QSP models have been developed for ADCs and are summarized by Lam et al*.* [7]. These models describe cellular mechanisms, tumor penetration, preclinical to clinical translation and clinical simulations. For example, a seminal paper by Shah and coworkers presented a bench to bedside translation of brentuximab vedotin using a multiscale mechanistic PK/PD model [49]. This model characterized the ADC and payload PK at the cellular level and in vivo in mouse plasma. It introduced a novel tumor disposition model to predict tumor payload concentrations of ADC and payload. The model also contained a tumor growth inhibition model which was used to characterize mouse xenograft data, and then translated to human. The model successfully predicted PFS rates in the clinic for brentuximab vedotin. A similar model structure and translational strategy was also applied by others for successful prediction of clinical outcome for T-DM1 [34, 50] and inotuzumab ozogamicin [51]. The latter paper was used to recommend a modified, fractionated regimen for a new indication of inotuzumab (ALL) which was successfully employed in the clinic. Vasalou and coworkers developed a mechanistic model of the solid tumor in a mouse model, incorporating essential mechanisms involved in ADC tumor localization and distribution [6]. They focused on intrinsic properties of the tumor, and controllable design parameters. This was a pivotal paper showing the impact of ADC design on tumor penetration, and quantitative solutions to the binding site barrier effect. This analysis was further explored by others [52, 53], including Khera et al*.* [54] who developed a model to study payload distribution as a function of antibody dose, payload dose, and payload properties. This work highlighted the importance of increasing ADC tissue penetration irrespective of bystander effects. Interestingly there are fewer publications on QSP models for ADC toxicity [55, 56], which is a definite gap in the science.

Although much progress has been made, there was a need for a more holistic platform model suitable for ADCs with varying mechanisms of action, that could be used for preclinical to clinical translation for ADCs with different tumor targets and classes of linkers and payloads. Platform models are QSP models which provide a common integrated quantitative knowledge repository for continued preclinical and clinical evaluation [57]. They are not specific to a particular drug and therefore can be re-applied, providing a mechanistic framework for predicting efficacy distinct from other pharmacometrics strategies.

### How did we approach the development of a platform QSP model for ADC efficacy?

This manuscript describes our initial steps towards a platform model for ADCs, integrating multiple data types and established knowledge to predict ADC efficacy (Fig. 1). Platform model development focused initially on the use of T-DM1 and T-DXd as test ADCs. These ADCs were selected as (1) there was sufficient data in the public domain for model calibration/ validation and (2) despite binding to the same target (HER2), they have disparate intracellular mechanisms of action which enable testing of different components of the model. Model development was approached in a modular and multi-scale fashion (Fig. 2), with verification of model predictions applied at each stage of the process by comparison to representative datasets. Although the model was trained on data for HER-2 ADCs, the framework is relevant for other ADCs simply by substitution of relevant parameters.

### Describing the intracellular mechanism of action of ADCs

First, a cellular model was developed to characterize ADC disposition and release of payload in the target tumor cells (Figure S1A). This model was used to describe in vitro incubations and was the basis of the tumor compartment in the in vivo models. The goal was to describe greater detail at the intracellular level compared to previous models, in order to sufficiently describe ADCs with different mechanisms of payload release and disposition. The model includes extracellular ADC deconjugation and competition of ADC with antibody for binding to the target cell, which is not accounted for in previous models. Upon binding to HER2, the ADC is internalized, and both the free and bound receptor can be recycled back to the cell surface. This was especially important for HER2-ADCs as HER2 exhibits a high recycling fraction [58], and recycling may occur before payload can be released inside the cell. Following internalization into cancer cells, T-DM1 traffics from the endosome to the lysosome where it undergoes proteolytic degradation, which releases the cytotoxic DM1-linker-lysine-metabolite (lysine-MCC-DM1) [59, 60]. In contrast, upon internalization of T-DXd, cleavage of the protease linker results in release of the DXd payload in the endosomes [61]. In the model, these different mechanisms are described by a degradation rate constant for T-DM1 describing release of payload along with the degradation of the antibody. For T-DXd an additional rate is included to describe linker-cleavage mediated release of the payload in the endosomes. Released payload can be transported to the cytosol where it is free to bind to its intracellular target (tubulin for lys-MCC-DM1 and TOPO-1 for DXd). The released payload species can also leave and re-enter the tumor cell, which is a function of its physicochemical properties and affinity for transport proteins.

The parameters for the cellular model were extracted from the literature or determined by fitting to data (Table S2). These included system properties such as HER2 expression across tumor cell lines, rates of internalization and recycling, and levels of tubulin or TOPO-1 inside cells. It also included drug specific properties including deconjugation rates and DAR for T-DM1 and T-DXd, trastuzumab binding affinity to HER2, DM1 affinity to tubulin and DXd inhibition of TOPO-1, and cellular efflux/influx rates of payload. Intracellular and extracellular payload diffusion rates were taken from Khera et al*.* [54] who performed a computational analysis to estimate these values based on parallel artificial membrane assay (PAMPA) data and physicochemical properties. The intracellular payload parameters can be the most difficult to obtain for ADC models, but since intracellular payload concentrations of payload drive efficacy (and toxicity) they are of fundamental importance [62, 63]. More reports of these studies are appearing in the literature, but further experiments are needed [54, 64]. The model was validated by comparison of model simulations to experimental results from diverse publications [14–16], following administration of trastuzumab and T-DM1 (Fig. 3 and S1B). In vitro cellular data was not available for T-DXd; however the in vitro model has been used to simulate T-DXd disposition and to make comparisons with T-DM1 [65].

### Implementation of a mechanistic tumor uptake model to predict intracellular payload concentrations

For accurate prediction of extent and time course of uptake of drug species into solid tumors, a mechanistic tumor distribution model was included. The model describes the exchange of ADC, Ab, and payload between the systemic circulation and tumor extracellular space using permeability and diffusion terms determined by their respective molecular size. The size of the tumor determines the extent of the distribution via either surface area or vascular exchange, representing avascular micro metastases and larger vascularized tumors, respectively. Previous publications [34, 49–51] have shown this approach can predict tumor ADC and payload concentration in mouse studies [66] and is clinically translatable. An important feature of the model is the dynamic interaction between the tumor disposition parameters and tumor size, where changes in tumor volume are directly able to influence the concentration of payload in the tumor, which is in turn responsible for the size of the tumor. To predict tumor payload concentrations in xenograft-bearing mice, the plasma PK model was linked to the tumor uptake model. Once in the tumor extracellular environment, cellular disposition of ADC, Ab and payload was then characterized using the parameters defined from the in vitro cellular model. For T-DM1 we show that the model is capable of predicting total tumor DM1 concentrations and intracellular concentrations of unconjugated DM1 in mice (Fig. 4). To accurately predict tumor DM1 concentration, it was necessary to use an intracellular tubulin concentration of 65 nM, which was the concentration estimated from previous modeling analyses [34, 49, 50]. This value is lower than experimental estimates of tubulin concentration from literature (range of 10–20 µM [67]) which were too high to describe the observed data. The current implementation of the model does not incorporate spatial distribution across the tumor tissue, but since it is based upon a general Krogh cylinder structure, it could be expanded to include this, which may be particularly useful for optimizing the design of ADCs.

### Tumor growth inhibition model

To describe the reduction in tumor volume in the mouse following administration of T-DM1 and T-DXd to xenograft models, an established TGI model was applied [24]. This model was selected as it includes a comprehensive tumor growth model describing exponential, linear and logistic growth, combined with a transit compartment tumor cell killing model, capable of describing different tumor growth and killing characteristics observed in vitro, in vivo, and in the clinic. Tumor killing was described as a function of intracellular unconjugated tumor payload concentration, predicted by the physiological PK model described above. As such the model does not specifically delineate between direct (targeted) tumor killing and bystander killing. Bystander killing refers to the ability of payloads to diffuse through membranes out of directly targeted cells into neighboring non-targeted cells resulting in cell death [68]. This mechanism of action has been postulated to be important for the treatment of tumors with heterogeneous expression of ADC targets, and thus may be an important design consideration. Bystander effect is observed for ADCs with cleavable linkers, like T-DXd, where smaller and more lipophilic payloads are released (e.g., DXd) that can diffuse across membranes [69]. ADCs with non-cleavable linkers, such as T-DM1, release larger/more hydrophilic amino acid-linker-payload metabolites (e.g., lysine-MCC-DM1) which do not readily diffuse across membranes to kill bystander cells. The impact of bystander killing on efficacy is not fully understood and has been found to be less efficient than direct killing [54]. Nonetheless, an addition to the model currently under testing includes an untargeted cell population, capable of exploring bystander effect.

The current model was able to describe the tumor growth and tumor cell killing observed following administration of different doses and regimens of T-DM1 and T-DXd to a range of mouse xenograft models (Fig. 4). The simulations predicted higher concentrations of tumor payload for T-DXd compared to T-DM1 for a given dose in the same tumor model, which was associated with greater efficacy of T-DXd. This may be due to the higher DAR of T-DXd compared to T-DM1, or more efficient release of its payload inside the cell resulting from its cleavable linker.

### Expanded human PK model accounting for multiple sinks

To describe the human PK, a comprehensive model was developed for a mechanistic description of ADC, Ab, and payload disposition in the systemic circulation and normal tissues including binding to various receptor sinks. Many ADCs bind to targets which have some level of expression on healthy cells in addition to tumor cells, and some of these receptors can also be shed from the membrane. For example, HER2 has been shown to be expressed on circulating NK cells, cardiomyocytes, skeletal muscle cells, and colonic epithelial cells [35]. The extracellular domain of HER2 is also shed from the cell surface, and serum concentrations of HER2 have been shown to be higher in metastatic breast cancer patients compared to healthy females [70]. Both the healthy cells expressing tumor target and the soluble target can act as a sink for ADC (or released antibody), reducing the exposure to the tumor cells for a given ADC dose. For HER2 this was demonstrated for trastuzumab, where high levels of serum extracellular domain are associated with rapid clearance and decreased benefit from trastuzumab therapy [70–72]. In addition, binding to these cells can result in on-target/ off-tumor toxicities [73] which can limit the dosing of ADCs in the clinic. As such, this is an important feature of a platform model. The model also describes deconjugation of ADC to release payload, and catabolism of ADC which can also release payload. Another distinguishing feature of the model is that it contains physiological volumes for central and peripheral compartments for ADC, Ab and payload, and as such moves away from more empirical PK descriptions. The model was able to predict the PK of T-DM1 and T-DXd in the clinic, including the non-linear PK of ADCs observed across the dose range explored in Phase 1a studies, without estimation of any parameters (Fig. 5). It was also capable of describing total antibody and payload concentrations observed at the efficacious doses of T-DM1 and T-DXd (Fig. 5).

### Predictions of tumor volume reduction and PFS in HER2+ MBC patients

The next step in the process was to combine the human PK model with the TGI model, to predict T-DM1 and T-DXd efficacy in HER2+ MBC patients. The overall structure of the mouse TGI model was kept the same, but the mouse system parameters such as initial tumor size, tumor growth rate, and receptor expression were replaced with clinically relevant parameters for HER2+ MBC. The drug effect parameters, including the maximum kill rate and the potency values were kept the same as those estimated from mouse TGI studies for T-DM1 and T-DXd. Initially the model was used to perform simulations for a nominal patient receiving T-DM1 and T-DXd given the same doses and regimen. The parameters relating to the different payloads of the two HER2 ADCs were different including DAR, deconjugation rate, cleavage rate in the endo/lysosomal compartment and intracellular target binding. In addition, the drug effect parameters including kill rate and potency were different. However, all other parameters in the model were the same. “The simulations (Fig. 6) show that for the same dose, tumor payload concentrations are much higher for T-DXd administration compared to T-DM1, which could be a factor that contributes to higher predicted efficacy for T-DXd, consistent with clinical observations. This points to intracellular tumor payload concentration, a function of both target mediated and target-independent uptake along with tumor penetration and intrinsic killing activity of the payload as factors that could be used to optimize drug design.

To identify the most sensitive parameters impacting tumor volume predictions, and to guide the selection of parameters for virtual clinical trial simulations, a global sensitivity analysis was completed (Figure S6). Parameters controlling tumor growth and death were the most sensitive parameters in the model. In particular, tumor growth rate has been shown to be a sensitive parameter from previous model analyses for ADCs [51]. As such, exponential and linear growth rates were varied in the clinical simulations, along with the pharmacodynamic killing parameters (*k*_*kill*_ and *kc*_*50*_). The model was used to perform virtual clinical trial simulations for T-DM1 and T-DXd, emulating conditions in pivotal phase 2 and phase 3 trials [39–41]. The model was able to predict PFS for T-DM1 and T-DXd that agreed with observed clinical data (Fig. 7), providing validation of model prediction of clinical outcome. In addition, the model predicted a difference in PFS for patients with low HER2 expression (20,000 receptors/cell, approximately HER2 1+) compared to patients with high HER2 expression (1 million receptors/cell, approximately HER2 3+). This was especially noticeable for T-DM1, which was predicted to have substantially shorter PFS in patients with low HER2 expression status compared to patients with high HER2 expression, as observed in clinical trial data [39–41]. In contrast, T-DXd was predicted to have longer PFS in patients with both high and low HER2 expression status. This suggests that the model could be used for head-to-head in silico predictions of clinical efficacy for different ADCs, which could be useful for early-stage assessments of efficacy differentiation and to provide rationale for pursuing clinical development. The model could also be used to investigate different clinical diagnostics of efficacy such as receptor expression, supporting precision medicine strategies for ADCs in the clinic.

### Model application and future developments

Significant progress has been made in the development of this next generation multiscale QSP model for ADCs, capable of supporting project decisions from exploratory research through to late-stage clinical trials. This platform model can be applied to other ADCs with different payloads and targeting different receptors, using data routinely collected for ADCs in the discovery process, or available from the literature (Table 1 and Table S4). For example, key target data to collect would include receptor expression, internalization rates and affinities. For specific payloads, important data would include affinity to intracellular target (or potencies) and permeability data, such as PAMPA. To investigate optimal ADC design at early stages, the model could be used to predict and optimize intracellular payload concentration as a surrogate for ADC efficacy, with comparison to competitor ADCs with similar payload class and clinical results. The model sensitivity to the concentration of payload in the tumor at half maximum kill rate suggests that maximizing the concentration of payload delivered to the tumor will enable successful treatment of more patients (assuming lack of toxicity). It is important to note that because this model does not explicitly capture tissue penetration, it is most useful in comparing molecules with similar biophysical properties. Likewise, the model captures killing on target-positive cells; thus, any bystander effect of target-negative cells are not considered, and the model might underestimate therapeutic efficacy. Once a lead ADC has been selected, the model can be linked to tumor cell killing, by fitting data from mouse TGI experiments, and then translated to the clinic. Strategies for answering specific drug development questions can be quite modular as the modeling can include or exclude various data complementary to the question poised. The goal is that the ADC model can provide a translational framework integrating data to provide a clearer understanding of the system, to help with design and to determine overall risk, so that ADCs could be progressed with a higher probability of success.

**Table 1:** Use-cases for the ADC platform model

Question 1	How do I optimize ADC design at early stages?		
Models to use	Cellular PK + Human PK		
Output	Predicted intracellular tumor payload concentration and/or binding to intracellular target for antibody target positive cells		
Data required	Parameters to measure experimentally or find in literature	Optional Parameters to measure or find in literature^a^	Parameters to assume and scan over with simulations
	• Receptor expression (tumor and healthy cells) • Internalization and degradation rates • Soluble target expression and turnover rates • Antibody binding affinity for membrane target (e.g., SPR) • Payload membrane permeability e.g., PAMPA	• Plasma stability • Lysosomal stability • Payload binding affinity for intracellular target • Intracellular target concentration	• Assume typical ADC first order t_1/2_ (e.g., 10 days) • Assign an ADC affinity to membrane target (e.g., 1 nM) • Assign a DAR (e.g., 4) • Assume tissue penetration is equivalent across formats
Example strategies	1. Calibrate to in vitro cellular disposition data of molecules with different DAR and affinities 2. Simulate tumor payload concentrations at different DARs, affinities, PK t_1/2_ s etc 3. Optimize tumor payload concentrations with comparison to competitor ADCs with similar payload class and clinical results 4. Optimize binding to intracellular target, consistent with MOA of payload		

Question 2	How do I translate from preclinical to clinical to predict clinical efficacious dose?	
Models to use	Cellular PK, Mouse PK + TGI, Human PK + TGI	
Output	Prediction of clinical PK (including TMDD) and clinical tumor volume reduction	
Data required	All data from Question 1	
	Preclinical data: • ADC/antibody/payload disposition data (if available) • Mouse PK • Mouse TGI vs different cell lines/PDX with different properties	Clinical data: • ADC/antibody/payload disposition data (if available) • Tumor doubling times
Example strategy	1. Develop tumor cellular model and calibrate to in vitro cellular disposition data (if available) 2. Develop mouse PK model and mouse TGI model 3. Develop human PK model 4. Link tumor cellular model, human PK model and TGI model. Parameterize using PD parameters determined from mouse and human PK and systems parameters 5. Perform model simulations of tumor volume reductions at different doses and regimens 6. Complete a global sensitivity analysis to determine key parameters impacting tumor volume reduction 7. Perform virtual clinical trial simulations varying sensitive parameters across plausible ranges	

Models and data required to address two standard questions asked during ADC drug development. Potential strategies are included to provide further context for motivation of the modeling questions

^a^Plasma stability can be used to inform deconjugation rates, lysosomal stability can inform cleavage/degradation rates of ADC to release payload, and permeability can inform the rates of influx/efflux across cells. However, for common payloads parameter estimates are also available from this manuscript and from others (e.g., Khera et al. [54])

Work is currently underway to validate predictions for other ADCs with varying mechanisms of action, including those that have failed in the clinic, which will frame the next version of the efficacy model. The modular nature of the model makes it suitable for expansion depending on the specific questions asked. For example, recent evidence indicates that heterogeneous tumoral distribution of ADCs can play a large role in limiting their efficacy [54]. Therefore, to be most relevant for design-based questions, a spatial version of the Krogh cylinder model should be incorporated to ensure optimal tumor penetration of ADCs. Additionally, inclusion of a sub model to predict bystander mediated killing distinct from targeted cell killing would be beneficial. Other future enhancements of the model would be to include other mechanisms of killing associated with ADCs including antibody dependent cellular phagocytosis (ADCP) and antibody dependent cell cytotoxicity (ADCC) [74]. Additional data would be needed to support inclusion of these processes in the platform model, but this would allow the model to answer questions with more granularity. Finally, and perhaps most importantly, prediction of ADC efficacy is only one part of the therapeutic index equation, and linkage to a toxicity model to predict dose limiting toxicities resulting from ADC administration will reduce ADC attrition in the clinic.

## Conclusions

In conclusion, the presented model is a step toward a platform QSP model and strategy for ADCs, integrating multiple types of data and knowledge to predict ADC efficacy. Compared to previous work, the model incorporates greater mechanistic detail, particularly at the intracellular level, to account for different mechanisms of ADC processing and payload release*.* It accounts for the disposition of the ADC, antibody, and payload inside and outside of the tumor, including binding to off-tumor on-target sinks. Alongside the model, the methods for translating to the clinic and performing virtual clinical trial simulations have been reassessed. The model was able to successfully predict clinical outcome for T-DM1 and T-DXd, and is a promising quantitative tool to support design, selection, and optimization of clinical dosing strategies for ADCs.

## Supplementary Information

Below is the link to the electronic supplementary material.

Supplementary file1 (DOCX 103 kb)

Supplementary file2 (DOCX 1025 kb)
