# Supplementary material for: Towards a platform quantitative systems pharmacology (QSP) model for preclinical to clinical translation of antibody drug conjugates (ADCs)
Source: J Pharmacokinet Pharmacodyn. 2023 Oct 3;51(5):429–47. doi: 10.1007/s10928-023-09884-6 (PMC11576657; doi:10.1007/s10928-023-09884-6)
Supplement: Supplementary file 1 — Supplementary file1 (DOCX 103 kb) [file 10928_2023_9884_MOESM1_ESM.docx]

# **Supplemental Information** for “Towards a platform quantitative systems pharmacology (QSP) model for preclinical to clinical translation of antibody-drug conjugated (ADCs)”

Journal of Pharmacokinetics/Pharmacodynamics

Bruna Scheuher, Khem Raj Ghusinga, Kimiko McGirr, Maksymilian Nowak, Sheetal Panday, Joshua Apgar, Kalyanasundaram Subramanian and Alison Betts

Corresponding author: Alison Betts, Applied BioMath, 561 Virginia Road, Concord MA 01742
Email: [alisonbetts72@gmail.com](mailto:alisonbetts72@gmail.com)

## Modeling convention

We have used three models in the main text, namely, the *in vitro* cellular model, the mouse model, and the human model. Each of these models are described as chemical reaction networks that are converted to ordinary differential equations. By default, the states in these models are assumed to be *amounts* of the species in nanomoles. As the reactions depend on concentrations of species, corresponding rate laws are derived in terms of amounts. In particular, the second order reactions require scaling of the rate constant by volume of the reaction.

## States/species of models

In this section, we provide the list of states/species used in the models.

### Table S1a: State variables used in the cellular model

| **Notation** | **Description** |
| --- | --- |
| $ADC_{ext}$ | Extracellular ADC |
| $Ab_{ext}$ | Extracellular antibody |
| $PL_{ext}$ | Extracellular payload |
| $HER2$ | Cell surface HER2 |
| $HER2:ADC$ | Cell surface HER2:ADC complex |
| $HER2:Ab$ | Cell surface HER2:Ab complex |
| $HER2_{endo}$ | Endosomal HER2 |
| $HER2:ADC_{endo}$ | Endosomal HER2:ADC complex |
| $HER2:Ab_{endo}$ | Endosomal HER2:Ab complex |
| $PL_{endo}$ | Endosomal payload |
| $PL_{cyto}$ | Cytosolic payload |
| $T_{cyto}$ | Cytosolic target of payload |
| ${T:PL}_{cyto}$ | Cytosolic target payload complex |

### Mouse modelTable S1b: State variables used in the mouse model

| **Notation** | **Description** |
| --- | --- |
| $ADC_{central}$ | ADC in central compartment |
| $Ab_{central}$ | Antibody in central compartment |
| $PL_{central}$ | Payload in central compartment |
| $ADC_{peripheral}$ | ADC in peripheral compartment |
| $Ab_{peripheral}$ | Antibody in peripheral compartment |
| $PL_{peripheral}$ | Payload in peripheral compartment |
| $ADC_{ext,tumor}$ | Extracellular ADC in tumor compartment |
| $Ab_{ext,tumor}$ | Extracellular antibody in tumor compartment |
| $PL_{ext,tumor}$ | Extracellular payload in tumor compartment |
| $N\{s\}$ | Number of tumor cells in stage $s$  $s\in\{1, 2, 3, 4\}$ |
| $HER2_{N\{s\},tumor}$ | Cell surface HER2 in tumor cells in stage $s$ |
| $HER{2:ADC}_{N\{s\},tumor}$ | Cell surface HER2:ADC in tumor cells in stage $s$ |
| $HER{2:Ab}_{N\{s\},tumor}$ | Cell surface HER2:Ab in tumor cells in stage $s$ |
| $HER2_{endo,N\{s\},tumor}$ | Endosomal HER2 in tumor cells in stage $s$ |
| $HER{2:ADC}_{endo,N\{s\},tumor}$ | Endosomal HER2:ADC in tumor cells in stage $s$ |
| $HER{2:Ab}_{endo,N\{s\},tumor}$ | Endosomal HER2:Ab in tumor cells in stage $s$ |
| $PL_{endo,N\{s\},tumor}$ | Endosomal payload in tumor cells in stage $s$ |
| $PL_{cyto,N\{s\},tumor}$ | Cytosolic HER2:ADC in tumor cells in stage $s$ |
| $T_{cyto,N\{s\},tumor}$ | Cytosolic intracellular target in tumor cells in stage $s$ |
| $T:PL_{cytoendo,N\{s\},tumor}$ | Cytosolic intracellular target payload complex in tumor cells in stage $s$ |

### Human Table S1c: Additional state variables used in the human model

| **Notation** | **Description** |
| --- | --- |
| $HER2_{central}$ | Cell surface HER2 in central compartment |
| $HER{2:ADC}_{central}$ | Cell surface HER2:ADC in central compartment |
| $HER{2:Ab}_{central}$ | Cell surface HER2:Ab in central compartment |
| $HER2_{endo,central}$ | Endosomal HER2 in central compartment |
| $HER{2:ADC}_{endo,central}$ | Endosomal HER2:ADC in central compartment |
| $HER{2:Ab}_{endo,central}$ | Endosomal HER2:Ab in central compartment |
| $sHER2_{central}$ | Soluble HER2 in central compartment |
| $sHER{2:ADC}_{central}$ | Soluble HER2:ADC in central compartment |
| $sHER{2:Ab}_{central}$ | Soluble HER2:Ab in central compartment |
| $HER2_{peripheral}$ | Cell surface HER2 in peripheral compartment |
| $HER{2:ADC}_{peripheral}$ | Cell surface HER2:ADC in peripheral compartment |
| $HER{2:Ab}_{peripheral}$ | Cell surface HER2:Ab in peripheral compartment |
| $HER2_{endo,peripheral}$ | Endosomal HER2 in peripheral compartment |
| $HER{2:ADC}_{endo,peripheral}$ | Endosomal HER2:ADC in peripheral compartment |
| $HER{2:Ab}_{endo,peripheral}$ | Endosomal HER2:Ab in peripheral compartment |
| $sHER2_{peripheral}$ | Soluble HER2 in peripheral compartment |
| $sHER{2:ADC}_{peripheral}$ | Soluble HER2:ADC in peripheral compartment |
| $sHER{2:Ab}_{peripheral}$ | Soluble HER2:Ab in peripheral compartment |
| $sHER2_{tumor}$ | Soluble HER2 in tumor compartment |
| $sHER{2:ADC}_{tumor}$ | Soluble HER2:ADC in tumor compartment |
| $sHER{2:Ab}_{tumor}$ | Soluble HER2:Ab in tumor compartment |

## Model parameters

In this section, we tabulate the model parameters. In some cases, we infer the parameters in the model reactions from these parameters, which are separately tabulated.

### Table S2a: *in vitro* cellular model parameters for T-DM1

| **Notation** | **Unit** | **Description** | **Value (Lower CI, Upper CI)** | **Source / Notes** |
| --- | --- | --- | --- | --- |
| $k_{dec}$ | $1/s$ | Rate constant for deconjugation of ADC | 6.41E-7  (1.31E-7, 3.13E-6) | Fit to [[1]](https://paperpile.com/c/z7MHEw/CEZ3v), Figure 3 |
| $DAR$ | - | Drug to antibody ratio | 3.5 | [[2]](https://paperpile.com/c/z7MHEw/eEFec) |
| $k_{on,Ab}$ | $1/nM/s$ | Association rate constant for binding of ADC or Ab with HER2 | 1E-4 | Typical value, [[3]](https://paperpile.com/c/z7MHEw/e2xpO) |
| $K_{D,Ab}$ | $nM$ | Equilibrium binding constant for HER2:ADC complex or HER2:Ab complex | 0.314  (1.27E-1,7.72E-1) | Fit to [[4]](https://paperpile.com/c/z7MHEw/1MDjR), Supplementary Figure 1B |
| $k_{endo,HER2}$ | $1/s$ | Endocytosis rate constant for HER2 | 4.27E-5  (6.16E-6, 2.96E-4) | Fit to [[4]](https://paperpile.com/c/z7MHEw/1MDjR), Supplementary Figure 1B |
| $k_{rec,HER2}$ | $1/s$ | Recycling rate constant for HER2 | 2.4E-5  (2.00E-6, 2.85E-4) | Fit to [[4]](https://paperpile.com/c/z7MHEw/1MDjR), Supplementary Figure 1B |
| $k_{deg,HER2}$ | $1/s$ | Degradation rate constant for HER2 | 1.27E-4  (6.77E-5, 2.40E-4) | Fit to [[4]](https://paperpile.com/c/z7MHEw/1MDjR), consistent with [[5]](https://paperpile.com/c/z7MHEw/ohbt3) |
| $k_{endo,HER2:Ab}$ | $1/s$ | Endocytosis rate constant for HER2:ADC or HER2:Ab | 4.27E-5  (6.16E-6, 2.96E-4) | Fit to [[4]](https://paperpile.com/c/z7MHEw/1MDjR), Supplementary Figure 1B |
| $k_{rec,HER2:Ab}$ | $1/s$ | Recycling rate constant for HER2:ADC or HER2:Ab | 2.4E-5  (2.00E-6, 2.85E-4) | Fit to [[4]](https://paperpile.com/c/z7MHEw/1MDjR), Supplementary Figure 1B |
| $k_{deg,HER2:Ab}$ | $1/s$ | Degradation rate constant for HER2:ADC or HER2:Ab | 1.27E-4  (6.77E-5, 2.40E-4) | Fit to [[4]](https://paperpile.com/c/z7MHEw/1MDjR), consistent with [[5]](https://paperpile.com/c/z7MHEw/ohbt3), Supplementary Figure 1B |
| $k_{cleave}$ | $1/s$ | Cleavage rate constant for ADC in endosomes | 0 | Assumption, non-cleavable linker |
| $k_{on,PL}$ | $1/nM/s$ | Association rate constant for payload binding to target | 1E-3 | Typical value, [[6, 7]](https://paperpile.com/c/z7MHEw/r6ObW+R60iQ) |
| $K_{D,PL}$ | $nM$ | Equilibrium binding constant for target bound payload | 930 | [[8]](https://paperpile.com/c/z7MHEw/C0Tqn) |
| $T_{per,cell}$ | $nM$ | Intracellular target concentration per cell | 65 | Estimated in [[9]](https://paperpile.com/c/z7MHEw/N90n4) |
| $k_{in,PL}$ | $1/s$ | Influx rate constant of payload | 5.95E-5 | [[10]](https://paperpile.com/c/z7MHEw/BMYGf) |
| $k_{out,PL}$ | $1/s$ | Efflux rate constant of payload | 3.95E-5 | [[10]](https://paperpile.com/c/z7MHEw/BMYGf) |
| **Experiment specific T-DM1 cellular model parameters** | | | | |
| $V_{cell}$ | $L$ | Volume of a cell | 2E-12 (used in [[4]](https://paperpile.com/c/z7MHEw/1MDjR),[[11]](https://paperpile.com/c/z7MHEw/HYKeD)) | Typical, [[12]](https://paperpile.com/c/z7MHEw/g3zI9) |
|  |  |  | 3.82E-12 (SKBr3, [[1]](https://paperpile.com/c/z7MHEw/CEZ3v)) | Fit to [[1]](https://paperpile.com/c/z7MHEw/CEZ3v), Figure 3 |
|  |  |  | 3.96E-12 (BT474, [[1]](https://paperpile.com/c/z7MHEw/CEZ3v)) | Fit to [[1]](https://paperpile.com/c/z7MHEw/CEZ3v), Figure 3 |
|  |  |  | 3.65E-12 (MCF7, [[1]](https://paperpile.com/c/z7MHEw/CEZ3v)) | Fit to [[1]](https://paperpile.com/c/z7MHEw/CEZ3v), Figure 3 |
| $V_{media}$ | $L$ | Volume of media in an *in vitro* experiment | 1E-2 | [[1]](https://paperpile.com/c/z7MHEw/CEZ3v) |
|  |  |  | 2E-3 | [[4]](https://paperpile.com/c/z7MHEw/1MDjR), [[11]](https://paperpile.com/c/z7MHEw/HYKeD) |
| $N_{cell}$ | count | Number of cells in an *in vitro* experiment | 1E7 | [[1]](https://paperpile.com/c/z7MHEw/CEZ3v) |
|  |  |  | 1.2E6 | [[11]](https://paperpile.com/c/z7MHEw/HYKeD) |
|  |  |  | 6E5 | Fit to [[4]](https://paperpile.com/c/z7MHEw/1MDjR), Supplementary Figure 1B |
| $RPC_{HER2,tumor}$ | count /  cell | Receptor per cell expressed on a tumor cell | 1.6E6 (used in [[4]](https://paperpile.com/c/z7MHEw/1MDjR),[[11]](https://paperpile.com/c/z7MHEw/HYKeD)) | Fit to [[4]](https://paperpile.com/c/z7MHEw/1MDjR), Supplementary Figure 1B |
|  |  |  | 1.12E6 (SKBr3, [[1]](https://paperpile.com/c/z7MHEw/CEZ3v)) | Fit to [[1]](https://paperpile.com/c/z7MHEw/CEZ3v), Figure 3 |
|  |  |  | 4.7E5 (BT474, [[1]](https://paperpile.com/c/z7MHEw/CEZ3v)) | Fit to [[1]](https://paperpile.com/c/z7MHEw/CEZ3v), Figure 3 |
|  |  |  | 1.3E6 (MCF7, [[1]](https://paperpile.com/c/z7MHEw/CEZ3v)) | Fit to [[1]](https://paperpile.com/c/z7MHEw/CEZ3v), Figure 3 |
| **Inferred parameters and initial conditions** | | | | |
| $k_{synth, HER2,tumor}$ | $1/s$ | Zeroth order synthesis rate of cell surface HER2 per cell | $k_{synth,HER2,tumor}=RPC_{HER2,tumor}\frac{k_{endo,HER2} k_{deg,HER2}}{k_{deg,HER2}+k_{rec,HER2}}$ | |
| $RPC_{HER2,endo,tumor}$ | count /  cell | Pre-drug endosomal HER2 per cell | $RPC_{HER2,endo,tumor}=\frac{k_{endo,HER2}}{k_{rec,HER2}+k_{deg,HER2}}RPC_{HER2,tumor}$ | |
| $k_{off,X}$ | $1/s$ | Dissociation rate constant for binding. $X=Ab$ for ADC or antibody; $X=PL$ for payload | $k_{off,X}=k_{on,X} K_{D,X}$ | |

### Table S2b: Mouse PK parameter values

The following in vitro cellular parameters were carried over to the mouse model:

$k_{endo,HER2},$ $k_{endo, HER2:Ab},$ $k_{rec,HER2}$, $k_{rec, HER2:Ab,}k_{deg,HER2,}$and $k_{deg,HER2:Ab}$

| **Parameter** | **Unit** | **Description** | **Value** | **Source** |
| --- | --- | --- | --- | --- |
| $V_{central}$ | $L$ | Volume of the central compartment | 1E-3 | [[13]](https://paperpile.com/c/z7MHEw/VG6iu) |
| $V_{peripheral}$ | $L$ | Volume of the peripheral compartment | 5.7E-3 | [[13]](https://paperpile.com/c/z7MHEw/VG6iu) |
| $V_{cell}$ | $L$ | Volume of a cell | 2E-12 | Typical, [[12]](https://paperpile.com/c/z7MHEw/g3zI9) |
| $RPC_{HER2,tumor}$ | $-$ | Receptors per cell expressed on a tumor cell | 1E6 | [[14]](https://paperpile.com/c/z7MHEw/Ico67) |
| $BW$ | $kg$ | Body weight | 2E-2 | [[15]](https://paperpile.com/c/z7MHEw/HWeXO) |
| ${MW}_{ADC}$ | $g/mol$ | Molecular weight of the ADC | 148781 (T-DM1) | [[2]](https://paperpile.com/c/z7MHEw/eEFec) |
|  |  |  | 153702  (T-DXd) | [[16]](https://paperpile.com/c/z7MHEw/M3pTK) |
| ${MW}_{Ab}$ | $g/mol$ | Molecular weight of the antibody | 145167 | [[17]](https://paperpile.com/c/z7MHEw/UqZ1b) |
| ${MW}_{PL}$ | $g/mol$ | Molecular weight of the payload | 1100 | Lys-MCC-DM1 [[2]](https://paperpile.com/c/z7MHEw/eEFec) |
|  |  |  | 493 | DXd; Calculated from structure [[18]](https://paperpile.com/c/z7MHEw/nxb5b) |
| $t_{½, Ab}$ | $day$ | Elimination half-life of antibody/ADC | 11.6 | Fit to non-tumor bearing mouse PK [[1]](https://paperpile.com/c/z7MHEw/CEZ3v) |
| $t_{½, PL}$ | $hour$ | Elimination half-life of payload | 3 (T-DM1) | Fit to non-tumor bearing mouse PK of a similar molecule [[19]](https://paperpile.com/c/z7MHEw/C9GdY) |
|  |  |  | 0.85 (T-DXd) | Fit to non-tumor bearing mouse PK [[20]](https://paperpile.com/c/z7MHEw/KnoSQ) |
| $t_{dist12, Ab}$ | $hour$ | Time of distribution of antibody/ADC from central to peripheral compartment | 2.65 | Fit to to non-tumor bearing mouse PK [[1]](https://paperpile.com/c/z7MHEw/CEZ3v) |
| $P_{dist12, Ab}$ | $-$ | Partition coefficient of antibody/ADC between central and peripheral compartments | 0.34 | Fit to to non-tumor bearing mouse PK [[1]](https://paperpile.com/c/z7MHEw/CEZ3v) |
| $t_{dist12, PL}$ | $hour$ | Time of distribution of payload from central to peripheral compartment | 0.1 | Fit to to non-tumor bearing mouse PK [[1]](https://paperpile.com/c/z7MHEw/CEZ3v) |
| $P_{dist12, PL}$ | $-$ | Partition coefficient of payload between central and peripheral compartments | 83.02 | Fit to non-tumor bearing mouse PK [[1]](https://paperpile.com/c/z7MHEw/CEZ3v) |
| $P_{Ab}$ | $\mu m/day$ | Vascular permeability of antibody/ADC | 50.34 | Fit to BT474-EEI mouse PK [[1]](https://paperpile.com/c/z7MHEw/CEZ3v) |
| $D_{Ab}$ | $\mu m^{2}/day$ | Diffusivity of antibody/ADC | 1.74E5 | Fit to BT474-EEI mouse PK [[1]](https://paperpile.com/c/z7MHEw/CEZ3v) |
| $P_{PL}$ | $\mu m/day$ | Vascular permeability of payload | 924 | Scaled by $\varepsilon_{PL}$ from [[21]](https://paperpile.com/c/z7MHEw/SFIj9) |
| $D_{PL}$ | $\mu m^{2}/day$ | Diffusivity of payload | 1.1E7 | Scaled by $\varepsilon_{PL}$ from [[21]](https://paperpile.com/c/z7MHEw/SFIj9) |
| $R_{cap}$ | $\mu m$ | Capillary radius | 8 | [[10]](https://paperpile.com/c/z7MHEw/BMYGf) |
| $R_{Krogh}$ | $\mu m$ | Krogh cylinder radius | 75 | [[10]](https://paperpile.com/c/z7MHEw/BMYGf) |
| $\varepsilon_{Ab}$ | $-$ | Acellular fraction of tumor volume available to antibody/ADC | 0.24 | [[10]](https://paperpile.com/c/z7MHEw/BMYGf) |
| $\varepsilon_{PL}$ | $-$ | Acellular fraction of tumor volume available to payload | 0.44 | [[10]](https://paperpile.com/c/z7MHEw/BMYGf) |
| $k_{dec}$ | $1/s$ | Rate constant for deconjugation of ADC | 8.5E-7 (T-DM1) | Fit to non tumor-bearing mouse PK [[1]](https://paperpile.com/c/z7MHEw/CEZ3v) |
|  |  |  | 1.7E-7 (T-DXd) | Fit to non tumor-bearing mouse PK [[20]](https://paperpile.com/c/z7MHEw/KnoSQ) |
| $DAR$ | $-$ | Drug to antibody ratio | 3.5 (T-DM1) | [[2]](https://paperpile.com/c/z7MHEw/eEFec) |
|  |  |  | 8 (T-DXd) | [[18]](https://paperpile.com/c/z7MHEw/nxb5b) |
| $k_{on,Ab}$ | $1/nM/s$ | Association rate constant for binding of HER2 with ADC or Ab | 1E-4 | [[3]](https://paperpile.com/c/z7MHEw/e2xpO) |
| $K_{D,Ab}$ | $nM$ | Equilibrium binding constant of HER2:ADC or HER2:Ab complex | 0.3 | Carried forward from *in vitro* fitting |
| $k_{on,PL}$ | $1/nM/s$ | Association rate constant for payload binding to target | 1E-3 | Typical value, [[6, 7]](https://paperpile.com/c/z7MHEw/r6ObW+R60iQ) |
| $K_{D, PL}$ | $nM$ | Equilibrium binding constant of target bound payload | 930 (DM1) | [[8]](https://paperpile.com/c/z7MHEw/C0Tqn) |
|  |  |  | 307 (DXd)^1^ | Calculated K_i_ from [[22, 23]](https://paperpile.com/c/z7MHEw/JLHX+pSCU5) |
| $T_{per,cell}$ | $nM$ | Concentration of target in the cell | 65 (Tubulin) | Estimated in [[9]](https://paperpile.com/c/z7MHEw/N90n4) |
|  |  |  | 297 (TOPO-1)^2^ | Estimated from [[24]](https://paperpile.com/c/z7MHEw/usCM) |
| $k_{in,PL}$ | $1/s$ | Influx rate constant for payload | 5.95E-5 (T-DM1) | [[10]](https://paperpile.com/c/z7MHEw/BMYGf) |
|  |  |  | 0.0128 (T-DXd) | [[10]](https://paperpile.com/c/z7MHEw/BMYGf) |
| $k_{out,PL}$ | $1/s$ | Efflux rate constant for payload | 3.94E-5 (T-DM1) | [[10]](https://paperpile.com/c/z7MHEw/BMYGf) |
|  |  |  | 0.00898 (T-DXd) | [[10]](https://paperpile.com/c/z7MHEw/BMYGf) |
| ${Pc}_{PL}$ | $-$ | Partition coefficient of payload in tumor tissue | 0.51 (T-DM1) | [[10]](https://paperpile.com/c/z7MHEw/BMYGf) |
|  |  |  | 0.43 (T-DXd) | [[10]](https://paperpile.com/c/z7MHEw/BMYGf) |
| **Inferred parameters (in addition to those described in *in vitro* parameter table)** | | | | |
| $k_{12, X}$ | $1/s$ | Central to peripheral transport rate constant. $X=Ab$ for ADC or antibody; $X=PL$ for payload. | $\frac{log2}{t_{dist, X}}\frac{Pdist_{12, X}}{Pdist_{12, X}+V_{central}/V_{peripheral}}$ | |
| $k_{21, X}$ | $1/s$ | Peripheral to central transport rate constant. $X=Ab$ for ADC or antibody; $X=PL$ for payload. | $\frac{log2}{t_{dist, X}}\frac{V_{central}/V_{peripheral}}{Pdist_{12, X}+V_{central}/V_{peripheral}}$ | |
| $k_{13, X}$ | $1/s$ | Central to peripheral transport rate constant. $X=Ab$ for ADC or antibody; $X=PL$ for payload. | $\left( \frac{2P_{X}R_{cap}}{{R^{2}}_{Krogh}}+\frac{6D_{X}R_{cap}}{(V_{tumor})^{2/3}} \right)\frac{V_{tumor}}{V_{central}}$ | |
| $k_{31, X}$ | $1/s$ | Peripheral to central transport rate constant. $X=Ab$ for ADC or antibody; $X=PL$ for payload. | $\left( \frac{2P_{X}R_{cap}}{{R^{2}}_{Krogh}}+\frac{6D_{X}R_{cap}}{(V_{tumor})^{2/3}} \right)\frac{1}{\varepsilon_{X}}$ | |

^1^Estimation of target burden for TOPO-1: Topoisomerase I was measured to be 0.9 - 9.8 ng/mL in 1 g protein extract[[24]](https://paperpile.com/c/z7MHEw/usCM). Considering the geometric mean of the measured Topoisomerase I concentration (2.97 ng/mL) in 100 L sample volume, we obtain 0.297 ng Topoisomerase I in 1 ug protein extract (assumed per 100 uL sample volume). Assuming 100 kDa molecular weight of Topoisomerase I and 200 pg/cell of protein mass in protein extract (range 100 - 300 pg/cell[[25]](https://paperpile.com/c/z7MHEw/lNHC)) gives 297 nM, where cellular volume is assumed to be 2E-12 L as in the parameter table.

^2^Estimation of K_D_ of DXd: K_D_ was assumed to be related to Ki. Ki was calculated by Ki = IC50/ (Km/[S] + 1); where Km is defined by the dissociation constant of Topoisomerase binding to DNA = 2.7 nM[[26]](https://paperpile.com/c/z7MHEw/oAtP) and the S concentration used was 290 nM in the Kawato et al. (1991) SN38 study (assumed to have the same relationship as DXd)[[22]](https://paperpile.com/c/z7MHEw/JLHX).

### Table S2c: Mouse PD parameter values

| **Parameter** | **Unit** | **Description** | **Value** | **Source** |
| --- | --- | --- | --- | --- |
| $\psi$ | $-$ | Switch between exponential and linear growth phases | 20 | [[27]](https://paperpile.com/c/z7MHEw/8iEMn) |
| $V_{tumor, max}$ | $L$ | Maximum tumor volume | 5E-3 | Assumption |
| **BT-474 Cells with T-DM1** | | | | |
| $k_{kill, max}$ | $1/day$ | Maximum killing rate | 0.49 | Fit to [[28]](https://paperpile.com/c/z7MHEw/qaMx4) |
| $\tau$ | $day$ | Time delay of killing | 3.54 | Fit to [[28]](https://paperpile.com/c/z7MHEw/qaMx4) |
| ${kc}_{50}$ | $nM$ | Half-maximal killing concentration | 100 | Fit to [[28]](https://paperpile.com/c/z7MHEw/qaMx4) |
| $n_{Hill}$ | $-$ | Hill coefficient of killing | 4 | Fit to [[28]](https://paperpile.com/c/z7MHEw/qaMx4) |
| $t_{double}$ | $day$ | Exponential tumor doubling time | 11.35 | Fit to [[28]](https://paperpile.com/c/z7MHEw/qaMx4) |
| $k_{lin}$ | $mm^{3}/day$ | Linear tumor growth rate | 73.08 | Fit to [[28]](https://paperpile.com/c/z7MHEw/qaMx4) |
| **BT474EEI Cells with T-DM1** | | | | |
| $k_{kill, max}$ | $1/day$ | Maximum killing rate | 0.24 | Fit to [[1]](https://paperpile.com/c/z7MHEw/CEZ3v) |
| $\tau$ | $day$ | Time delay of killing | 1E-3 | Tuned to ensure early killing as seen in data [[1]](https://paperpile.com/c/z7MHEw/CEZ3v) |
| ${kc}_{50}$ | $nM$ | Half-maximal killing concentration | 236.8 | Fit to [[1]](https://paperpile.com/c/z7MHEw/CEZ3v) |
| $n_{Hill}$ | $-$ | Hill coefficient of killing | 1 | Fit to [[1]](https://paperpile.com/c/z7MHEw/CEZ3v) |
| $t_{double}$ | $day$ | Exponential tumor doubling time | 41.78 | Fit to [[1]](https://paperpile.com/c/z7MHEw/CEZ3v) |
| $k_{lin}$ | $mm^{3}/day$ | Linear tumor growth rate | 90 | Estimated |
| **N87 Cells with T-DM1 and T-DXd** | | | | |
| $k_{kill, max}$ | $1/day$ | Maximum killing rate | 0.31 (T-DM1) | Fit to [[29]](https://paperpile.com/c/z7MHEw/02x49) |
|  |  |  | 0.24 (T-DXd) | Fit to [[23]](https://paperpile.com/c/z7MHEw/pSCU5) |
| $\tau$ | $day$ | Time delay of killing | 0.25 | Fit to [[29]](https://paperpile.com/c/z7MHEw/02x49) |
|  |  |  | 0.17 (T-DXd) | Tuned to ensure early killing as seen in data [[23]](https://paperpile.com/c/z7MHEw/pSCU5) |
| ${kc}_{50}$ | $nM$ | Half-maximal killing concentration | 485 (T-DM1) | Fit to [[29]](https://paperpile.com/c/z7MHEw/02x49) |
|  |  |  | 13.68 (T-DXd) | Fit to [[23]](https://paperpile.com/c/z7MHEw/pSCU5) |
| $n_{Hill}$ | $-$ | Hill coefficient of killing | 1 | Fit to [[29]](https://paperpile.com/c/z7MHEw/02x49) |
|  |  |  | 2 | Fit to [[23]](https://paperpile.com/c/z7MHEw/pSCU5) |
| $t_{double}$ | $day$ | Exponential tumor doubling time | 12.37 | Fit to [[29]](https://paperpile.com/c/z7MHEw/02x49) |
|  |  |  | 9.70 | Fit to [[23]](https://paperpile.com/c/z7MHEw/pSCU5) |
| $k_{lin}$ | $mm^{3}/day$ | Linear tumor growth rate | 189.56 | Fit to [[29]](https://paperpile.com/c/z7MHEw/02x49) |
|  |  |  | 175 | Fit to [[23]](https://paperpile.com/c/z7MHEw/pSCU5) |
| **KPL4 Cells with T-DM1** | | | | |
| $k_{kill, max}$ | $1/day$ | Maximum killing rate | 0.49 | Fit to [[30]](https://paperpile.com/c/z7MHEw/mzKcO) |
| $\tau$ | $day$ | Time delay of killing | 0.48 | Fit to [[30]](https://paperpile.com/c/z7MHEw/mzKcO) |
| ${kc}_{50}$ | $nM$ | Half-maximal killing concentration | 42.6 | Fit to [[30]](https://paperpile.com/c/z7MHEw/mzKcO) |
| $n_{Hill}$ | $-$ | Hill coefficient of killing | 1 | Fit to [[30]](https://paperpile.com/c/z7MHEw/mzKcO) |
| $t_{double}$ | $day$ | Exponential tumor doubling time | 6.16 | Fit to [[30]](https://paperpile.com/c/z7MHEw/mzKcO) |
| $k_{lin}$ | $mm^{3}/day$ | Linear tumor growth rate | 113.45 | Fit to [[30]](https://paperpile.com/c/z7MHEw/mzKcO) |
| **Inferred parameters and states (in addition to those previously described)** | | | | |
| $\boldsymbol{P}$ $\boldsymbol{L}_{\boldsymbol{total}\boldsymbol{,}\boldsymbol{N}\boldsymbol{1}}$ | **nmol** | **Total deconjugated, intracellular PL in N1** | $\boldsymbol{P}$ $\boldsymbol{L}_{\boldsymbol{total}}\boldsymbol{=}\boldsymbol{P}\boldsymbol{L}_{\boldsymbol{endo}\boldsymbol{,}\boldsymbol{N}\boldsymbol{1,}\boldsymbol{tumor}}\boldsymbol{+}\boldsymbol{P}\boldsymbol{L}_{\boldsymbol{cyto}\boldsymbol{,}\boldsymbol{N}\boldsymbol{1,}\boldsymbol{tumor}}$  $\boldsymbol{+}\boldsymbol{T}\boldsymbol{:}\boldsymbol{P}$ $\boldsymbol{L}_{\boldsymbol{cyto}\boldsymbol{,}\boldsymbol{N}\boldsymbol{1,}\boldsymbol{tumor}}$ | |
| $\boldsymbol{k}_{\boldsymbol{kill}}$ | 1/s | Tumor killing rate | $k_{kill}=\frac{P{L^{n_{kill}}}_{total, N1}}{1+P{L^{n_{kill}}}_{total, N1}}$ | |
| $V_{ext, tumor}$ | $L$ | Acellular/interstitial volume in tumor | $V_{ext,tumor}=\varepsilon_{PL}V_{tumor}$ | |
| $V_{int, tumor}$ | $L$ | Cellular/internal volume in tumor | $V_{int, tumor}=(1-\varepsilon_{PL})V_{tumor}$ | |
| $N_{cell, tumor}$ | - | Number of tumor cells | $N_{cell,tumor}=\frac{V_{int, tumor}}{V_{cell}}=\sum_{s} N_{\{s\}}$ | |
| $N_{max, tumor}$ | - | Number of tumor cells in maximum tumor volume | $N_{max, tumor}=\frac{{(1-\varepsilon_{PL})V}_{tumor, max}}{V_{cell}}$ | |
| $k_{growth}$ | $1/s$ | Specific growth rate of tumor cells | $k_{growth}=\frac{\frac{log2}{t_{double}}\left( 1-\frac{N_{cell,tumor}}{N_{max, tumor}} \right)}{\left( 1+\left( \frac{log2 N_{cell,tumor}V_{cell}}{t_{double}(1-\varepsilon_{PL})k_{lin}}\times10^{6} \right)^{\psi} \right)^{1/\psi}}$ | |
| $V_{ext,Ab, tumor}$ | $L$ | Acellular/interstitial volume accessible to ADC or antibody | $V_{ext, Ab, tumor}=\varepsilon_{Ab}V_{tumor}$ | |

### Table S2d: Human parameter values

We carried over all model parameters from the mouse model, except for the ones tabulated below. For tumor killing parameters, we used N87 values.

| **Parameter** | **Unit** | **Description** | **Value** | **Source** |
| --- | --- | --- | --- | --- |
| $V_{central}$ | $L$ | Volume of the central compartment | 3 | [[13]](https://paperpile.com/c/z7MHEw/VG6iu) |
| $V_{peripheral}$ | $L$ | Volume of the peripheral compartment | 13 | [[13]](https://paperpile.com/c/z7MHEw/VG6iu) |
| $V_{tumor, i}$ | $L$ | Initial tumor volume | 0.0016 | [[31]](https://paperpile.com/c/z7MHEw/vwMxy) |
| $V_{tumor, max}$ | $L$ | Maximum tumor volume | 0.5238 | [[32]](https://paperpile.com/c/z7MHEw/F29sM) |
| $V_{cell}$ | $L$ | Volume of a cell | 2.00E-12 | Typical, [[12]](https://paperpile.com/c/z7MHEw/g3zI9) |
| $RPC_{HER2, tumor}$ | count / cell | Receptors per cell expressed on a tumor cell | 1.00E+06 | [[14]](https://paperpile.com/c/z7MHEw/Ico67) |
| $RPC_{HER2, normal}$ | count / cell$-$ | Receptors per cell expressed on a normal cell | 2.00E+04 | [[14, 33]](https://paperpile.com/c/z7MHEw/aDFOh+Ico67) |
| $N_{cell, central}$ | count | Number of HER2+ cells in the central compartment | 2.74E+08 | Calibrated to human PK |
| $N_{cell, peripheral}$ | count | Number of HER2+ cells in the peripheral compartment | 2.08E+11 | Calibrated to human PK |
| $C_{sHER2, central}$ | $ng/mL$ | Concentration of sHER2 in central compartment in healthy individuals | 8 | [[34]](https://paperpile.com/c/z7MHEw/1sYsC) ^1^ |
| $\chi_{shed, HER2:Ab}$ | $-$ | Fraction of shedding that occurs when receptor is bound to antibody | 0 | Assumption based on [[35]](https://paperpile.com/c/z7MHEw/V2O9Y) |
| $BW$ | $kg$ | Body weight | 70 | [[13]](https://paperpile.com/c/z7MHEw/VG6iu) |
| ${MW}_{sHER2}$ | $g/mol$ | Molecular weight of soluble HER2 | 1.00E+05 | [[35]](https://paperpile.com/c/z7MHEw/V2O9Y) |
| $t_{½, sHER2}$ | $hour$ | Elimination half-life of sHER2 | 5 | Estimated based on [[36]](https://paperpile.com/c/z7MHEw/MAWK9), [[37]](https://paperpile.com/c/z7MHEw/vRwkD), and typical Ab half life |
| $t_{½, sHER2:Ab}$ | $day$ | Elimination half-life of sHER2:ADC complex or sHER2:Ab complex | 11.6 | Set to half-life of antibody/ADC [[1, 20]](https://paperpile.com/c/z7MHEw/CEZ3v+KnoSQ) |
| $t_{dist12, sHER2}$ | $hour$ | Time of distribution of sHER2 from central to peripheral compartment | 8 | Set to time-scale of permeability of Ab |
| $P_{dist12, sHER2}$ | $-$ | Partition coefficient of sHER2 between central and peripheral compartments | 1 | Assumption |
| $t_{dist13, sHER2}$ | $hour$ | Time of distribution of sHER2 from central to tumor compartment | 8 | Set to time-scale of permeability of Ab |
| $P_{dist13, sHER2}$ | $-$ | Partition coefficient of sHER2 between central and tumor compartments | 0.24 | Set to acellular fraction of tumor volume available to antibody/ADC [[10]](https://paperpile.com/c/z7MHEw/BMYGf) |
| $t_{dist12, Ab}$ | $hour$ | Time of distribution of antibody/ADC from central to peripheral compartment | 10 | Calibrated to human PK [[38, 39]](https://paperpile.com/c/z7MHEw/Rvug9+cCf18) |
| $P_{dist12, Ab}$ | $-$ | Partition coefficient of antibody/ADC between central and peripheral compartments | 0.187 | Typical, [[40]](https://paperpile.com/c/z7MHEw/BcIfV) |
| $t_{double}$ | $day$ | Exponential tumor doubling time | 25 | [[32]](https://paperpile.com/c/z7MHEw/F29sM) |
| $k_{lin}$ | $mm^{3}/day$ | Linear tumor growth rate | 621 | Estimated from [[41]](https://paperpile.com/c/z7MHEw/WCwlJ) |
| $k_{dec}$ | $1/s$ | Rate constant for deconjugation of ADC | 8.5E-7 (T-DM1) | Carried over from mouse model |
|  |  |  | 1.67E-8 (T-DXd) | [[23]](https://paperpile.com/c/z7MHEw/pSCU5) |
| **Inferred parameters (in addition to those previously described)** | | | | |
| $k_{synth, HER2,X}$ | $1/s$ | Synthesis rate of cell surface HER2. $X=normal$ for normal cells and $X=tumor$ for tumor cells. | $k_{synth, HER2,normal}=RPC_{HER2, X}\left( k_{shed}+\frac{k_{endo,HER2} k_{deg,HER2}}{k_{deg,HER2}+k_{rec,HER2}} \right)$ | |
| $k_{shed, HER2}$ | $1/s$ | HER2 shedding rate | $k_{shed,HER2}=\frac{C_{sHER2, central}}{RPC_{HER2,normal}}\frac{k_{deg,sol}\left( k_{deg,sol}+k_{12}+k_{21} \right)}{{\left( k_{deg,sol}+k_{21} \right)N}_{cell, central}+k_{21}N_{cell, peripheral}}$ | |
| **Inferred parameters (in addition to those described in *in vitro* parameter table)** | | | | |
| $k_{12, X}$^2^ | $1/s$ | Central to peripheral transport rate constant. | $\frac{log2}{t_{dist, X}}\frac{Pdist_{12, X}}{Pdist_{12, X}+V_{central}/V_{peripheral}}$ | |
| $k_{21, X}$^2^ | $1/s$ | Peripheral to central transport rate constant. | $\frac{log2}{t_{dist, X}}\frac{V_{central}/V_{peripheral}}{Pdist_{12, X}+V_{central}/V_{peripheral}}$ | |
| $k_{13, X}$^2^ | $1/s$ | Central to tumor transport rate constant. | $\frac{log2}{t_{dist, X}}\frac{Pdist_{12, X}}{Pdist_{13, X}+V_{central}/V_{ext, Ab, tumor}}$ | |
| $k_{31, X}$^2^ | $1/s$ | Peripheral to tumor transport rate constant. | $\frac{log2}{t_{dist, X}}\frac{V_{central}/V_{ext, Ab, tumor}}{Pdist_{13, X}+V_{central}/V_{ext, Ab, tumor}}$ | |

^1^sHER2 level in the blood of healthy individuals was used to calibrate shedding rate. Differences in sHER2 levels between healthy and diseased individuals are driven by shedding from the tumor and are consistent with available experimental data.

^2^$X=sHER2$ for sHER2; $X=sHER2:Ab$ for sHER2:Ab or sHER2:ADC complex;

### Table S2e: Virtual patient parameter values. All parameter distributions were sampled from a Normal distribution with corresponding coefficients of variation that were calibrated to match PFS clinical data.

| **Parameter** | **Unit** | **Mean** | **Coefficient of Variation** | **Source** |
| --- | --- | --- | --- | --- |
| $t_{double}$ | $day$ | 25 | 20% | [[32]](https://paperpile.com/c/z7MHEw/F29sM) |
| $k_{lin}$ | $mm^{3}/day$ | 621 | 20% | [[32]](https://paperpile.com/c/z7MHEw/F29sM) |
| **T-DM1** | | | | |
| $k_{kill, max}$ | $1/day$ | 1.39e-1 | 10% | Mean calibrated from mouse; CV calibrated to PFS |
| ${kc}_{50}$ | $nM$ | 23.8 | 2000% | Mean calibrated from mouse; CV calibrated to PFS |
| **T-DXd** | | | | |
| $k_{kill, max}$ | $1/day$ | 2.01e-1 | 10% | Mean calibrated from mouse; CV calibrated to PFS |
| ${kc}_{50}$ | $nM$ | 9.54 | 10000% | Mean calibrated from mouse; CV calibrated to PFS |

## Reactions and resulting ordinary differential equations

### Table S3a: In vitro cellular model reactions

| **Reaction** | **Rate law** |
| --- | --- |
| $ADC_{ext} \to Ab_{ext} + DAR * PL_{ext}$ | $v_{1}=k_{dec}ADC_{ext}$ |
| $\emptyset\to HER2$ | $v_{2}=k_{synth, no shedding}$ |
| $HER2+ADC_{ext}\leftrightarrow HER2:ADC$ | $v_{3f}=\frac{k_{on,Ab}}{V_{media}}HER2 \times ADC_{ext}$  $v_{3r}=k_{on,Ab}K_{D,Ab}HER2:ADC$ |
| $HER2+Ab_{ext}\leftrightarrow HER2:Ab$ | $v_{4f}=\frac{k_{on}}{V_{media}}HER2 \times Ab_{ext}$  $v_{4r}=k_{on,Ab}K_{D,Ab}HER2:Ab$ |
| $HER2\leftrightarrow HER2_{endo}$ | $v_{5f}=k_{endo, HER2} HER2$  $v_{5r}=k_{rec, HER2} HER2_{endo}$ |
| $HER2:ADC\leftrightarrow HER{2:ADC}_{endo}$ | $v_{6f}=k_{endo, HER2:Ab} HER2:ADC$  $v_{6r}=k_{rec, HER2:Ab} HER{2:ADC}_{endo}$ |
| $HER2:Ab\leftrightarrow HER{2:Ab}_{endo}$ | $v_{7f}=k_{endo, HER2:Ab} HER2:Ab$  $v_{7r}=k_{rec, HER2:Ab} HER{2:Ab}_{endo}$ |
| $HER2_{endo}\to\emptyset$ | $v_{8}=k_{deg,HER2} HER2_{endo}$ |
| $HER{2:ADC}_{endo}\to DAR*PL_{endo}$ | $v_{9}=k_{deg,HER2:Ab} HER{2:ADC}_{endo}$ |
| $HER{2:Ab}_{endo}\to\emptyset$ | $v_{10}=k_{deg,HER2:Ab} HER{2:Ab}_{endo}$ |
| $HER{2:ADC}_{endo}\to HER{2:Ab}_{endo}+DAR*PL_{endo}$ | $v_{11}=k_{cleave} HER{2:ADC}_{endo}$ |
| $PL_{endo}\to PL_{cyto}$ | $v_{12}=k_{in} PL_{endo}$ |
| $T_{cyto}+PL_{cyto}\leftrightarrow T:PL_{cyto}$* | $v_{13f}=\frac{k_{on,PL}}{{{}_{PL}V}_{cell}}T_{cyto}\times PL_{cyto}$  $v_{13r}=k_{on,PL}K_{D,PL}T:PL_{cyto}$ |
| $PL_{cyto}\leftrightarrow PL_{ext}$ | $v_{14f}=k_{out}PL_{cyto}$  $v_{14r}=k_{in}PL_{ext}$ |

*$P$ $c_{PL}$ = 1 in vitro.

### Table S3b: Resulting in vitro cellular model ODEs

| **State** | **ODE** | **Equation** |
| --- | --- | --- |
| $A{DC}_{ext}$ | $\frac{dADC_{ext}}{dt}=-v_{1}-v_{3f}+v_{3r}$ | (1) |
| $Ab_{ext}$ | $\frac{dAb_{ext}}{dt}=v_{1}-v_{4f}+v_{4r}$ | (2) |
| $PL_{ext}$ | $\frac{dPL_{ext}}{dt}=v_{1}\times DAR+v_{14f}-v_{14r}$ | (3) |
| $HER2$ | $\frac{dHER2}{dt}=v_{2}-v_{5f}+v_{5r}-v_{3f}+v_{3r}-v_{4f}+v_{4r}$ | (4) |
| $HER2:ADC$ | $\frac{dHER2:ADC}{dt}=v_{3f}-v_{3r}-v_{6f}+v_{6r}$ | (5) |
| $HER2:Ab$ | $\frac{dHER2:Ab}{dt}=v_{4f}-v_{4r}-v_{7f}+v_{7r}$ | (6) |
| $HER2_{endo}$ | $\frac{dHER2_{endo}}{dt}=v_{5f}-v_{5r}-v_{8}$ | (7) |
| $HER2:ADC_{endo}$ | $\frac{dHER{2:ADC}_{endo}}{dt}=v_{6f}-v_{6r}-v_{9}-v_{11}$ | (8) |
| $HER2:Ab_{endo}$ | $\frac{dHER{2:Ab}_{endo}}{dt}=v_{7f}-v_{7r}-v_{10}+v_{11}$ | (9) |
| $PL_{endo}$ | $\frac{dPL_{endo}}{dt}=(v_{9}+v_{11})\times DAR-v_{12}$ | (10) |
| $PL_{cyto}$ | $\frac{dPL_{cyto}}{dt}=v_{12}-v_{13f}+v_{13r}-v_{14f}+v_{14r}$ | (11) |
| $T_{cyto}$ | $\frac{dT_{cyto}}{dt}=-v_{13f}+v_{13r}$ | (12) |
| $T:PL_{cyto}$ | $\frac{dT_{cyto}}{dt}=v_{13f}-v_{13r}$ | (13) |

### Table S3c: Mouse model reactions

| **Reaction** | **Rate law** |
| --- | --- |
| $ADC_{central} \to Ab_{central} + DAR * PL_{central}$ | $v_{14}=k_{dec}ADC_{central}$ |
| $ADC_{central} \to DAR * PL_{central}$ | $v_{15}= \frac{ln(2)}{t_{½, Ab}}ADC_{central}$ |
| $Ab_{central} \to\emptyset$ | $v_{16}=$ $\frac{ln(2)}{t_{½, Ab}}Ab_{central}$ |
| $PL_{central} \to\emptyset$ | $v_{17}=\frac{ln(2)}{t_{½, PL}}PL_{central}$ |
| $ADC_{central}\leftrightarrow ADC_{peripheral}$ | $v_{18f}=k_{12, Ab}ADC_{central}$  $v_{18r}=k_{21, Ab}ADC_{peripheral}$ |
| $Ab_{central}\leftrightarrow Ab_{peripheral}$ | $v_{19f}=k_{12, Ab}Ab_{central}$  $v_{19r}=k_{21, Ab}Ab_{peripheral}$ |
| $PL_{central}\leftrightarrow PL_{peripheral}$ | $v_{20f}=k_{12, PL}PL_{central}$  $v_{20r}=k_{21, PL}PL_{peripheral}$ |
| $ADC_{peripheral} \to Ab_{peripheral} + DAR * PL_{peripheral}$ | $v_{21}=$ $k_{dec}ADC_{peripheral}$ |
| $ADC_{peripheral} \to DAR * PL_{peripheral}$ | $v_{22}=\frac{ln(2)}{t_{½, Ab}}ADC_{peripheral}$ |
| $Ab_{peripheral} \to\emptyset$ | $v_{23}=\frac{ln(2)}{t_{½, Ab}}Ab_{peripheral}$ |
| $PL_{peripheral} \to\emptyset$ | $v_{24}=\frac{ln(2)}{t_{½, PL}}PL_{peripheral}$ |
| $ADC_{central}\leftrightarrow ADC_{ext,tumor}$ | $v_{25f}=k_{13, Ab}ADC_{central}$  $v_{25r}=k_{31, Ab}ADC_{ext,tumor}$ |
| $Ab_{central}\leftrightarrow Ab_{ext,tumor}$ | $v_{26f}=k_{13, Ab}Ab_{central}$  $v_{26r}=k_{31, Ab}Ab_{ext,tumor}$ |
| $PL_{central}\leftrightarrow PL_{ext,tumor}$ | $v_{27f}=k_{13, PL}PL_{central}$  $v_{27r}=k_{31, PL}PL_{ext,tumor}$ |
| $ADC_{ext,tumor} \to Ab_{ext,tumor} + DAR * PL_{ext,tumor}$ | $v_{28}=$ $k_{dec}ADC_{ext,tumor}$ |
| $\emptyset\to HER2_{ext,N\{s\},tumor}$ | $v_{29}=k_{synth}N_{\{s\}}$ |
| $HER2_{ext,N\{s\},tumor}+ADC_{ext,tumor}\leftrightarrow HER2:ADC_{ext,N\{s\},tumor}$ | $v_{30f}=\frac{k_{on,Ab}}{V_{tumor,ext}\varepsilon_{Ab}}HER2_{ext,N\{s\},tumor}\times ADC_{ext,tumor}$  $v_{30r}=k_{off,Ab}HER2:ADC_{N\{s\},tumor}$ |
| $HER2_{ext,N\{s\},tumor}+Ab_{ext,tumor}\leftrightarrow HER2:Ab_{ext,N\{s\},tumor}$ | $v_{31f}=\frac{k_{on,Ab}}{V_{ext, Ab, tumor}}HER2_{ext,N\{s\},tumor}\times Ab_{ext,tumor}$  $v_{31r}={k_{on,PL}K_{D,PL}}HER2:Ab_{ext,N\{s\},tumor}$ |
| $HER2:ADC_{ext,N\{s\},tumor} \to HER2:Ab_{ext,N\{s\},tumor}$  $+ DAR * PL_{ext,tumor}$ | $v_{32}=$ $k_{dec}HER2:ADC_{ext,N\{s\},tumor}$ |
| $HER2_{ext,N\{s\},tumor}\leftrightarrow HER2_{endo,N\{s\},tumor}$ | $v_{33f}=k_{endo,HER2}HER2_{ext,N\{s\},tumor}$  $v_{33r}=k_{rec,HER2}HER2_{endo,N\{s\},tumor}$ |
| $HER2:ADC_{ext,N\{s\},tumor}\leftrightarrow HER{2:ADC}_{endo,N\{s\},tumor}$ | $v_{34f}=k_{endo, HER2:Ab}HER2:ADC_{ext,N\{s\},tumor}$  $v_{34r}=k_{rec, HER2:Ab}HER{2:ADC}_{endo,N\{s\},tumor}$ |
| $HER2:Ab_{ext,N\{s\},tumor}\leftrightarrow HER{2:Ab}_{endo,N\{s\},tumor}$ | $v_{35f}=k_{endo, HER2:Ab}HER2:Ab_{ext,N\{s\},tumor}$  $v_{35r}=k_{rec, HER2:Ab}HER{2:Ab}_{endo,N\{s\},tumor}$ |
| $HER2_{endo,N\{s\},tumor}\to\emptyset$ | $v_{36}=k_{deg,HER2}HER2_{endo,N\{s\},tumor}$ |
| $HER{2:ADC}_{endo,N\{s\},tumor}\to DAR*PL_{endo,N\{s\},tumor}$ | $v_{37}=k_{deg,HER2:Ab}HER{2:ADC}_{endo,N\{s\},tumor}$ |
| $HER{2:Ab}_{endo,N\{s\},tumor}\to\emptyset$ | $v_{38}=k_{deg,HER2:Ab}HER{2:Ab}_{endo,N\{s\},tumor}$ |
| $HER{2:ADC}_{endo,N\{s\},tumor}\to HER{2:Ab}_{endo,N\{s\},tumor}$  $+DAR*PL_{endo,N\{s\},tumor}$ | $v_{39}=k_{dec}HER{2:ADC}_{endo,N\{s\},tumor}$ |
| $PL_{endo,N\{s\},tumor}\to PL_{cyto,N\{s\},tumor}$ | $v_{40}=k_{in, PL}PL_{endo,N\{s\},tumor}$ |
| $T_{cyto,N\{s\},tumor}+PL_{cyto,N\{s\},tumor}\leftrightarrow T:PL_{cyto,N\{s\},tumor}$ | $v_{41f}=\frac{k_{on,PL}}{{Pc_{PL}V}_{int,tumor}}T_{cyto,N\{s\},tumor}PL_{cyto,N\{s\},tumor}$  $v_{41r}=k_{off,PL}T:PL_{cyto,N\{s\},tumor}$ |
| $\emptyset\to N_{1}$ | $v_{42}=k_{growth}N_{1}$ |
| $\emptyset\to HER2_{ext,N1,tumor}$ | $v_{43}=k_{growth}N_{1}RPC_{HER2,tumor}$ |
| $\emptyset\to HER2_{endo,N1,tumor}$ | $v_{44}=k_{growth}N_{1}RPC_{HER2,endo,tumor}$ |
| $\emptyset\to T_{cyto,N1,tumor}$ | $v_{45}=k_{growth}N_{1}V_{cell}T_{per,cell}$ |
| $N_{1}\to N_{2}$ | $v_{46}=kkill\times N_{1}$ |
| $N_{\{s\}}\to N_{\{s+1\}}, s=2, 3$ | $v_{47}={\frac{N_{\{s\}}}{\tau}}$ |
| $N_{4}\to\emptyset$ | $v_{48}={\frac{N_{4}}{\tau}}$ |
| ${HER2}_{ext,N1,tumor}\to HER2_{ext,N2,tumor}$ | $v_{49}=kkill\times{HER2}_{ext,N1,tumor}$ |
| $HER2_{ext,N\{s\},tumor}\to HER2_{ext,N\{s+1\},tumor}, s=2,3$ | $v_{50}=\frac{HER2_{ext,N\{s\},tumor}}{\tau}$ |
| ${HER2}_{ext,N4,tumor}\to\emptyset$ | $v_{51}=\frac{HER2_{ext,N4,tumor}}{\tau}$ |
| ${HER2:ADC}_{ext,N1,tumor}\to HER{2:ADC}_{ext,N2,tumor}$ | $v_{52}=kkill\times{HER2:ADC}_{ext,N1,tumor}$ |
| $HER{2:ADC}_{ext,N\{s\},tumor}\to HER{2:ADC}_{ext,N\{s+1\},tumor},$  $s=2,3$ | $v_{53}=\frac{HER{2:ADC}_{ext,N\{s\},tumor}}{\tau}$ |
| ${HER2:ADC}_{ext,N4,tumor}\to DAR*PL_{ext,tumor}$ | $v_{54}=\frac{HER{2:ADC}_{ext,N4,tumor}}{\tau}$ |
| ${HER2:Ab}_{ext,N1,tumor}\to HER{2:Ab}_{ext,N2,tumor}$ | $v_{55}=kkill\times{HER2:Ab}_{ext,N1,tumor}$ |
| $HER{2:Ab}_{ext,N\{s\},tumor}\to HER{2:Ab}_{ext,N\{s+1\},tumor}, s=2,3$ | $v_{56}=\frac{HER{2:Ab}_{ext,N\{s\},tumor}}{\tau}$ |
| ${HER2:Ab}_{ext,N4,tumor}\to\emptyset$ | $v_{57}=\frac{HER{2:Ab}_{ext,N4,tumor}}{\tau}$ |
| ${HER2}_{endo,N1,tumor}\to HER2_{endo,N2,tumor}$ | $v_{58}=kkill\times{HER2}_{endo,N1,tumor}$ |
| $HER2_{endo,N\{s\},tumor}\to HER2_{endo,N\{s+1\},tumor}, s=2,3$ | $v_{59}=\frac{HER2_{endo,N\{s\},tumor}}{\tau}$ |
| ${HER2}_{endo,N4,tumor}\to\emptyset$ | $v_{60}=\frac{HER2_{endo,N4,tumor}}{\tau}$ |
| ${HER2:ADC}_{endo,N1,tumor}\to HER{2:ADC}_{endo,N2,tumor}$ | $v_{61}=kkill\times{HER2:ADC}_{endo,N1,tumor}$ |
| $HER{2:ADC}_{endo,N\{s\},tumor}\to HER{2:ADC}_{endo,N\{s+1\},tumor},$  $s=2,3$ | $v_{62}=\frac{HER{2:ADC}_{endo,N\{s\},tumor}}{\tau}$ |
| ${HER2:ADC}_{endo,N4,tumor}\to DAR*PL_{ext,tumor}$ | $v_{63}=\frac{HER{2:ADC}_{endo,N4,tumor}}{\tau}$ |
| ${HER2:Ab}_{endo,N1,tumor}\to HER{2:Ab}_{endo,N2,tumor}$ | $v_{64}=kkill\times{HER2:Ab}_{endo,N1,tumor}$ |
| $HER{2:Ab}_{endo,N\{s\},tumor}\to HER{2:Ab}_{endo,N\{s+1\},tumor},$  $s=2,3$ | $v_{65}=\frac{HER{2:Ab}_{endo,N\{s\},tumor}}{\tau}$ |
| ${HER2:Ab}_{endo,N4,tumor}\to\emptyset$ | $v_{66}=\frac{HER{2:Ab}_{endo,N4,tumor}}{\tau}$ |
| ${PL}_{endo,N1,tumor}\to PL_{endo,N2,tumor}$ | $v_{67}=kkill\times{PL}_{endo,N1,tumor}$ |
| $PL_{endo,N\{s\},tumor}\to{PL}_{endo,N\{s+1\},tumor}, s=2,3$ | $v_{68}=\frac{PL_{endo,N\{s\},tumor}}{\tau}$ |
| ${PL}_{endo,N4,tumor}\to PL_{ext,tumor}$ | $v_{69}=\frac{{PL}_{endo,N4,tumor}}{\tau}$ |
| ${PL}_{cyto,N1,tumor}\to PL_{cyto,N2,tumor}$ | $v_{70}=kkill\times PL_{cyto,N1,tumor}$ |
| $PL_{cyto,N\{s\},tumor}\to{PL}_{cyto,N\{s+1\},tumor}, s=2,3$ | $v_{71}=\frac{PL_{cyto,N\{s\},tumor}}{\tau}$ |
| ${PL}_{cyto,N4,tumor}\to PL_{ext,tumor}$ | $v_{72}=\frac{{PL}_{cyto,N4,tumor}}{\tau}$ |
| $T_{cyto,N1,tumor}\to T_{cyto,N2,tumor}$ | $v_{73}=kkill\times T_{cyto,N1,tumor}$ |
| $T_{cyto,N\{s\},tumor}\to T_{cyto,N\{s+1\},tumor}, s=2,3$ | $v_{74}=\frac{T_{cyto,N\{s\},tumor}}{\tau}$ |
| $T_{cyto,N4,tumor}\to\emptyset$ | $v_{75}=\frac{T_{cyto,N4,tumor}}{\tau}$ |
| ${T:PL}_{cyto,N1,tumor}\to T:PL_{cyto,N2,tumor}$ | $v_{76}=kkill\times{T:PL}_{cyto,N1,tumor}$ |
| $T:PL_{cyto,N\{s\},tumor}\to{T:PL}_{cyto,N\{s+1\},tumor}, s=2,3$ | $v_{77}=\frac{T:PL_{cyto,N\{s\},tumor}}{\tau}$ |
| ${T:PL}_{cyto,N4,tumor}\to PL_{ext,tumor}$ | $v_{78}=\frac{{T:PL}_{cyto,N4,tumor}}{\tau}$ |
| $PL_{ext,tumor}\leftrightarrow PL_{cyto,N\{s\},tumor}$ | $v_{79f}=k_{in,PL}PL_{ext,tumor}$  $v_{79r}=k_{out,PL}PL_{cyto,N\{s\},tumor}$ |

where s=1,2,3,4 unless noted.

### Table S3d: Resulting mouse model ODEs

| **State** | **ODE** | **Equation** |
| --- | --- | --- |
| $ADC_{central}$ | $\frac{dADC_{central}}{dt}=-v_{14}-v_{15}-v_{18f}+v_{18r}-v_{25f}+v_{25r}$ | (14) |
| $Ab_{central}$ | $\frac{dAb_{central}}{dt}=v_{14}-v_{16}-v_{19f}+v_{19r}-v_{26f}+v_{26r}$ | (15) |
| $PL_{central}$ | $\frac{dPL_{central}}{dt}=v_{14}\times DAR+v_{15}\times DAR-v_{17}-v_{20f}+v_{20r}-v_{27f}+v_{27r}$ | (16) |
| $ADC_{peripheral}$ | $\frac{dADC_{peripheral}}{dt}=v_{18f}-v_{18r}-v_{21}-v_{22}$ | (17) |
| $Ab_{peripheral}$ | $\frac{dAb_{peripheral}}{dt}=v_{19f}-v_{19r}+v_{21}-v_{23}$ | (18) |
| $PL_{peripheral}$ | $\frac{dPL_{peripheral}}{dt}=v_{20f}-v_{20r}+v_{21}\times DAR+v_{22}\times DAR-v_{24}$ | (19) |
| $ADC_{ext,tumor}$ | $\frac{dADC_{ext,tumor}}{dt}=v_{25f}-v_{25r}-v_{28}-\sum v_{30f,N\{s\}}+\sum v_{30r,N\{s\}}$ | (20) |
| $Ab_{ext,tumor}$ | $\frac{dAb_{ext,tumor}}{dt}={v_{26f}}-v_{26r}+v_{28}-\sum v_{31f,N\{s\}}+\sum v_{31r,N\{s\}}$ | (21) |
| $PL_{ext,tumor}$ | $\frac{dPL_{ext,tumor}}{dt}=v_{27f}-v_{27r}+v_{28}\times DAR+\sum v_{32,N\{s\}}\times DAR+v_{54}\times DAR$  $+v_{63}\times DAR+v_{69}+v_{72}+v_{78}-v_{79f}+\sum v_{79r,N\{s\}}$ | (22) |
| $N1$ | $\frac{dN1}{dt}=v_{42}-v_{46}$ | (23) |
| $N2$ | $\frac{dN2}{dt}=v_{46}-v_{47,N2}$ | (24) |
| $N3$ | $\frac{dN3}{dt}=v_{47,N2}-v_{47,N3}$ | (25) |
| $N4$ | $\frac{dN4}{dt}=v_{47,N3}-v_{48}$ | (26) |
| $HER2_{ext,N1,tumor}$ | $\frac{dHER2_{ext,N1,tumor}}{dt}=v_{29,N1}{-v}_{30f,N1}+v_{30r,N1}-v_{31f,N1}+v_{31r,N1}-v_{33f,N1}+v_{33r,N1}$  $+v_{43}-v_{49}$ | (27) |
| $HER2_{ext,N2,tumor}$ | $\frac{dHER2_{ext,N2,tumor}}{dt}=v_{29,N2}{-v}_{30f,N2}+v_{30r,N2}-v_{31f,N2}+v_{31r,N2}-v_{33f,N2}+v_{33r,N2}$  $+v_{49}-v_{50,N2}$ | (28) |
| $HER2_{ext,N3,tumor}$ | $\frac{dHER2_{ext,N3,tumor}}{dt}={v_{29,N3}{-v}_{30f,N3}+v_{30r,N3}-v_{31f,N3}+v_{31r,N3}-v_{33f,N3}+v_{33r,N3}}$  $+v_{50,N2}-v_{50,N3}$ | (29) |
| $HER2_{ext,N4,tumor}$ | $\frac{dHER2_{ext,N4,tumor}}{dt}={v_{29,N4}{-v}_{30f,N4}+v_{30r,N4}-v_{31f,N4}+v_{31r,N4}-v_{33f,N4}+v_{33r,N4}}$  $+v_{50,N3}-v_{51}$ | (30) |
| $HER{2:ADC}_{ext,N1,tumor}$ | $\frac{dHER{2:ADC}_{ext,N1,tumor}}{dt}=v_{30f,N1}-v_{30r,N1}-v_{32,N1} {-v}_{34f,N1}+v_{34r,N1}-v_{52}$ | (31) |
| $HER{2:ADC}_{ext,N2,tumor}$ | $\frac{dHER{2:ADC}_{ext,N2,tumor}}{dt}=v_{30f,N2}-v_{30r,N2}-v_{32,N2}{-v}_{34f,N2}+v_{34r,N2}+v_{52}-v_{53,N2}$ | (32) |
| $HER{2:ADC}_{ext,N3,tumor}$ | $\frac{dHER{2:ADC}_{ext,N3,tumor}}{dt}=v_{30f,N3}-v_{30r,N3}-v_{32,N3}{-v}_{34f,N3}+v_{34r,N3}+v_{53,N2}-v_{53,N3}$ | (33) |
| $HER{2:ADC}_{ext,N4,tumor}$ | $\frac{dHER{2:ADC}_{ext,N4,tumor}}{dt}=v_{30f,N4}-v_{30r,N4}-v_{32,N4}{-v}_{34f,N4}+v_{34r,N4}+v_{53,N3}-v_{54}$ | (34) |
| $HER{2:Ab}_{ext,N1,tumor}$ | $\frac{dHER{2:Ab}_{ext,N1,tumor}}{dt}=v_{31f,N1}-v_{31r,N1}+v_{32,N1}{-v}_{35f,N1}+v_{35r,N1}-v_{55}$ | (35) |
| $HER{2:Ab}_{ext,N2,tumor}$ | $\frac{dHER{2:Ab}_{ext,N2,tumor}}{dt}=v_{31f,N2}-v_{31r,N2}+v_{32,N2}{-v}_{35f,N2}+v_{35r,N2}+v_{55}-v_{56,N2}$ | (36) |
| $HER{2:Ab}_{ext,N3,tumor}$ | $\frac{dHER{2:Ab}_{ext,N3,tumor}}{dt}=v_{31f,N3}-v_{31r,N3}+v_{32,N3}{-v}_{35f,N3}+v_{35r,N3}+v_{56,N2}-v_{56,N3}$ | (37) |
| $HER{2:Ab}_{ext,N4,tumor}$ | $\frac{dHER{2:Ab}_{ext,N4,tumor}}{dt}=v_{31f,N4}-v_{31r,N4}+v_{32,N4}{-v}_{35f,N4}+v_{35r,N4}+v_{56,N3}-v_{57}$ | (38) |
| $HER2_{endo,N1,tumor}$ | $\frac{dHER2_{endo,N1,tumor}}{dt}=v_{33f,N1}-v_{33r,N1}-v_{36,N1}{+v}_{44}-v_{58}$ | (39) |
| $HER2_{endo,N2,tumor}$ | $\frac{dHER2_{endo,N2,tumor}}{dt}=v_{33f,N2}-v_{33r,N2}-v_{36,N2}+v_{58}-v_{59,N2}$ | (40) |
| $HER2_{endo,N3,tumor}$ | $\frac{dHER2_{endo,N3,tumor}}{dt}=v_{33f,N3}-v_{33r,N3}-v_{36,N3}+v_{59,N2}-v_{59,N3}$ | (41) |
| $HER2_{endo,N4,tumor}$ | $\frac{dHER2_{endo,N4,tumor}}{dt}=v_{33f,N4}-v_{33r,N4}-v_{36,N4}+v_{59,N3}-v_{60}$ | (42) |
| $HER{2:ADC}_{endo,N1,tumor}$ | $\frac{dHER{2:ADC}_{endo,N1,tumor}}{dt}=v_{34f,N1}-v_{34r,N1}-v_{37,N1}{-v}_{39,N1}-v_{61}$ | (43) |
| $HER{2:ADC}_{endo,N2,tumor}$ | $\frac{dHER{2:ADC}_{endo,N2,tumor}}{dt}=v_{34f,N2}-v_{34r,N2}-v_{37,N2}{-v}_{39,N2}+v_{61}-v_{62,N2}$ | (44) |
| $HER{2:ADC}_{endo,N3,tumor}$ | $\frac{dHER{2:ADC}_{endo,N3,tumor}}{dt}=v_{34f,N3}-v_{34r,N3}-v_{37,N3}{-v}_{39,N3}+v_{62,N2}-v_{62,N3}$ | (45) |
| $HER{2:ADC}_{endo,N4,tumor}$ | $\frac{dHER{2:ADC}_{endo,N4,tumor}}{dt}=v_{34f,N4}-v_{34r,N4}-v_{37,N4}{-v}_{39,N4}+v_{62,N3}-v_{63}$ | (46) |
| $HER{2:Ab}_{endo,N1,tumor}$ | $\frac{dHER{2:Ab}_{endo,N1,tumor}}{dt}=v_{35f,N1}-v_{35r,N1}-v_{38,N1}{+v}_{39,N1}-v_{64}$ | (47) |
| $HER{2:Ab}_{endo,N2,tumor}$ | $\frac{dHER{2:Ab}_{endo,N2,tumor}}{dt}=v_{35f,N2}-v_{35r,N2}-v_{38,N2}{+v}_{39,N2}+v_{64}-v_{65,N2}$ | (48) |
| $HER{2:Ab}_{endo,N3,tumor}$ | $\frac{dHER{2:Ab}_{endo,N3,tumor}}{dt}=v_{35f,N3}-v_{35r,N3}-v_{38,N3}{+v}_{39,N3}+v_{65,N2}-v_{65,N3}$ | (49) |
| $HER{2:Ab}_{endo,N4,tumor}$ | $\frac{dHER{2:Ab}_{endo,N4,tumor}}{dt}=v_{35f,N4}-v_{35r,N4}-v_{38,N4}{+v}_{39,N4}+v_{65,N3}-v_{66}$ | (50) |
| $PL_{endo,N1,tumor}$ | $\frac{dPL_{endo,N1,tumor}}{dt}=v_{37,N1}\times DAR+v_{39,N1}\times DAR-v_{40,N1}-v_{67}$ | (51) |
| $PL_{endo,N2,tumor}$ | $\frac{dPL_{endo,N2,tumor}}{dt}=v_{37,N2}\times DAR+v_{39,N2}\times DAR-v_{40,N2}+v_{67}-v_{68,N2}$ | (52) |
| $PL_{endo,N3,tumor}$ | $\frac{dPL_{endo,N3,tumor}}{dt}=v_{37,N3}\times DAR+v_{39,N3}\times DAR-v_{40,N3}+v_{68,N2}-v_{68,N3}$ | (53) |
| $PL_{endo,N4,tumor}$ | $\frac{dPL_{endo,N4,tumor}}{dt}=v_{37,N4}\times DAR+v_{39,N4}\times DAR-v_{40,N4}+v_{68,N3}-v_{69}$ | (54) |
| $PL_{cyto,N1,tumor}$ | $\frac{dPL_{cyto,N1,tumor}}{dt}=v_{40,N1}-v_{41f,N1}+v_{41r,N1}-v_{70}+v_{79f}-v_{79r,N1}$ | (55) |
| $PL_{cyto,N2,tumor}$ | $\frac{dPL_{cyto,N2,tumor}}{dt}=v_{40,N2}-v_{41f,N2}+v_{41r,N2}+v_{70}-v_{71,N2}+v_{79f}-v_{79r,N2}$ | (56) |
| $PL_{cyto,N3,tumor}$ | $\frac{dPL_{cyto,N3,tumor}}{dt}=v_{40,N3}-v_{41f,N3}+v_{41r,N3}+v_{71,N2}-v_{71,N3}+v_{79f}-v_{79r,N3}$ | (57) |
| $PL_{cyto,N4,tumor}$ | $\frac{dPL_{cyto,N4,tumor}}{dt}=v_{40,N4}-v_{41f,N4}+v_{41r,N4}+v_{71,N3}-v_{72}+v_{79f}-v_{79r,N4}$ | (58) |
| $T_{cyto,N1,tumor}$ | $\frac{dT_{cyto,N1,tumor}}{dt}=-v_{41f,N1}+v_{41r,N1}+v_{45}-v_{73}$ | (59) |
| $T_{cyto,N2,tumor}$ | $\frac{dT_{cyto,N2,tumor}}{dt}=-v_{41f,N2}+v_{41r,N2}+v_{73}-v_{74,N2}$ | (60) |
| $T_{cyto,N3,tumor}$ | $\frac{dT_{cyto,N3,tumor}}{dt}=-v_{41f,N3}+v_{41r,N3}+v_{74,N2}-v_{74,N3}$ | (61) |
| $T_{cyto,N4,tumor}$ | $\frac{dT_{cyto,N4,tumor}}{dt}=-v_{41f,N4}+v_{41r,N4}+v_{74,N3}-v_{75}$ | (62) |
| $T:PL_{endo,N1,tumor}$ | $\frac{dT:PL_{endo,N1,tumor}}{dt}=v_{41f,N1}-v_{41r,N1}-v_{76}$ | (63) |
| $T:PL_{endo,N2,tumor}$ | $\frac{dT:PL_{endo,N2,tumor}}{dt}=v_{41f,N2}-v_{41r,N2}+v_{76}-v_{77,N2}$ | (64) |
| $T:PL_{endo,N3,tumor}$ | $\frac{dT:PL_{endo,N3,tumor}}{dt}=v_{41f,N3}-v_{41r,N3}+v_{77,N2}-v_{77,N3}$ | (65) |
| $T:PL_{endo,N4,tumor}$ | $\frac{dT:PL_{endo,N4,tumor}}{dt}=v_{41f,N4}-v_{41r,N4}+v_{77,N3}-v_{78}$ | (66) |

### Table S3e: Additional human model reactions

| **Reaction** | **Rate law** |
| --- | --- |
| $\emptyset\to HER2_{central}$ | $v_{78}=k_{synth,HER2}N_{cell, central}$ |
| $HER2_{central}+ADC_{central}\leftrightarrow HER2:ADC_{central}$ | $v_{79f}=\frac{k_{on, Ab}}{V_{central}}HER2_{central}ADC_{central}$  $v_{79r}=k_{off,Ab}HER2:ADC_{central}$ |
| $HER2_{central}+Ab_{central}\leftrightarrow HER2:Ab_{central}$ | $v_{80f}=\frac{k_{on,Ab}}{V_{central}}HER2_{central}Ab_{central}$  $v_{80r}=k_{off,Ab}HER2:Ab_{central}$ |
| $HER2_{central}\leftrightarrow HER2_{endo, central}$ | $v_{81f}=k_{endo, HER2}HER2_{central}$  $v_{81r}=k_{rec, HER2} HER2_{endo, central}$ |
| $HER{2:ADC}_{central}\leftrightarrow HER{2:ADC}_{endo, central}$ | $v_{82f}=k_{endo, HER2:Ab}HER2:ADC_{central}$  $v_{82r}=k_{rec, HER2:Ab} HER{2:ADC}_{endo, central}$ |
| $HER{2:Ab}_{central}\leftrightarrow HER{2:Ab}_{endo, central}$ | $v_{83f}=k_{endo, HER2:Ab}HER2:Ab_{central}$  $v_{83r}=k_{rec, HER2:Ab} HER{2:Ab}_{central}$ |
| $HER2_{endo, central}\to\emptyset$ | $v_{84}=k_{deg, HER2}HER2_{endo,central}$ |
| $HER{2:ADC}_{endo, central}\to\emptyset$ | $v_{85}=k_{deg, HER2, Ab}HER{2:ADC}_{endo,central}$ |
| $HER{2:Ab}_{endo, central}\to\emptyset$ | $v_{86}=k_{deg,HER2, Ab}HER{2:Ab}_{endo,central}$ |
| $\emptyset\to HER2_{peripheral}$ | $v_{87}=k_{synth,HER2}N_{cell, peripheral}$ |
| $HER2_{peripheral}+ADC_{peripheral}\leftrightarrow HER2:ADC_{peripheral}$ | $v_{88f}=\frac{k_{on, Ab}}{V_{peripheral}}HER2_{peripheral}ADC_{peripheral}$  $v_{88r}=k_{off,Ab}HER2:ADC_{peripheral}$ |
| $HER2_{peripheral}+Ab_{peripheral}\leftrightarrow HER2:Ab_{peripheral}$ | $v_{89f}=\frac{k_{on,Ab}}{V_{central}}HER2_{peripheral}Ab_{peripheral}$  $v_{89r}=k_{off,Ab}HER2:Ab_{peripheral}$ |
| $HER2_{peripheral}\leftrightarrow HER2_{endo, peripheral}$ | $v_{90f}=k_{endo, HER2}HER2_{peripheral}$  $v_{90r}=k_{rec, HER2} HER2_{endo, peripheral}$ |
| $HER{2:ADC}_{peripheral}\leftrightarrow HER{2:ADC}_{endo, peripheral}$ | $v_{91f}=k_{endo, HER2:Ab}HER2:ADC_{peripheral}$  $v_{91r}=k_{rec, HER2:Ab} HER{2:ADC}_{endo, peripheral}$ |
| $HER{2:Ab}_{peripheral}\leftrightarrow HER{2:Ab}_{endo, peripheral}$ | $v_{92f}=k_{endo, HER2:Ab}HER2:Ab_{peripheral}$  $v_{92r}=k_{rec, HER2:Ab} HER{2:Ab}_{peripheral}$ |
| $HER2_{endo, peripheral}\to\emptyset$ | $v_{93}=k_{deg, HER2}HER2_{endo,peripheral}$ |
| $HER{2:ADC}_{endo, peripheral}\to\emptyset$ | $v_{94}=k_{deg, HER2, Ab}HER{2:ADC}_{endo,peripheral}$ |
| $HER{2:Ab}_{endo, peripheral}\to\emptyset$ | $v_{95}=k_{deg,HER2, Ab}HER{2:Ab}_{endo,peripheral}$ |
| $HER2_{central} \to sHER2_{central}$ | $v_{96}=k_{shed}HER2_{central}$ |
| $sHER2_{central} \to\emptyset$ | $v_{97}=k_{deg,sHER2}HER2_{central}$ |
| $HER{2:ADC}_{central} \to sHER{2:ADC}_{central}$ | $v_{98}=k_{shed, sHER2}\chi_{shed, HER2:Ab}HER{2:ADC}_{central}$ |
| $HER{2:Ab}_{central} \to sHER{2:Ab}_{central}$ | $v_{99}=k_{shed, sHER2}\chi_{shed, HER2:Ab}HER{2:Ab}_{central}$ |
| $sHER{2:ADC}_{central}\to DAR*PL_{central}$ | $v_{100}=k_{deg, sHER2,Ab}sHER{2:ADC}_{central}$ |
| $sHER{2:Ab}_{central} \to\emptyset$ | $v_{101}=k_{deg, sHER2,Ab}sHER{2:Ab}_{central}$ |
| $sHER2_{central}+ADC_{central}\leftrightarrow sHER2:ADC_{central}$ | $v_{102f}=\frac{k_{on,Ab}}{V_{central}}sHER2_{central}ADC_{central}$  $v_{102r}=k_{off,Ab}sHER2:ADC_{central}$ |
| $sHER2_{central}+Ab_{central}\leftrightarrow sHER2:Ab_{central}$ | $v_{103f}=\frac{k_{on,Ab}}{V_{central}}sHER2_{central}Ab_{central}$  $v_{103r}=k_{off,Ab}sHER2:Ab_{central}$ |
| $HER2_{peripheral} \to sHER2_{peripheral}$ | $v_{104}=k_{shed}HER2_{peripheral}$ |
| $sHER2_{peripheral} \to\emptyset$ | $v_{105}=k_{deg,sHER2}HER2_{peripheral}$ |
| $HER{2:ADC}_{peripheral} \to sHER{2:ADC}_{peripheral}$ | $v_{106}=k_{shed, sHER2}\chi_{shed, HER2:Ab}HER{2:ADC}_{peripheral}$ |
| $HER{2:Ab}_{peripheral} \to sHER{2:Ab}_{peripheral}$ | $v_{107}=k_{shed, sHER2}\chi_{shed, HER2:Ab}HER{2:Ab}_{peripheral}$ |
| $sHER{2:ADC}_{peripheral}\to DAR*PL_{peripheral}$ | $v_{108}=k_{deg, sHER2,Ab}sHER{2:ADC}_{peripheral}$ |
| $HER2:Ab_{peripheral} \to\emptyset$ | $v_{109}=k_{deg, sHER2:Ab}sHER{2:Ab}_{peripheral}$ |
| $sHER2_{peripheral}+ADC_{peripheral}\leftrightarrow sHER2:ADC_{peripheral}$ | $v_{110f}=\frac{k_{on,Ab}}{V_{peripheral}}sHER2_{peripheral}ADC_{peripheral}$  $v_{110r}=k_{off,Ab}sHER2:ADC_{peripheral}$ |
| $sHER2_{peripheral}+Ab_{peripheral}\leftrightarrow HER2:Ab_{peripheral}$ | $v_{111f}=\frac{k_{on,Ab}}{V_{peripheral}}sHER2_{peripheral}Ab_{peripheral}$  $v_{111r}=k_{off,Ab}sHER2:Ab_{peripheral}$ |
| $sHER2_{central}\leftrightarrow sHER2_{peripheral}$ | $v_{112f}=k_{12,sHER2}sHER2_{central}$  $v_{112r}=k_{21,sHER2}sHER2_{peripheral}$ |
| $sHER2_{central}\leftrightarrow sHER2_{tumor}$ | $v_{113f}=k_{13,sHER2}sHER2_{central}$  $v_{113r}=k_{31,sHER2}sHER2_{tumor}$ |
| $sHER{2:ADC}_{central}\leftrightarrow sHER{2:ADC}_{peripheral}$ | $v_{114f}=k_{12,sHER2:Ab}sHER2:ADC_{central}$  $v_{114r}=k_{21,sHER2:Ab}sHER2:ADC_{peripheral}$ |
| $sHER{2:ADC}_{central}\leftrightarrow sHER{2:ADC}_{tumor}$ | $v_{115f}=k_{13,sHER2:Ab}sHER2:ADC_{central}$  $v_{115r}=k_{31,sHER2:Ab}sHER2:ADC_{tumor}$ |
| $sHER{2:Ab}_{central}\leftrightarrow sHER{2:Ab}_{peripheral}$ | $v_{116f}=k_{12,sHER2:Ab}sHER2:Ab_{central}$  $v_{116r}=k_{21,sHER2:Ab}sHER2:Ab_{peripheral}$ |
| $sHER{2:Ab}_{central}\leftrightarrow sHER{2:Ab}_{tumor}$ | $v_{117f}=k_{12,sHER2:Ab}sHER2:Ab_{central}$  $v_{117r}=k_{21,sHER2:Ab}sHER2:Ab_{tumor}$ |
| $HER2_{N\{s\},tumor.} \to sHER2_{tumor}$ | $v_{118,N\{s\}}=k_{shed}HER2_{N\{s\},tumor}$ |
| $HER{2:Ab}_{N\{s\},tumor.} \to sHER{2:Ab}_{tumor}$ | $v_{119}=k_{shed}\chi_{shed, HER2:Ab}HER{2:Ab}_{N\{s\},tumor}$ |
| $HER{2:ADC}_{N\{s\},tumor.} \to sHER{2:ADC}_{tumor}$ | $v_{120}=k_{shed}\chi_{shed, HER2:Ab}HER{2:ADC}_{N\{s\},tumor}$ |
| $sHER2_{tumor}+ADC_{ext,tumor}\leftrightarrow sHER2:ADC_{tumor}$ | $v_{121f}=\frac{k_{on,Ab}}{V_{ext, Ab, tumor}}sHER2_{tumor}ADC_{ext, tumor}$  $v_{121r}=k_{off,Ab}sHER2:ADC_{tumor}$ |
| $sHER2_{tumor}+Ab_{ext, tumor}\leftrightarrow HER2:Ab_{tumor}$ | $v_{122f}=\frac{k_{on,Ab}}{V_{ext, Ab, tumor}}sHER2_{tumor}Ab_{ext, tumor}$  $v_{122r}=k_{off,Ab}sHER2:Ab_{tumor}$ |
| $sHER{2:ADC}_{central}\to sHER{2:Ab}_{central}+DAR*PL_{central}$ | $v_{123}=k_{dec}sHER{2:ADC}_{central}$ |
| $sHER{2:ADC}_{peripheral}\to sHER{2:Ab}_{peripheral}+DAR*PL_{peripheral}$ | $v_{124}=k_{dec}sHER{2:ADC}_{peripheral}$ |
| $sHER{2:ADC}_{tumor}\to sHER{2:Ab}_{tumor}+DAR*PL_{tumor}$ | $v_{125}=k_{dec}sHER{2:ADC}_{tumor}$ |
| $HER{2:ADC}_{central}\to HER{2:Ab}_{central}+DAR*PL_{central}$ | $v_{126}=k_{dec}HER{2:ADC}_{central}$ |
| $HER{2:ADC}_{peripheral}\to HER{2:Ab}_{peripheral}+DAR*PL_{peripheral}$ | $v_{127}=k_{dec}HER{2:ADC}_{peripheral}$ |

### Table S3f: Resulting human model ODEs (additional or changed from mouse model)

| **State** | **ODE** | **Eqn** |
| --- | --- | --- |
| $ADC_{central}$ | $\frac{dADC_{central}}{dt}=-v_{14}-v_{15}-v_{18f}+v_{18r}-v_{25f}+v_{25r}-v_{79f}+v_{79r}-v_{102f}+v_{102r}$ | (67) |
| $Ab_{central}$ | $\frac{dAb_{central}}{dt}=v_{14}-v_{16}-v_{19f}+v_{19r}-v_{26f}+v_{26r}-v_{80f}+v_{80r}-v_{103f}+v_{103r}$ | (68) |
| $PL_{central}$ | $\frac{dPL_{central}}{dt}=v_{14}\times DAR+v_{15}\times DAR-v_{17}-v_{20f}+v_{20r}-v_{27f}+v_{27r}$  ${+ v}_{126}\times DAR + v_{123}\times DAR$ | (69) |
| $HER2_{central}$ | $\frac{dHER2_{central}}{dt}=v_{78}-v_{79f}+v_{79r}-v_{80f}+v_{80r}-v_{81f}+v_{81r}$ | (70) |
| $HER{2:ADC}_{central}$ | $\frac{dHER{2:ADC}_{central}}{dt}=v_{79f}-v_{79r}-v_{82f}+v_{82r}-v_{126}$ | (71) |
| $HER{2:Ab}_{central}$ | $\frac{dHER{2:Ab}_{central}}{dt}=v_{80f}-v_{80r}-v_{83f}+v_{83r}+v_{126}$ | (72) |
| $HER2_{endo,central}$ | $\frac{dHER2_{endo, central}}{dt}=v_{81f}-v_{81r}-v_{84}$ | (73) |
| $HER{2:ADC}_{endo,central}$ | $\frac{dHER{2:ADC}_{endo, central}}{dt}=v_{82f}-v_{82r}-v_{85}$ | (74) |
| $HER{2:Ab}_{endo,central}$ | $\frac{dHER{2:Ab}_{endo, central}}{dt}=v_{83f}-v_{83r}-v_{86}$ | (75) |
| $sHER2_{central}$ | $\frac{dsHER2_{central}}{dt}=v_{96}-v_{97}-v_{102f}+v_{102r}-v_{103f}+v_{103r}-v_{112f}+v_{112r}-v_{113f}+v_{113r}$ | (76) |
| $sHER{2:ADC}_{central}$ | $\frac{dsHER{2:ADC}_{central}}{dt}={-v_{100}+v}_{102f}-v_{102r}-v_{114f}+v_{114r}-v_{123}$ | (77) |
| $sHER{2:Ab}_{central}$ | $\frac{dsHER{2:Ab}_{central}}{dt}={-v_{101}+v}_{103f}-v_{103r}-v_{116f}+v_{116r}$ | (78) |
| $ADC_{peripheral}$ | $\frac{dADC_{peripheral}}{dt}=v_{18f}-v_{18r}-v_{21}-v_{22}-v_{88f}+v_{88r}-v_{110f}+v_{110r}$ | (79) |
| $Ab_{peripheral}$ | $\frac{dAb_{peripheral}}{dt}=v_{19f}-v_{19r}+v_{21}-v_{23}-v_{89f}+v_{89r}-v_{111f}+v_{111r}$ | (80) |
| $PL_{peripheral}$ | $\frac{dPL_{peripheral}}{dt}=v_{20f}-v_{20r}+v_{21}\times DAR+v_{22}\times DAR-v_{24}+v_{124}\times DAR + v_{127}\times DAR$ | (81) |
| $HER2_{peripheral}$ | $\frac{dHER2_{peripheral}}{dt}=v_{87}-v_{88f}+v_{88r}-v_{89f}+v_{89r}-v_{90f}+v_{90r}$ | (82) |
| $HER{2:ADC}_{peripheral}$ | $\frac{dHER{2:ADC}_{peripheral}}{dt}=v_{88f}-v_{88r}-v_{91f}+v_{91r}-v_{127}$ | (83) |
| $HER{2:Ab}_{peripheral}$ | $\frac{dHER{2:Ab}_{peripheral}}{dt}=v_{89f}-v_{89r}-v_{92f}+v_{92r}+v_{127}$ | (84) |
| $HER2_{endo,peripheral}$ | $\frac{dHER2_{endo, peripheral}}{dt}=v_{90f}-v_{90r}-v_{93}$ | (85) |
| $HER{2:ADC}_{endo,peripheral}$ | $\frac{dHER{2:ADC}_{endo, peripheral}}{dt}=v_{91f}-v_{91r}-v_{94}$ | (86) |
| $HER{2:Ab}_{endo,peripheral}$ | $\frac{dHER{2:Ab}_{endo, peripheral}}{dt}=v_{92f}-v_{92r}-v_{95}$ | (87) |
| $sHER2_{peripheral}$ | $\frac{dsHER2_{peripheral}}{dt}=v_{104}-v_{105}-v_{110f}+v_{110r}-v_{111f}+v_{111r}+v_{112f}-v_{112r}$ | (88) |
| $sHER{2:ADC}_{peripheral}$ | $\frac{dsHER{2:ADC}_{peripheral}}{dt}={-v_{108}+v}_{110f}-v_{110r}+v_{114f}-v_{114r}-v_{124}$ | (89) |
| $sHER{2:Ab}_{peripheral}$ | $\frac{dsHER{2:Ab}_{central}}{dt}={-v_{109}+v}_{111f}-v_{111r}+v_{116f}-v_{116r}$ | (90) |
| $ADC_{ext,tumor}$ | $\frac{dADC_{ext,tumor}}{dt}=v_{25f}-v_{25r}-v_{28}-\sum v_{30f,N\{s\}}+\sum v_{30r,N\{s\}}-v_{121f}+v_{121r}$ | (91) |
| $Ab_{ext,tumor}$ | $\frac{dAb_{ext,tumor}}{dt}={v_{26f}}-v_{26r}+v_{28}-\sum v_{31f,N\{s\}}+\sum v_{31r,N\{s\}}-v_{122f}+v_{122r}-v_{122f}+v_{122r}$ | (92) |
| $PL_{ext,tumor}$ | $\frac{dPL_{ext,tumor}}{dt}=v_{27f}-v_{27r}+v_{28}\times DAR+\sum v_{32,N\{s\}}\times DAR+v_{54}\times DAR$  $+v_{63}\times DAR+v_{69}+v_{72}+v_{78}-v_{79f}+\sum v_{79r,N\{s\}}+k_{125}\times DAR$ | (93) |
| $sHER2_{tumor}$ | $\frac{dsHER2_{tumor}}{dt}=\sum v_{118,N\{s\}}-v_{121f}+v_{121r}+v_{113f}-v_{113r}$ | (94) |
| $sHER{2:ADC}_{tumor}$ | $\frac{dsHER{2:ADC}_{tumor}}{dt}=v_{121f}-v_{121r}+v_{115f}-v_{115r}-v_{125}$ | (95) |
| $sHER{2:Ab}_{tumor}$ | $\frac{dsHER{2:Ab}_{tumor}}{dt}=v_{122f}-v_{122r}+v_{117f}-v_{117r}+v_{125}$ | (96) |

### Table S4: Types of model parameters and sources for their values

| **Antibody-related parameters** | **Target-related parameters** | **Payload-related parameters** |
| --- | --- | --- |
| - Target binding affinity - First-order clearance - Antibody-target Internalization rate - MW and valency - Dose / schedule / route | - Receptor expression (whole body and tumor) - Receptor turnover rate - Ligand expression (whole body and tumor) - Ligand turnover rate - Receptor-ligand affinity - Soluble/shed receptor concentration - Soluble target turnover - Soluble target shed / synthesis biology - Internalization rate | - DAR - Binding affinity for intracellular target - Concentration of intracellular target - Payload permeability/partition coefficient - Linker cleavage rate - Payload efflux rate |

**Bibliography**

1. [Erickson HK, Lewis Phillips GD, Leipold DD, et al (2012) The Effect of Different Linkers on Target Cell Catabolism and Pharmacokinetics/Pharmacodynamics of Trastuzumab Maytansinoid Conjugates. Mol Cancer Ther 11:1133–1142](http://paperpile.com/b/z7MHEw/CEZ3v)

2. [FDA (2013) Kadcyla Pharmacology Review BLA. 1–5](http://paperpile.com/b/z7MHEw/eEFec)

3. [Schlosshauer M, Baker D (2004) Realistic protein-protein association rates from a simple diffusional model neglecting long-range interactions, free energy barriers, and landscape ruggedness. Protein Sci 13:1660–1669](http://paperpile.com/b/z7MHEw/e2xpO)

4. [Austin CD, De Mazière AM, Pisacane PI, et al (2004) Endocytosis and sorting of ErbB2 and the site of action of cancer therapeutics trastuzumab and geldanamycin. Mol Biol Cell 15:5268–5282](http://paperpile.com/b/z7MHEw/1MDjR)

5. [Pereira PMR, Sharma SK, Carter LM, et al (2018) Caveolin-1 mediates cellular distribution of HER2 and affects trastuzumab binding and therapeutic efficacy. Nat Commun 9:5137](http://paperpile.com/b/z7MHEw/ohbt3)

6. [Frost J, Galdeano C, Soares P, et al (2016) Potent and selective chemical probe of hypoxic signalling downstream of HIF-α hydroxylation via VHL inhibition. Nat Commun 7:13312](http://paperpile.com/b/z7MHEw/r6ObW)

7. [Guan Y, Shan X, Zhang F, et al (2015) Kinetics of small molecule interactions with membrane proteins in single cells measured with mechanical amplification. Sci Adv 1:e1500633](http://paperpile.com/b/z7MHEw/R60iQ)

8. [Lopus M, Oroudjev E, Wilson L, et al (2010) Maytansine and cellular metabolites of antibody-maytansinoid conjugates strongly suppress microtubule dynamics by binding to microtubules. Mol Cancer Ther 9:2689–2699](http://paperpile.com/b/z7MHEw/C0Tqn)

9. [Shah DK, Haddish-Berhane N, Betts A (2012) Bench to bedside translation of antibody drug conjugates using a multiscale mechanistic PK/PD model: a case study with brentuximab-vedotin. J Pharmacokinet Pharmacodyn 39:643–659](http://paperpile.com/b/z7MHEw/N90n4)

10. [Khera E, Cilliers C, Bhatnagar S, Thurber GM (2018) Computational transport analysis of antibody-drug conjugate bystander effects and payload tumoral distribution: implications for therapy. Molecular Systems Design & Engineering 3:73–88](http://paperpile.com/b/z7MHEw/BMYGf)

11. [Li JY, Perry SR, Muniz-Medina V, et al (2016) A Biparatopic HER2-Targeting Antibody-Drug Conjugate Induces Tumor Regression in Primary Models Refractory to or Ineligible for HER2-Targeted Therapy. Cancer Cell 29:117–129](http://paperpile.com/b/z7MHEw/HYKeD)

12. [Wiśniewski JR, Vildhede A, Norén A, Artursson P (2016) In-depth quantitative analysis and comparison of the human hepatocyte and hepatoma cell line HepG2 proteomes. J Proteomics 136:234–247](http://paperpile.com/b/z7MHEw/g3zI9)

13. [Shah DK, Betts AM (2012) Towards a platform PBPK model to characterize the plasma and tissue disposition of monoclonal antibodies in preclinical species and human. J Pharmacokinet Pharmacodyn 39:67–86](http://paperpile.com/b/z7MHEw/VG6iu)

14. [Onsum MD, Geretti E, Paragas V, et al (2013) Single-cell quantitative HER2 measurement identifies heterogeneity and distinct subgroups within traditionally defined HER2-positive patients. Am J Pathol 183:1446–1460](http://paperpile.com/b/z7MHEw/Ico67)

15. [Davies B, Morris T (1993) Physiological parameters in laboratory animals and humans. Pharm Res 10:1093–1095](http://paperpile.com/b/z7MHEw/HWeXO)

16. [AstraZeneca Canada Inc. (2021) Enhertu Product Monograph](http://paperpile.com/b/z7MHEw/M3pTK)

17. [Poon KA, Flagella K, Beyer J, et al (2013) Preclinical safety profile of trastuzumab emtansine (T-DM1): mechanism of action of its cytotoxic component retained with improved tolerability. Toxicol Appl Pharmacol 273:298–313](http://paperpile.com/b/z7MHEw/UqZ1b)

18. [FDA (2019) Enhertu Multi-Discipline Review BLA](http://paperpile.com/b/z7MHEw/nxb5b)

19. [Xie H, Audette C, Hoffee M, et al (2004) Pharmacokinetics and biodistribution of the antitumor immunoconjugate, cantuzumab mertansine (huC242-DM1), and its two components in mice. J Pharmacol Exp Ther 308:1073–1082](http://paperpile.com/b/z7MHEw/C9GdY)

20. [Okamoto H, Oitate M, Hagihara K, et al (2020) Pharmacokinetics of trastuzumab deruxtecan (T-DXd), a novel anti-HER2 antibody-drug conjugate, in HER2-positive tumour-bearing mice. Xenobiotica 1–9](http://paperpile.com/b/z7MHEw/KnoSQ)

21. [Shah DK, King LE, Han X, et al (2014) A priori prediction of tumor payload concentrations: preclinical case study with an auristatin-based anti-5T4 antibody-drug conjugate. AAPS J 16:452–463](http://paperpile.com/b/z7MHEw/SFIj9)

22. [Kawato Y, Aonuma M, Hirota Y, et al (1991) Intracellular roles of SN-38, a metabolite of the camptothecin derivative CPT-11, in the antitumor effect of CPT-11. Cancer Res 51:4187–4191](http://paperpile.com/b/z7MHEw/JLHX)

23. [Ogitani Y, Aida T, Hagihara K, et al (2016) DS-8201a, A Novel HER2-Targeting ADC with a Novel DNA Topoisomerase I Inhibitor, Demonstrates a Promising Antitumor Efficacy with Differentiation from T-DM1. Clin Cancer Res 22:5097–5108](http://paperpile.com/b/z7MHEw/pSCU5)

24. [Pfister TD, Reinhold WC, Agama K, et al (2009) Topoisomerase I levels in the NCI-60 cancer cell line panel determined by validated ELISA and microarray analysis and correlation with indenoisoquinoline sensitivity. Mol Cancer Ther 8:1878–1884](http://paperpile.com/b/z7MHEw/usCM)

25. [Wiśniewski JR, Hein MY, Cox J, Mann M (2014) A “Proteomic Ruler” for Protein Copy Number and Concentration Estimation without Spike-in Standards *. Mol Cell Proteomics 13:3497–3506](http://paperpile.com/b/z7MHEw/lNHC)

26. [Wang Y, Rakela S, Chambers JW, et al (2019) Kinetic Study of DNA Topoisomerases by Supercoiling-Dependent Fluorescence Quenching. ACS Omega 4:18413–18422](http://paperpile.com/b/z7MHEw/oAtP)

27. [Simeoni M, Magni P, Cammia C, et al (2004) Predictive pharmacokinetic-pharmacodynamic modeling of tumor growth kinetics in xenograft models after administration of anticancer agents. Cancer Res 64:1094–1101](http://paperpile.com/b/z7MHEw/8iEMn)

28. [van der Lee MMC, Groothuis PG, Ubink R, et al (2015) The Preclinical Profile of the Duocarmycin-Based HER2-Targeting ADC SYD985 Predicts for Clinical Benefit in Low HER2-Expressing Breast Cancers. Mol Cancer Ther 14:692–703](http://paperpile.com/b/z7MHEw/qaMx4)

29. [Haddish-Berhane N, Shah DK, Ma D, et al (2013) On translation of antibody drug conjugates efficacy from mouse experimental tumors to the clinic: A PK/PD approach. J Pharmacokinet Pharmacodyn 40:557–571](http://paperpile.com/b/z7MHEw/02x49)

30. [Lewis Phillips GD, Li G, Dugger DL, et al (2008) Targeting HER2-positive breast cancer with trastuzumab-DM1, an antibody-cytotoxic drug conjugate. Cancer Res 68:9280–9290](http://paperpile.com/b/z7MHEw/mzKcO)

31. [Lee SH, Kim Y-S, Han W, et al (2016) Tumor growth rate of invasive breast cancers during wait times for surgery assessed by ultrasonography. Medicine 95:e4874](http://paperpile.com/b/z7MHEw/vwMxy)

32. [Singh AP, Shah DK (2017) Application of a PK-PD Modeling and Simulation-Based Strategy for Clinical Translation of Antibody-Drug Conjugates: a Case Study with Trastuzumab Emtansine (T-DM1). AAPS J 19:1054–1070](http://paperpile.com/b/z7MHEw/F29sM)

33. [You F, Roberts LA, Kang SP, et al (2008) Low-level expression of HER2 and CK19 in normal peripheral blood mononuclear cells: relevance for detection of circulating tumor cells. J Hematol Oncol 1:2](http://paperpile.com/b/z7MHEw/aDFOh)

34. [Jensen BV, Johansen JS, Price PA (2003) High levels of serum HER-2/neu and YKL-40 independently reflect aggressiveness of metastatic breast cancer. Clin Cancer Res 9:4423–4434](http://paperpile.com/b/z7MHEw/1sYsC)

35. [Perrier A, Gligorov J, Lefèvre G, Boissan M (2018) The extracellular domain of Her2 in serum as a biomarker of breast cancer. Lab Invest 98:696–707](http://paperpile.com/b/z7MHEw/V2O9Y)

36. [Betts A, Clark T, Jasper P, et al (2020) Use of translational modeling and simulation for quantitative comparison of PF-06804103, a new generation HER2 ADC, with Trastuzumab-DM1. J Pharmacokinet Pharmacodyn 47:513–526](http://paperpile.com/b/z7MHEw/MAWK9)

37. [Adams R, Griffin L, Compson JE, et al (2016) Extending the half-life of a fab fragment through generation of a humanized anti-human serum albumin Fv domain: An investigation into the correlation between affinity and serum half-life. MAbs 8:1336–1346](http://paperpile.com/b/z7MHEw/vRwkD)

38. [Girish S, Gupta M, Wang B, et al (2012) Clinical pharmacology of trastuzumab emtansine (T-DM1): an antibody-drug conjugate in development for the treatment of HER2-positive cancer. Cancer Chemother Pharmacol 69:1229–1240](http://paperpile.com/b/z7MHEw/Rvug9)

39. [Doi T, Shitara K, Naito Y, et al (2017) Safety, pharmacokinetics, and antitumour activity of trastuzumab deruxtecan (DS-8201), a HER2-targeting antibody–drug conjugate, in patients with advanced breast and gastric or gastro-oesophageal tumours: a phase 1 dose-escalation study. Lancet Oncol 18:1512–1522](http://paperpile.com/b/z7MHEw/cCf18)

40. [Cao Y, Jusko WJ (2014) Survey of monoclonal antibody disposition in man utilizing a minimal physiologically-based pharmacokinetic model. J Pharmacokinet Pharmacodyn 41:571–580](http://paperpile.com/b/z7MHEw/BcIfV)

41. [Spratt JA, von Fournier D, Spratt JS, Weber EE (1993) Mammographic assessment of human breast cancer growth and duration. Cancer 71:2020–2026](http://paperpile.com/b/z7MHEw/WCwlJ)
