# Supplementary material for: Towards a platform quantitative systems pharmacology (QSP) model for preclinical to clinical translation of antibody drug conjugates (ADCs)
Source: J Pharmacokinet Pharmacodyn. 2023 Oct 3;51(5):429–47. doi: 10.1007/s10928-023-09884-6 (PMC11576657; doi:10.1007/s10928-023-09884-6)
Supplement: Supplementary file 2 — Supplementary file2 (DOCX 1025 kb) [file 10928_2023_9884_MOESM2_ESM.docx]

# **Supplemental Figures** for “Towards a platform quantitative systems pharmacology (QSP) model for preclinical to clinical translation of antibody-drug conjugated (ADCs)”

Journal of Pharmacokinetics/Pharmacodynamics

Bruna Scheuher, Khem Raj Ghusinga, Kimiko McGirr, Maksymilian Nowak, Sheetal Panday, Joshua Apgar, Kalyanasundaram Subramanian and Alison Betts

Corresponding author: Alison Betts, Applied BioMath, 561 Virginia Road, Concord MA 01742
Email: [alisonbetts72@gmail.com](mailto:alisonbetts72@gmail.com)

**SUPPLEMENTARY FIGURE LEGENDS**

**Figure S1A: Schematic representation of the *in vitro* model.**


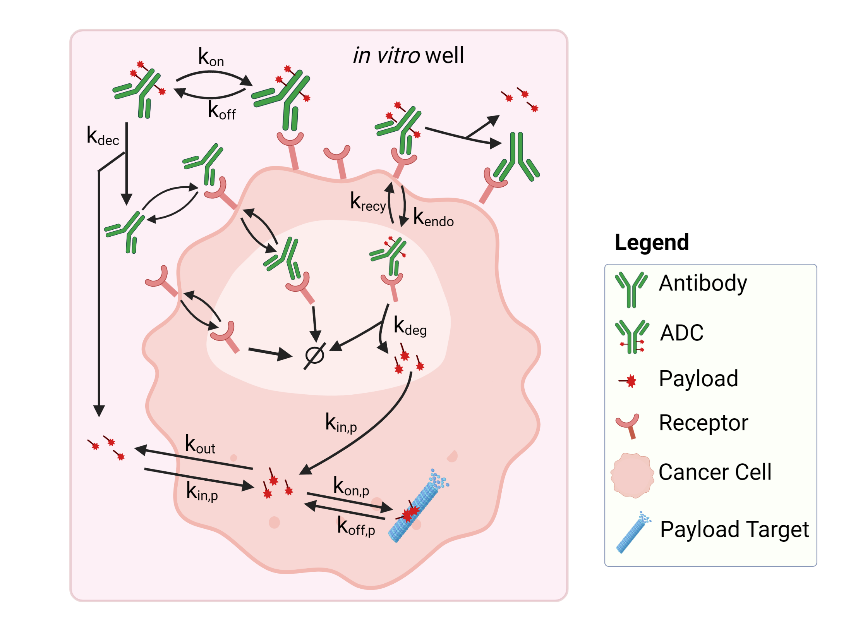


**Figure S1B: Observed (*symbols*) and in vitro model calibrated or predicted (*lines*) trastuzumab internalization data. (A)** Model calibration: 125I- trastuzumab was incubated (gray shaded area) with SK-BR-3 breast cancer cells and cell surface, dissociated, internalized and catabolized radioactivity was measured [1]. **(B)** Model validation: Trastuzumab was incubated with BT-474 cancer cells and internalized trastuzumab was measured [2].

**(A)**

**(B)**
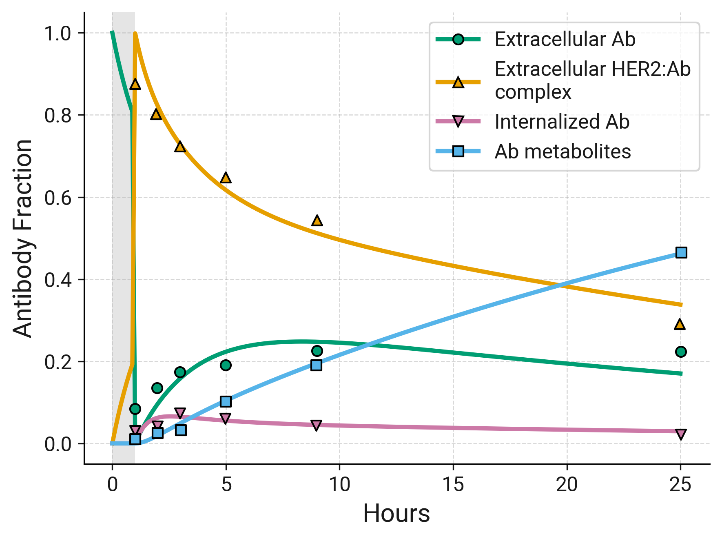


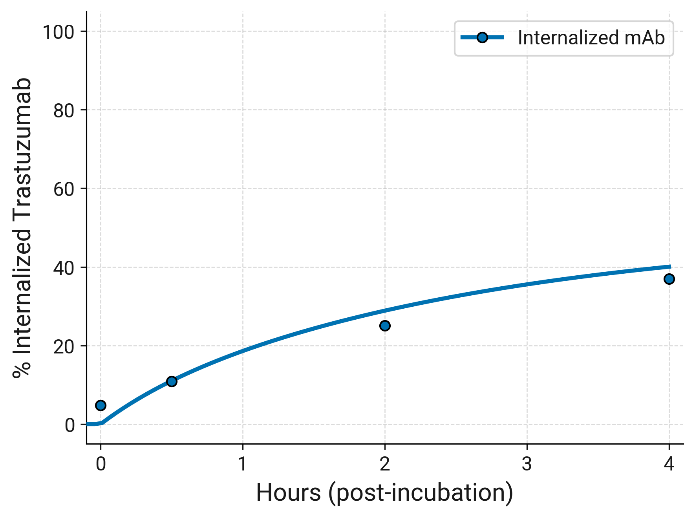


**Figure S2: Observed vs. predicted for in vitro and in vivo calibrations.**

1. **
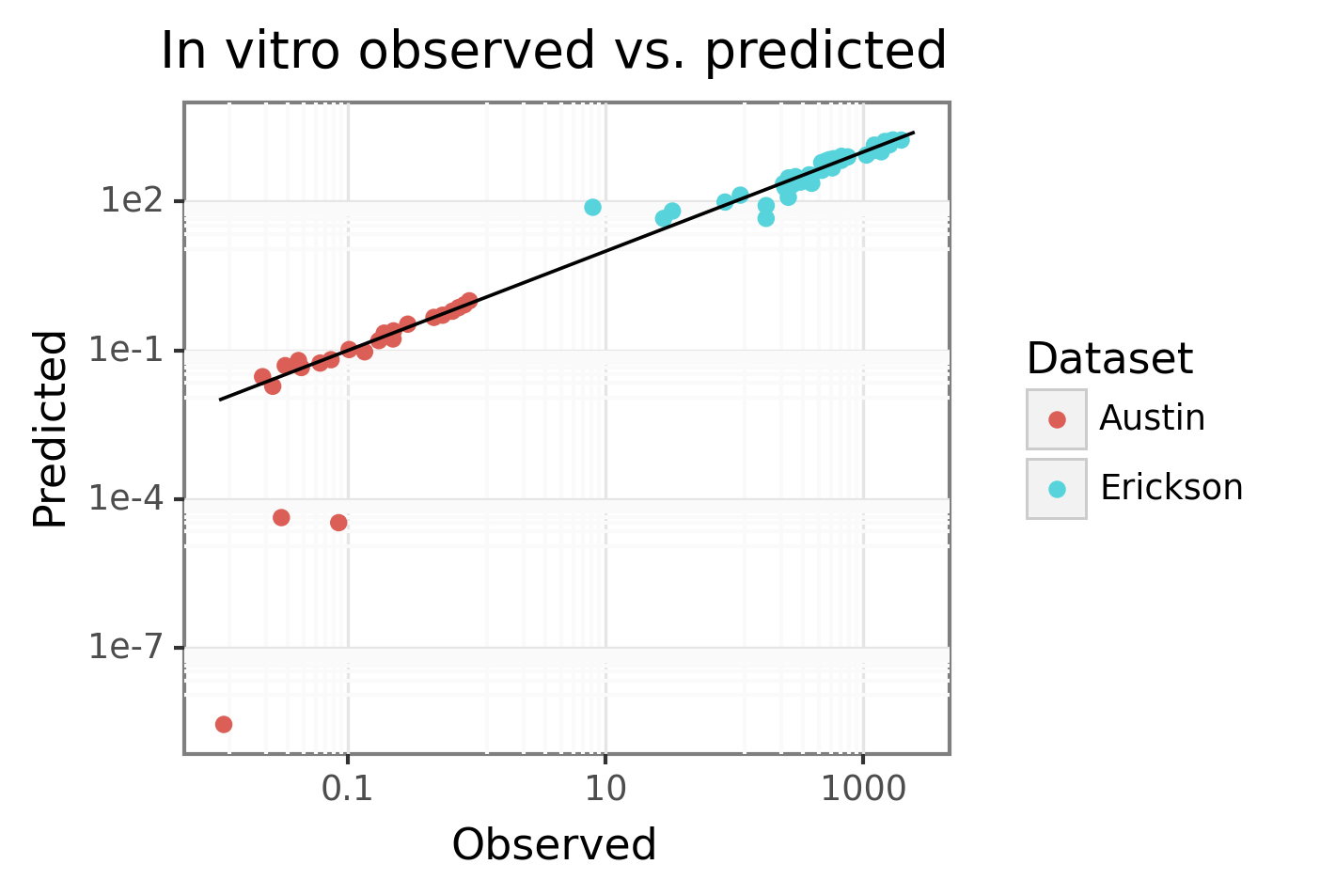
**

**Figure S3: Schematic representation of the mouse model.**


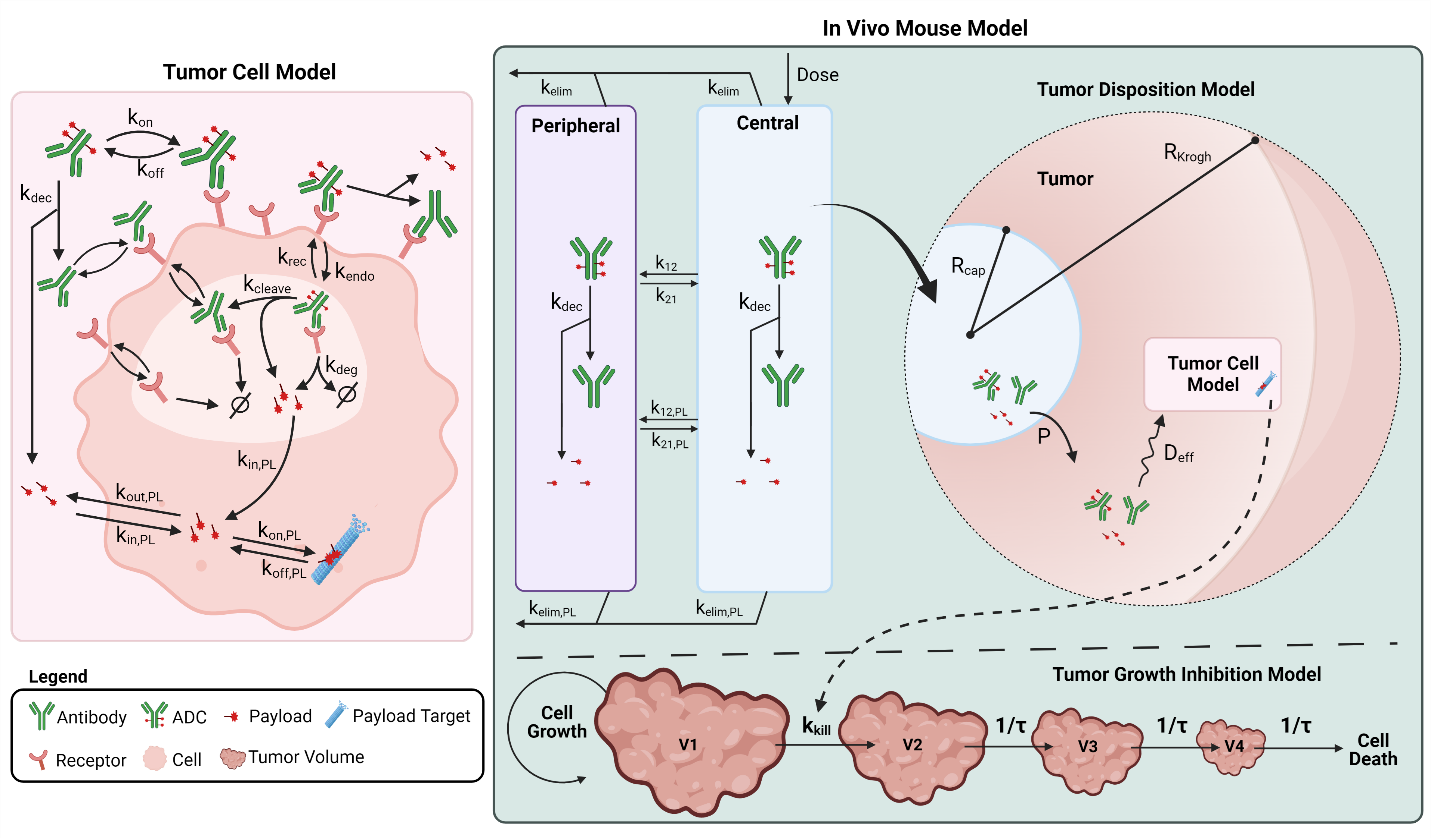


**Figure S4: Observed (*symbols*) and model calibrated (*lines*) plasma PK profiles for ADC and total antibody following single dose IV administration of (A) T-DM1** [[3]](https://paperpile.com/c/2FlyGJ/gOb1N) **and (B) T-DXd** [[4]](https://paperpile.com/c/2FlyGJ/KgMwM) **at 3 mg/kg to non-tumor bearing mice.** The model was used to fit the data for both ADCs simultaneously, and only deconjugation rate was allowed to vary between ADCs.
**Observed (symbols) and model predicted (lines) plasma PK profiles following IV administration of (C) a maytansine with structural similarity to DM1 at 0.1mg/kg** [[5]](https://paperpile.com/c/2FlyGJ/TpPYs) **and (D) DXd at 1 mg/kg to non-tumor bearing mice** [[4]](https://paperpile.com/c/2FlyGJ/KgMwM)**.** The model was used to fit the data for both payloads simultaneously, and only half-life was allowed to vary for each payload.


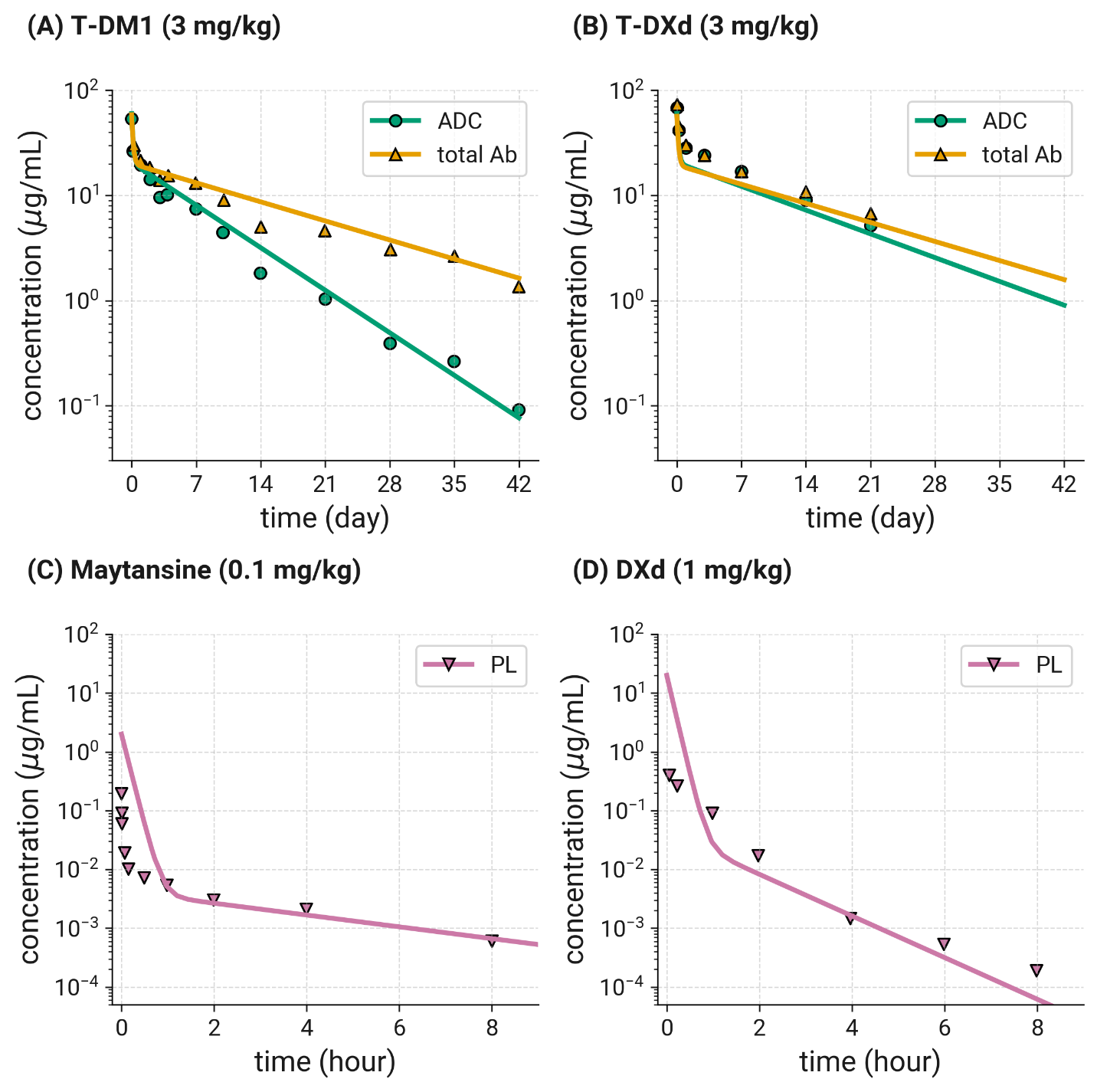


**Figure S5: Observed (*symbols*) and in vivo PK/PD model calibrated (*lines*) tumor growth inhibition by T-DM1 in KPL4 and BT474 xenograft mouse studies.**


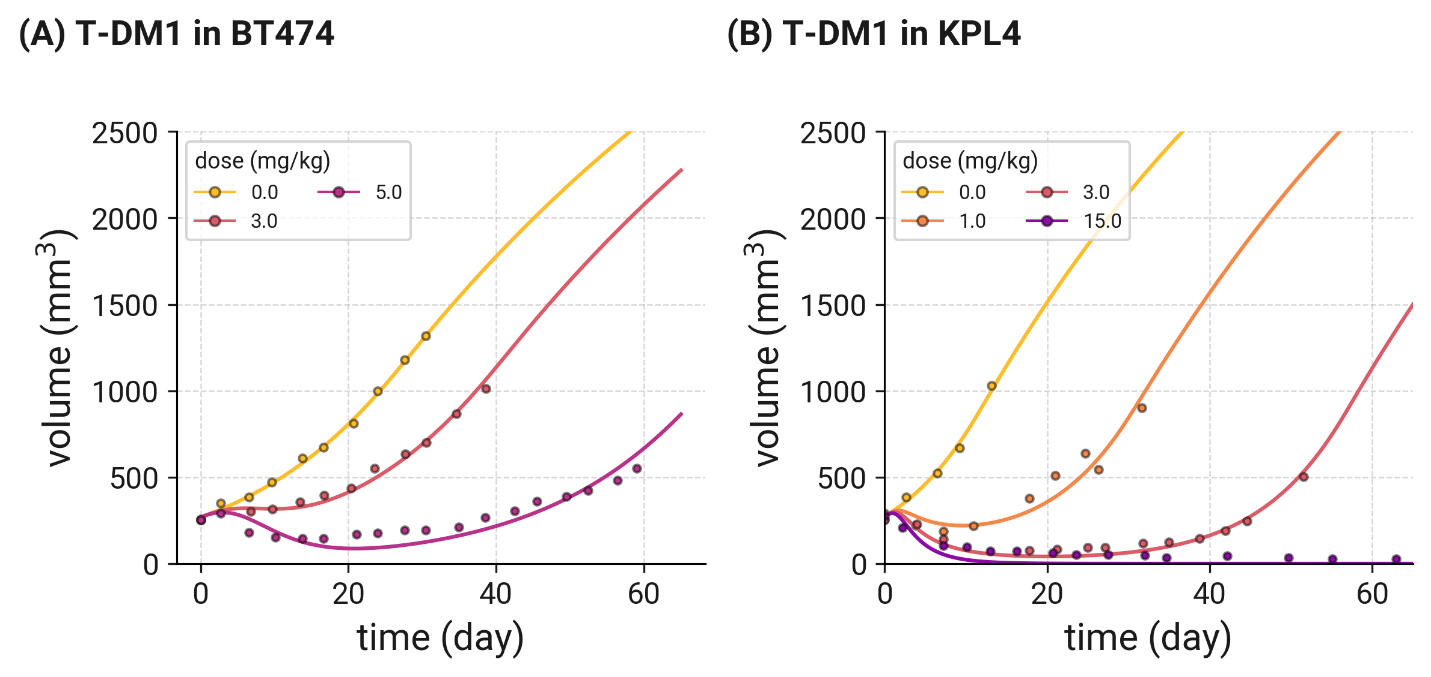


**Figure S6:** **Global sensitivity analysis of Human T-DM1 model parameters.** Latin hypercube partial rank correlation coefficient (LHS- PRCC) was performed to understand which parameters impacted tumor growth volume (summarized by AUC of tumor volume). Parameters shown had a Bonferroni-adjusted p-value > 0.05 and are ordered from greatest to least sensitive based on their PRCC value. Drug parameters (e.g., DAR) were sampled from a linear uniform distribution (red), while most system parameters (e.g., biological parameters) were sampled from a log normal distribution (blue).
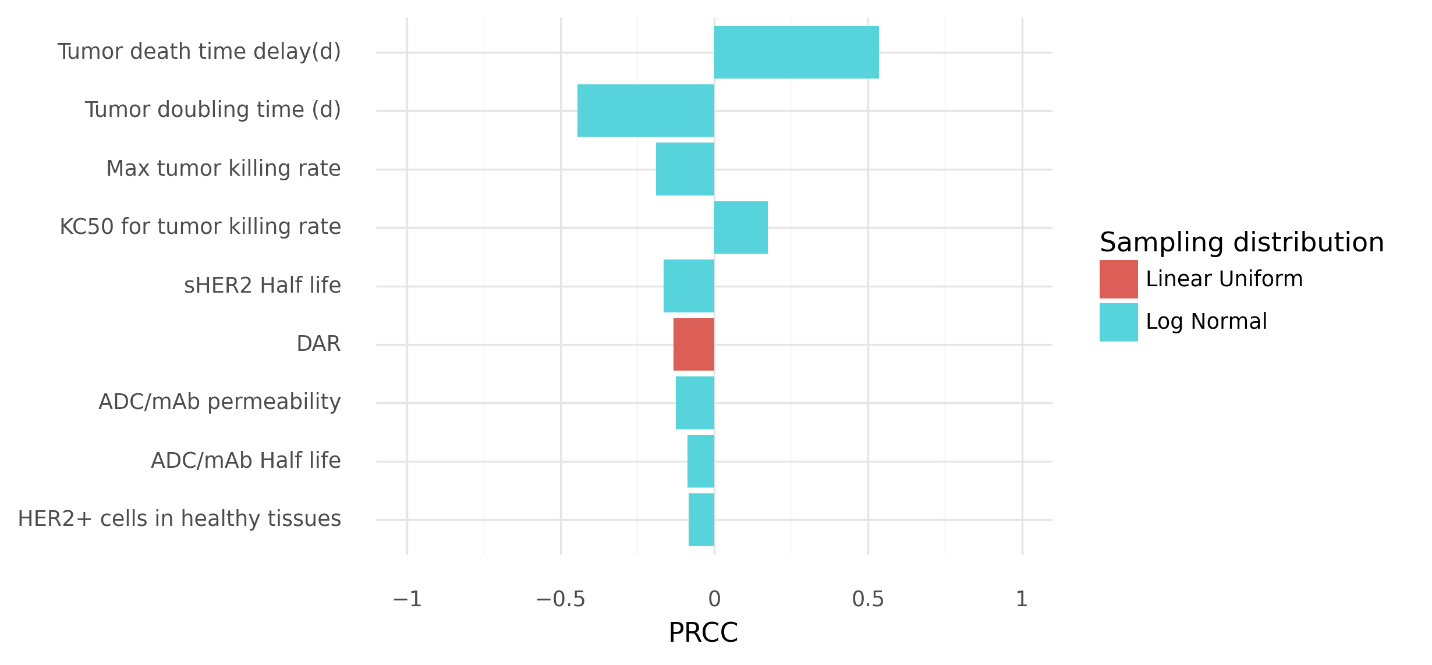


1. Austin CD, De Mazière AM, Pisacane PI, et al (2004) Endocytosis and sorting of ErbB2 and the site of action of cancer therapeutics trastuzumab and geldanamycin. Mol Biol Cell 15:5268–5282

2. Li JY, Perry SR, Muniz-Medina V, et al (2016) A Biparatopic HER2-Targeting Antibody-Drug Conjugate Induces Tumor Regression in Primary Models Refractory to or Ineligible for HER2-Targeted Therapy. Cancer Cell 29:117–129

3. Erickson HK, Lewis Phillips GD, Leipold DD, et al (2012) The Effect of Different Linkers on Target Cell Catabolism and Pharmacokinetics/Pharmacodynamics of Trastuzumab Maytansinoid Conjugates. Mol Cancer Ther 11:1133–1142

4. Okamoto H, Oitate M, Hagihara K, et al (2020) Pharmacokinetics of trastuzumab deruxtecan (T-DXd), a novel anti-HER2 antibody-drug conjugate, in HER2-positive tumour-bearing mice. Xenobiotica 1–9

5. Xie H, Audette C, Hoffee M, et al (2004) Pharmacokinetics and biodistribution of the antitumor immunoconjugate, cantuzumab mertansine (huC242-DM1), and its two components in mice. J Pharmacol Exp Ther 308:1073–1082
